# Laponite vs. Montmorillonite as Eugenol Nanocarriers for Low Density Polyethylene Active Packaging Films

**DOI:** 10.3390/nano14231938

**Published:** 2024-12-02

**Authors:** Achilleas Kechagias, Constantinos E. Salmas, Nikolaos Chalmpes, Areti A. Leontiou, Michael A. Karakassides, Emmanuel P. Giannelis, Aris E. Giannakas

**Affiliations:** 1 Department of Food Science and Technology, University of Patras, 30100 Agrinio, Greece; up1110842@upatras.gr (A.K.); aleontiu@upatras.gr (A.A.L.); 2 Department of Material Science and Engineering, University of Ioannina, 45110 Ioannina, Greece; mkarakas@uoi.gr; 3 Department of Materials Science and Engineering, Cornell University, Ithaca, NY 14850, USA; nc427@cornell.edu

**Keywords:** laponite, montmorillonite, eugenol, LDPE, nanohybrids, active packaging, controlled release, minced pork shelf-life

## Abstract

Although a lot of recent research revealed advantages of novel biopolymers’ implementation as active food packaging polymers, there is not an equivalent effort from industry to use such films, probably because of the required cost to change the supply chain and the equipment. This study investigates the use of two natural abundant nanoclays, laponite (Lap) and montmorillonite (Mt), as eugenol slow-release carriers for enhancing the functionality of low-density polyethylene (LDPE) active packaging films. The target is to combine the spirit of the circular economy with the existent technology and the broadly used materials to develop a novel attractive product for active food packaging applications. Utilizing a vacuum-assisted adsorption method, eugenol was successfully intercalated into Lap and Mt nanoclays, forming EG@Lap and EG@Mt nanohybrids. Testing results confirmed effective integration and dispersion of the nanohybrids within the LDPE matrix. The most promising final film seems to be the LDPE with 15% *w*/*w* EG@Lap nanohybrid which exhibited a higher release rate (k_2_ = 5.29 × 10^−4^ s^−1^) for temperatures ≤70 °C, similar mechanical properties, a significantly improved water barrier (D_wv_ = 11.7 × 10^−5^ cm^2^·s^−1^), and a slightly improved oxygen barrier (Pe_O2_ = 2.03 × 10^−8^ cm^2^·s^−1^) compared with neat LDPE. Antimicrobial and sensory tests on fresh minced pork showed two days’ shelf-life extension compared to pure LDPE and one more day compared to LDPE with 15% *w*/*w* EG@Mt nanohybrid.

## 1. Introduction

Climate change caused by greenhouse gases has catalyzed scientific research in many fields and has driven researchers to develop technologies for a more sustainable world [1,2,3,4,5]. Climate change causes a reduction in arable land leading to food security concerns [1,6,7,8]. In Food Technology, the demand to reduce the carbon dioxide footprint and thus minimize climate change while increasing food security requires a reduction in food waste by developing new preservation technologies that can extend the shelf-life of foods [9,10,11,12,13]. In the direction of a more sustainable world, researchers have replaced chemical additives used as preservation agents with natural bioactive compounds such as essential oils (EOs) and their derivatives, as well as natural extract preservatives [14,15,16,17]. In the food packaging sector, these natural preservatives are incorporated into packages by developing active packaging [18,19,20]. To that end, nanotechnology provides new approaches for the nanoencapsulation of such bioactive compounds in active packaging films that minimize their loss and increase their antioxidant and antibacterial activity [12,21,22,23,24,25]. One of the most used nanoencapsulation technologies of EOs and their derivatives is adsorption on nanocarriers such as nanoclays, natural zeolites, silicas, and activated carbons [26,27,28,29,30,31,32] and the incorporation of such hybrids in packaging films which results in slow and controlled release of EOs [33,34,35,36,37,38]. Nanoclay is a type of nanomaterial composed of layered mineral silicates with particles in the nanometer scale, typically less than 100 nm in at least one dimension [37]. Layered silicates such as montmorillonite (Mt) and laponite (Lap) are the most widely studied 2D nanomaterials because of their low cost and excellent biocompatibility [39]. Among natural nanoclays, halloysite nanotubes (HNTs) and montmorillonite (Mt) are the most common used for EOs control release [40,41]. HNTs have an advantage because of their tubular shape which leads to the slow release of the adsorbed EOs and/or their derivatives, as well as for the mechanical reinforcement of the obtained polymer/HNT nanocomposite matrix [34,42,43]. On the other hand, Mt has the advantage that it can be easily organically modified (OrgMt) and thus adsorb higher amounts of EOs and their derivatives than pure Mt [40,44,45]. Recently, laponite nanoclay (Lap) was studied as an ideal nanocarrier for drug delivery, biomedical, and essential oil adsorption applications [46,47,48,49,50]. Lap is a synthetic layered smectite clay mineral and its crystalline structure is very similar to that of montmorillonite, where an octahedral layer of magnesium cations is bonded with oxygen and hydroxyl groups, sandwiched by two tetrahedral silicate layers, referred to as the 2:1 structure [46,47,51]. Lap has a higher external surface area and higher d-spacing than Mt, while its particles have a discoidal shape with a diameter ranging from 25 to 30 nm [46,47,52]. Recently, Ferreira et al. [47] directly mixed Lavender, tea tree, and rosemary oil with Lap. Thermogravimetric analysis showed that the EO amount absorbed into Lap was more than 240 mg∙g^−1^ for all EOs used. X-ray diffraction analysis showed an increase in the Lap interlayer d-spacing, indicating that EO molecules were successfully intercalated, and forming larger grain aggregates [47]. In contrast, EO adsorption on Mt is usually limited to the external surfaces of Mt and no intercalation takes place [26,30,44].

Eugenol (EG) is a hydroxyphenyl propene, naturally occurring in the essential oils of plants such as clove oil, and is largely used in both foods and cosmetics as a flavoring agent [53]. A large body of recent scientific studies suggest that EG exerts beneficial effects on human health as an antioxidant and anti-inflammatory agent [54,55,56,57,58,59,60,61,62]. It also shows excellent antimicrobial activity against fungi and a wide range of Gram-negative and Gram-positive bacteria [49,50,51]. Even though in the last few years there have been many studies on the adsorption of EO derivatives such as thymol (TO) and carvacrol (CV) in nanoclays to develop antibacterial nanohybrids, to the best of our knowledge none has focused on EG alone [63,64,65,66,67,68]. Recently, Saadat et al. [69] incorporated clove oil which contains EG into HNTs, and the developed clove oil–HNT nanohybrid was further combined with chitosan for the formulation of active food packaging films. The obtained nanohybrids were incorporated into linear low-density polyethylene films to obtain nanocomposite active films with strong antibacterial activity against pathogenic bacteria, and controlled-release properties of thymol, carvacrol, and eugenol [70]. However, no study has focused on the incorporation of pure EG in either unmodified Mt or Lap nanoclays.

Low-density polyethylene (LDPE) is widely used in flexible food packaging films because of its high water barrier properties, good tensile properties, and tear resistance up to −60 °C [71,72,73,74]. Because of its excellent thermomechanical properties, LDPE can be used in the production of flexible packaging films via hot-extrusion and hot-blowing processes [75]. It is quite resistant to acids, bases, salt solutions, and usual organic solvents up to a temperature of 60 °C [73]. The ability to produce bio-polyethylene (bio-PE) which is polyethylene made from ethylene derived from non-fossil fuels such as bioethanol makes LDPE a material that can be used in the future in food packaging [74,76,77].

This study presents the development and characterization of novel EG@Lap and EG@Mt nanohybrids and novel LDPE/xEG@Lap and LDPE/xEG@Mt hybrid (where x = 5, 10, and 15 wt.%) active films. The two hybrids are studied and their performance in active packaging films compared and discussed.

## 2. Materials and Methods

### 2.1. Materials

The LDPE used was offered by the Mantzaris plastic film company (Tsakiri, Zevgolatio, Korinthia, 200 06, Greece). Eugenol, 2-Methoxy-4-(2-propenyl)phenol, 4-Allyl-2-methoxyphenol, and 4-Allylguaiacol with CAS No. 97-53-0 were purchased from Sigma-Aldrich (Darmstadt, Germany). Lap (laponite) with a cation exchange capacity (CEC) of 50 meq per 100 g was purchased from Southern Clay Products Inc. Mt with CAS No. 1318-93-0 30 and CEC 30 meq/100 g was purchased from Sigma-Aldrich (Darmstadt, Germany). 2,2-diphenyl-1-picrylhydrazyl (DPPH) (CAS No. 1898-66-4) was purchased also from Sigma-Aldrich (Darmstadt, Germany).

### 2.2. Preparation of EG@Lap and EG@Mt Nanohybrids

**Figure 1:**
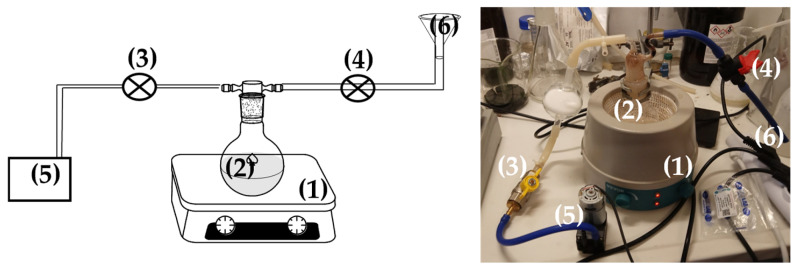
Schematic presentation (**left** part) and image (**right** part) of handmade apparatus used for the preparation of the EG@Lap and EG@Mt nanohybrids: (1) stirrer with a heating plate, (2) spherical glass flask, (3) security valve of the pump, (4) security valve of the CV tank, (5) air vacuum pump, and (6) CV tank. CV: carvacrol and NZ: natural zeolite.

For the preparation of both EG@Lap and EG@Mt nanohybrids, the vacuum-assisted adsorption process was followed. Specifically, 3 g of pure Lap or Mt were placed in a spherical glass flask and heated for 15 min at 100 °C under vacuum (10^−3^ bar, see Figure 1). Under these conditions, all the adsorbed water was removed from the clay. After the adsorbed water was removed, laponite changed its color from light white to grey while Mt turned from light beige to grey beige. After the end of the drying process, the security valve of the pump was closed, and the safety valve of the EG tank was opened to allow the EG to be adsorbed dropwise and under-stirred into the glass spherical flask (see Figure 1). The EG@Lap and EG@Mt nanohybrids were removed from the glass flask and kept at 25 °C at 50%RH for further characterization and use.

### 2.3. Preparation of LDPE/xEG@Lap and LDPE/xEG@Mt Active Films

LDPE/xEG@Lap and LDPE/xEG@Mt active films were made using a twin-screw mini lab extruder (Haake Mini Lab II, Thermo Scientific, ANTISEL, S.A., Athens, Greece). The twin-screw extruder rotation was adjusted at 100 rpm, while the temperature was kept at 200 °C; the extrusion time was 3 min. Appropriate amounts of LDPE pellets were mixed with 0.25, 0.50, and 0.75 g of EG@Lap or EG@Mt to achieve a final EG@Lap or EG@Mt nominal content of 5, 10, and 15 wt.%, respectively. For comparison, pure LDPE was also extruded under the same conditions and referred to as the “blank” sample. The extruded pellets (LDPE/xEG@Lap and LDPE/xEG@Mt) were converted to LDPE/xEG@Lap and LDPE/xEG@Mt active films with 10 cm average diameter and 0.1 mm average thickness by heat pressing the pellets at 110 °C with 1 tn pressure for 2 min (Specac Atlas™ Series Heated Platens, Specac, Orpington, UK). All obtained films were kept in a desiccator under 25 °C temperature and relative humidity of 50% RH before any testing. The sample names LDPE, EG@Lap, and the EG@Mt as well as the twin-screw extruder operating conditions employed for the preparation of all LDPE/xEG@Lap and LDPE/xEG@Mt active films are listed in Table 1 for comparison.

### 2.4. Physicochemical Characterization of EG@Lap and EG@Mt Nanohybrids and LDPE/xEG@Lap and LDPE/xEG@Mt Active Films

The EG@Lap and EG@Mt nanohybrids, pure Lap and Mt, as well as all obtained LDPE/xEG@Lap and LDPE/xEG@Mt active films and pure LDPE film were characterized with X-Ray Diffraction (XRD) analysis using a Bruker XRD D8 Advance diffractometer (Bruker, Analytical Instruments, S.A., Athens, Greece) equipped with a LINXEYE XE High Resolution Energy Dispersive detector. The diffractometer was thermostated at 20 °C, and the beam monochromator was operated at a voltage of 40 kV and a beam current of 40 mA. The CuKα radiator worked in 1-D mode with a wavelength λ = 1.541874 Å. Scanning parameters were set as follows: two theta range 0.5°–30°, increment 0.03°, PSD 0.764, counting time 1022 s, and slit width 0.6 mm. FTIR spectra were recorded using an FT/IR-6000 JASCO Fourier-transform spectrometer (JASCO, Interlab, S.A., Athens, Greece). Measurements were carried out using the KBr (0.5%wt. to 1%wt.) tablet technique. The spectra recorded over the wavenumber range from 4000 to 400 cm^−1^ at a resolution of 4 cm^−1^ and 64 scans were averaged to reduce noise.

### 2.5. EG Release Kinetic Studies of EG@Lap and EG@Mt Nanohybrids

To determine the total EG amount adsorbed on Lap or Mt, desorption kinetic experiments were carried out for both EG@Lap and EG@Mt nanohybrids, using a moisture analyzer AXIS AS-60 (AXIS Sp. z o.o., ul. Kartuska 375b, 80–125 Gdańsk, Poland). Approximately 100 mg of each nanohybrid was placed in the moisture analyzer and its weight was recorded as a function of time (m_t_). Measurements were taken at 70, 90, and 110 °C. The desorption kinetic experiments were repeated thrice. From the mt vs. t measurements, the normalized values of the fraction q_t_ = (1 − m_t_/m_0_) were calculated and plotted as a function of time. The plots were fitted using the well-known pseudo-second-order adsorption-desorption equation [78,79]. For process order n = 2, the overall normalized mass balance is given by:

(1) $$\frac{{dq}_{t}}{dt}=k_{2}*{\left(q_{e}-q_{t}\right)}^{2}$$

where k_2_ is the rate constant of the pseudo-second-order kinetic model (s^−1^), qt is the desorbed fraction capacity at time t, q_e_ = (1 − m_e_/m_0_) is the maximum desorbed fraction capacity at equilibrium, m_0_ is the initial EOs loading into the nanohybrid, and mt is the EOs amount remaining in the nanohybrid at time t. By integrating Equation (1), we achieve the pseudo-second-order kinetic model:

(2) $$q_{t}=(1-\frac{m_{t}}{m_{0}})=\frac{q_{e}^{2}*k_{2}*t}{q_{e}*k_{2}*t+1}$$

The initial release rate can be computed via Equation (1) and for t = 0 (i.e., q_t_ = 0). Thus:

(3) $$r_{i}={\left.\frac{{dq}_{t}}{dt}\right|}_{t=0}=k_{2}*q_{e}^{2}$$

From the best-fitted plots, the k_2_ and q_e_ values were calculated. Using the estimated k_2_ parameter, the ln(k_2_) term was calculated and plotted as a function of (1/T) to determine the desorption energy (Ε^0^_des_) according to the Arrhenius equation and the theory presented in detail in [80,81,82]:

(4) $$k_{2}=k_{0}\cdot e^{-\frac{E_{des}^{0}}{R\cdot T}}$$

and its linear transformed type:

(5) $$ln\left(k_{2}\right)=ln\left(k_{0}\right)-\frac{E_{des}^{0}}{R\cdot T}$$

where k_2_ is the rate constant of the pseudo-second order kinetic model (s^−1^), Ε^0^_des_ is the desorption activation energy, and A is the Arrhenius constant.

### 2.6. Morphological Characterization of EG@Lap and EG@Mt Nanohybrids and LDPE/xEG@Lap and LDPE/xEG@Mt Active Films

Scanning electron microscopy (SEM) images of EG@Lap and EG@Mt nanohybrids as well as pure Lap and Mt nanoclays were acquired using a Zeiss Gemini 500 SEM at a low accelerating voltage of 3 kV to reduce the excitation volume and enhance resolution. The images were captured using an in-lens secondary electron detector, and the powders were mounted on an aluminum holder with double-sided adhesive carbon tape. In case of LDPE/xEG@Lap and LDPE/xEG@Mt active films, as well as pure LDPE film, the SEM study was conducted at a low accelerating voltage of 1.5 kV to prevent film degradation.

### 2.7. Tensile Properties of LDPE/xEG@Lap and LDPE/xEG@Mt Active Films

Tensile properties of active films as well as neat LDPE film were determined according to the American Society for Testing and Materials (ASTM) D638 method, by employing a Shimadzu AG-Xplus (5 kN), instrument (Shimadzu, Kyoto, Japan). Three to five dog-bone-shaped samples were tensioned, and the stress-strain values were recorded. Three to five samples of type IV ASTMD638 specimens of each film were tensioned at an across-head speed of 2 mm/min. Film thickness was measured by using a Total 321501 digital thickness gauge with an accuracy of ±0.1 mm. Such measurements were applied to 3 different points on the dog-bone-shaped film and the average thickness was adopted for each sample. By using the applicable software (Trapezium X version 1.5.6, Shimadzu, Kyoto, Japan) with the recorded force (N), the deformation (mm) measurements, and the gauge dimensions, the mean elastic modulus (E), the ultimate strength (σ_uts_), and the % elongation at break (%ε) were calculated.

### 2.8. Water/Oxygen Barrier Properties of LDPE/xEG@Lap and LDPE/xEG@Mt Active Films

#### 2.8.1. Water Barrier Properties

Water barrier properties of active films as well as neat LDPE film were measured with a handmade apparatus according to the ASTM E96/E 96M-05 method at 38 °C and 95% RH by following the methodology described in detail elsewhere [41]. The Water Vapor Transmission Rate (WVTR) values were transformed to a water vapor diffusion coefficient (D_wv_) by using Fick’s law and the following equation:

(6) $$D_{WV}=WVTR\cdot \frac{Dx}{DC}$$

where WVTR [g/(cm^2^·s)] is the water vapor transmission rate, Dx (cm) is the film thickness, and DC (g/cm^3^) is the humidity concentration gradient on the two opposite sides of the film.

#### 2.8.2. Oxygen Barrier Properties

Oxygen barrier properties were determined according to ASTM D 3985 at 23 °C and 0%RH using an oxygen permeation analyzer (O.P.A., 8001, Systech Illinois Instruments Co., Johnsburg, IL, USA). Three disk shape films with an average diameter of 10 cm were placed in the sample area of the analyzer and the oxygen transmission rate (OTR, cc/m^2^/day) values were measured. The average thickness of each sample was measured after the end of OTR measurement by measuring the thickness at fifteen different points of the film. The oxygen diffusion coefficient value for each sample was calculated using the methodology described in detail elsewhere [41].

### 2.9. In Vitro Antioxidant Activity Determination of LDPE/xEG@Lap and LDPE/xEG@Mt Active Films

For all experiments, 250 mL of 2.16 mM (mmol/L) DPPH radical methanolic standard solution was used. For the calibration curve, five DPPH radical methanolic solutions with 10, 20, 30, 40, and 50 mg/L were prepared by diluting the appropriate amounts of DPPH radical standard solution and their absorbance was measured at 517 nm using a Shimadzu UV-1280 UV/VIS Spectrometer. The calibration curve (see Supplementary Materials) of absorbance (y) versus the concentration (x) of [DPPH^•^] free radical was expressed by the following equation:

y = 0.0388x + 0.015; R^2^ = 0.9994 (7)

For the determination of the concentration required to obtain a 50% antioxidant effect (EC_50_) from all films, 10, 20, 30, 40, and 50 mg of granule film were placed in dark vials in triplicates. An amount of 3 mL of DPPH radical methanolic solution and 2 mL of acetate buffer 100 mM (pH = 7.10) was added to each vial, and the absorbance of the reaction mixture was measured at 517 nm after 8 h. For a blank sample, we used a vial containing 3 mL of DPPH radical methanolic solution and 2 mL of acetate buffer without the addition of any granule film. The % inhibition of DPPH radical was calculated using the following equation:

(8) $$\%\;scavenged\;{DPPH}^{•}\;at\;steady\;state=\frac{A_{0}^{517}-A_{sample}^{517}}{A_{0}^{517}}\times 100$$

### 2.10. EG Release Kinetics of LDPE/xEG@Lap and LDPE/xEG@Mt Active Films

Release kinetics of EG from all samples were determined by using a moisture analyzer AXIS AS-60 (AXIS Sp. z o.o. ul. Kartuska 375b, 80–125 Gdańsk, Poland). For each film, three to five samples 500 to 700 mg of each film was placed inside the moisture analyzer, and its mass was monitored by heating at 70 °C for 1 h. From the mass loss values (m_t_) as a function of time (t), the mean values of initial EG release rate (RR_EG,initial_) were calculated. Finally, the rate constant of the pseudo-second order kinetic model k_2_ (s^−1^), and the desorption capacity at equilibrium q_e_ s were calculated by fitting the obtained mt and t values with the pseudo-second-order kinetic equation described above (2).

### 2.11. Packaging Preservation Test of Fresh Minced Pork Wrapped with LDPE/15EG@Lap and LDPE/15EG@Mt Active Films and Pure LDPE Film

#### 2.11.1. Packaging Preservation Test of Minced Pork Meat

For the packaging preservation test of minced pork meat, LDPE/15EG@Lap and LDPE/15EG@Mt films were selected and used as the optimum choise. Minced pork meat was offered by the Aifantis meat processing company within one hour of slaughter. The minced pork meat was from the hind pork leg. The minced pork meat was separated into portions of approximately 80–100 g each. Each portion was aseptically wrapped between two disk-shaped films of LDPE/15EG@Lap and LDPE/15EG@Mt and placed inside the Aifantis company’s commercial wrapping paper without the inner film. As a control, a portion of the minced pork meat was aseptically wrapped with two neat LDPE films and placed inside the Aifantis company’s commercial wrapping paper without the inner film. For all tests, samples for the second, fourth, sixth, eighth, and tenth day of preservation were prepared and stored under dark refrigerator conditions at 4 ± 1 °C (LG GC-151SA, Weybridge, UK). Then, total viable count, sensory analysis, pH analysis, and colorimetry analysis were measured periodically during the ten days of storage.

#### 2.11.2. Total Viable Count (TVC) of Minced Pork Meat

For the determination of total viable count (TVC) for all samples, a recently described methodology was followed [27].

#### 2.11.3. Sensory Analysis of Minced Pork Meat

Meat color, odor, and texture during the 10 days of storage were evaluated periodically via sensory analysis and ranked from 0 (lowest degree of each characteristic in the tested samples) to 5 (highest degree of each characteristic in the tested samples) by seven experienced panelist members of the Department of Food Science and Technology experienced in meat sensory evaluation using the conventional descriptive analysis [83,84,85].

#### 2.11.4. pH Analysis of Minced Pork Meat

The pH values of the minced pork meat were measured using a portable pH meter fitted with a penetration electrode and a temperature sensor (pH-Star, Matthäus GmbH, Poettmes, Germany) by following the procedure described in detail recently [24]. To ensure accuracy and reliability, the procedure was conducted in triplicate, and for each treatment group, ten separate pH readings were taken.

#### 2.11.5. Lab* Analysis

The alterations in the CIELAB color parameters (L*, a*, and b*) of the minced pork meat over a period of 10 days of storage were assessed using a LS171 colorimeter from the Linshang Company. Color evaluations were conducted directly on the surface of the minced pork meat, with each treatment group comprising three separate portions. For each of these portions, five discrete readings were taken to capture a robust assessment of the color. The total color differences (ΔE) were calculated using the following equation:

(9) $$\Delta E=\sqrt{{\left(L^{*}-L_{0}^{*}\right)}^{2}+{\left(a^{*}-a_{0}^{*}\right)}^{2}+{\left(b^{*}-b_{0}^{*}\right)}^{2}}$$

In this equation, L*0, a*0, b*0 denote the initial color parameters of the minced pork meat at Day 0 post-treatment. L*, a*, b* represent the respective color parameters at different time points during the 10 days of storage at 4 °C.

### 2.12. Statistical Analysis

All data acquired from structural and mechanical properties measurements, along with, antioxidant activity, total viable count, sensory analysis, pH analysis, and colorimetry analysis, were subjected to statistical analysis to indicate any statistical differences. The Kruskal–Wallis non-parametric statistical test method was chosen to evaluate the significance of difference between the properties’ mean values. Assuming a significance level of *p* < 0.05, all measurements were conducted using three separate samples of each LDPE, LDPE/xEG@Lap, and LDPE/xEG@Mt film. Statistical analysis was performed using SPSS software (v. 28.0, IBM, Armonk, NY, USA).

## 3. Results

### 3.1. Physicochemical Characterization of EG@Lap and EG@Mt Nanohybrids

**Figure 2:**
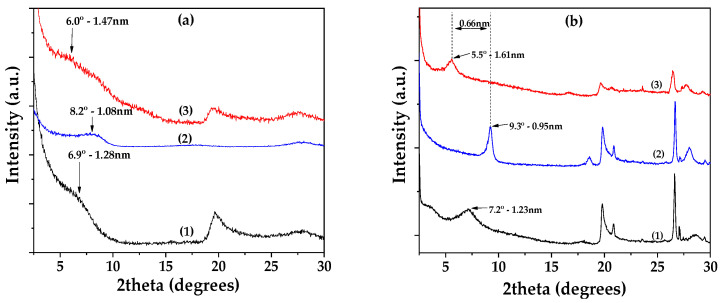
(**a**) XRD plots of (1) pure Lap, (2) dried Lap, and (3) EG@Lap nanohybrid; (**b**) XRD plots of (1) pure Mt, (2) dried Mt, and (3) EG@Mt nanohybrid.

Figure 2a shows the XRD patterns of Lap as received (bottom), dried Lap (middle), and modified EG@Lap nanohybrid (top). The XRD pattern of the as received Lap shows the characteristic reflection of Lap’s basal space at 2theta = 6.9° which corresponds to a d-spacing of 1.28 nm. After the vacuum desorption process, the d-spacing of dried Lap decreases to 1.08 nm. The decrease is consistent with the removal of water molecules from the interlayer of laponite. In EG@Lap nanohybrid, a broad shoulder can be seen at ~6.0°. While it is difficult to assign a single d-spacing from this broad peak, it is clear that it is higher from the dry or even the pristine laponite and consistent with the intercalation of EG in the galleries of Lap.

Figure 2b shows the corresponding patterns for montmorillonite. The XRD pattern of the as-received Mt shows a d-spacing of 1.23 nm which decreases to 0.93 nm after drying. The latter corresponds to collapsed layers suggesting that vacuum-assisted drying successfully removed all the adsorbed water molecules. In the modified EG@Mt nanohybrid, the characteristic reflection of Mt’s basal space is at 2theta = 5.5° corresponding to 1.61 nm interlayer space and consistent with a nanohybrid with intercalated EG molecules.

Thus, both EG@Lap and EG@Mt nanohybrids display intercalated EG molecules in the interlayer spaces. The relatively small particle size of laponite tends to lead to a less structured, turbostratic morphology with a less well-defined d-spacing. Considering that the size of EG molecule lying flat between the layers is equal to that of phenol (0.4 nm), the observed spacings are consistent with such a potential configuration.

Consistent with the above, the FTIR spectra (see Figure S1 in Supplementary Materials) of EG@Lap and EG@Mt (top) show the characteristic peaks of both EG (bottom) and either Lap or Mt (middle) suggesting that the intercalation is the result of simple physisorption with no chemical interactions between EG molecules and either clay.

**Figure 3:**
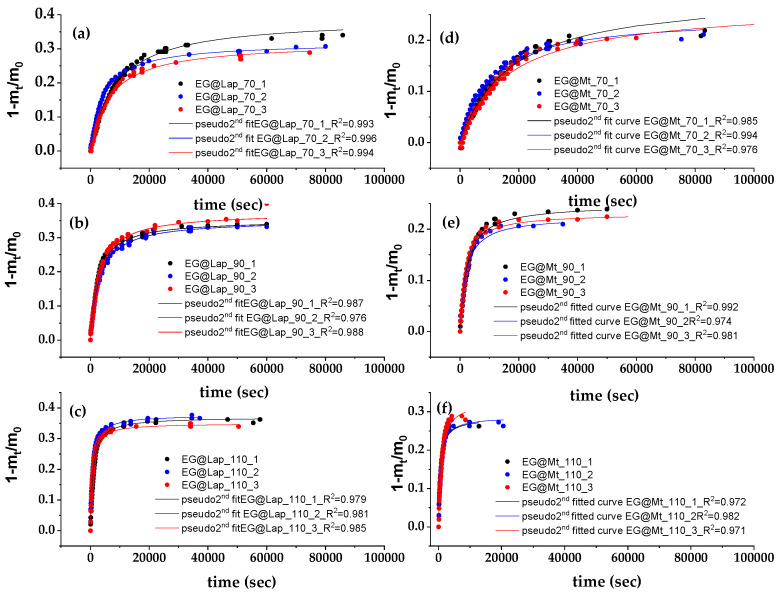
EG desorption isotherm kinetic plots (in triplicates) for EG@Lap (**left** part (**a**–**c**) plots) and EG@Mt (**right** part (**d**–**f**) plots) nanohybrids at 70 °C ((**a**,**d**) plots), 90 °C ((**b**,**e**) plots), and 110 °C ((**c**,**f**) plots). The red line shows the simulation plots according to the pseudo-second-order kinetic model.

Figure 3 shows the recorded values of (1 − m_t_/m_0_) as a function of time (t) for EG@Lap and EG@Mt nanohybrids (in triplicate) at 70, 90, and 110 °C.

These plots were fitted with the pseudo-second-order kinetic model to calculate the k_2_ and q_e_ values according to Equation (1) which are listed in Table 2.

As seen in Table 2, the calculated k_2_ and q_e_ mean values of the EG@Lap nanohybrid are higher than the calculated k_2_ and q_e_ mean values for the EG@Mt nanohybrid. Increasing the temperature leads to higher mean values for k_2_ and q_e_ for both EG@Lap and EG@Mt nanohybrids. The amount of desorbed EG at 70, 90, and 110 °C is 33.8, 35.6, and 36.5%, respectively, for EG@Lap. The corresponding values are 23.5, 26.4, and 29.9% for EG@Mt. The higher q_e_ mean values for EG@Lap compared to EG@Mt suggest that the desorbed EG amount is higher in the former. On the other hand, higher k_2_ values mean that the EG molecules are released faster. Overall, EG@Lap nanohybrid desorbs higher EG amounts with higher initial and total rates than EG@Mt nanohybrid. This is expected because a significant amount of EG molecules are adsorbed on the external surfaces of Lap. The latter is consistent with the less well-defined XRD pattern showing a turbostratic morphology [47,48].

**Figure 4:**
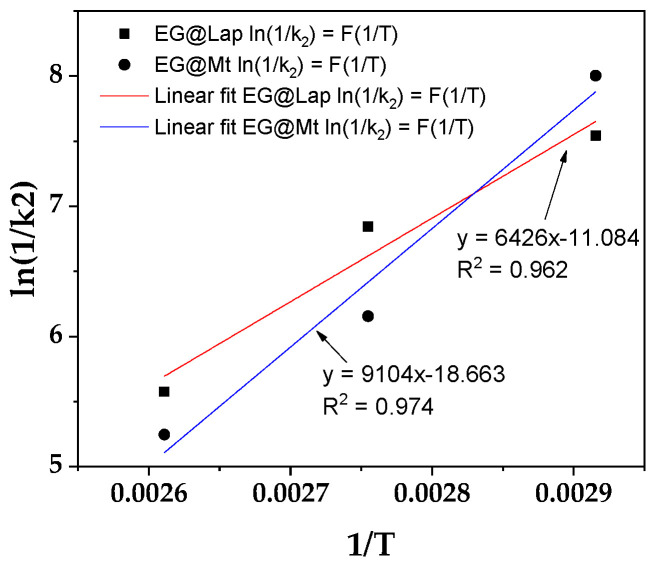
Plot of ln(1/k_2_) values as a function of (1/T) for both EG@Lap and EG@Mt nanohybrids.

From the calculated k_2_ values, the ln(1/k_2_) values were plotted as a function of (1/T) for both EG@Lap and EG@Mt nanohybrids (Figure 4).

From the linear fitted plots in Figure 4, the calculated slope was used with Equations (2) and (3) to determine the EG desorption energy (E^0^_des_). The values are 12.6 and 17.8 Kcal/mol for EG@Lap and EG@Mt, respectively. The values are consistent with the results above. In the case of EG@Lap the EG molecules tend to be more on the external surfaces of the clay and therefore a lower desorption activation energy value is seen.

**Figure 5:**
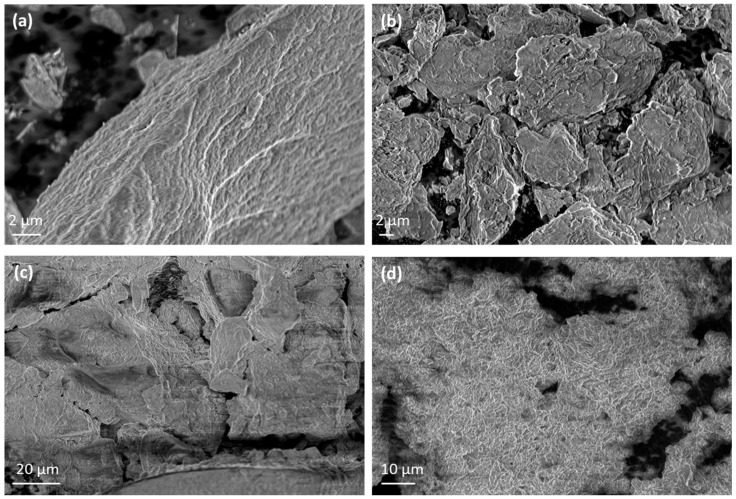
Representative SEM images of (**a**) pure Lap, (**b**) pure Mt, (**c**) EG@Lap, and (**d**) EG@Mt.

The morphological characteristics of pure Lap and Mt along with the corresponding nanohybrids are presented in Figure 5. Representative images of pure Lap and Mt (Figure 5a and Figure 5b, respectively) reveal an irregular disk-like or platelet morphology with obvious lamellar structure for both cases, which is typical feature of clays [86,87]. Larger platelets or clusters of Lap and Mt are presented revealing their tendency to stack or overlap due to interlayer forces and particle–particle interactions.

The SEM images of EG-modified nanohybrids revealed significant changes in morphology compared to the unmodified clays. The surface of clay nanoplatelets is more compact and smoother due to the intercalation of EG molecules within the clay layers. The characteristic flaky or plate-like structure of the Lap and Mt is less pronounced, with a tendency toward a denser packing of clay flakes. The EG intercalation leads to a more uniform appearance, with fewer visible pores indicating successful adsorption between eugenol and the clay particles.

### 3.2. Physicochemical Characterization of LDPE/xEG@Lap and LDPE/xEG@Mt Active Films

**Figure 6:**
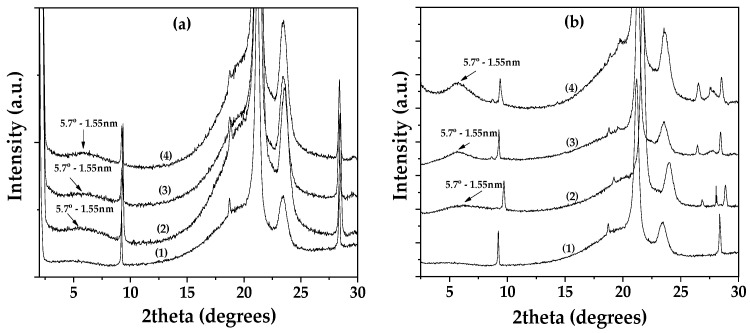
(**a**) XRD plots of (1) pure LDPE film, (2) LDPE/5EG@Lap active film, (3) LDPE/10EG@Lap active film, and (4) LDPE/15EG@Lap active film; (**b**) XRD plots of (1) pure LDPE film, (2) LDPE/5EG@Mt active film, (3) LDPE/10EG@Mt active film, and (4) LDPE/15EG@Mt active film.

Figure 6 shows the XRD patterns for LDPE/xEG@Lap active films (top left) and LDPE/xEG@Mt active films (top right). The corresponding FTIR spectra are on bottom left and bottom right, respectively.

The d-spacing of the films is more or less similar to the EG intercalated hybrids suggesting that no intercalation of the LDPE polymer chains inside the EG@Lap or EG@Mt nanohybrids takes place and that both EG@Lap and EG@Mt nanohybrids are dispersed in the LDPE matrix.

Figure S2 shows the FTIR spectra of pure LDPE film (bottom spectrum in the series) and those of LDPE/xEG@Lap and LDPE/xEG@Mt active films containing varying amounts of EG@clay hybrids. In the FTIR spectrum of pure LDPE film, the bands centered at 2850–2950 can be attributed to the CH_2_ symmetric and asymmetric stretching, respectively, while the peaks located at 1460 and 715 cm^−1^ are ascribed to the bending and rocking deformation of the CH_3_ and CH_2_ groups of LDPE [82]. For all the films consisting of either Lap or Mt, the broad peak at 1050–1100 cm^−1^ is attributed to the Si-O-Si stretching vibration due to the presence of clay particles.

In both LDPE/xEG@Lap and LDPE/xEG@Mt active films, no shifting of the LDPE characteristic peaks is seen implying good compatibility but no specific interactions of both EG@Lap and EG@Mt nanohybrids with the LDPE chains. The results agree with previous reports where EO modified halloysite, natural zeolite, activated carbon, and mesoporous silica SBA-15 were dispersed in LDPE [28,29,68,88].

**Figure 7:**
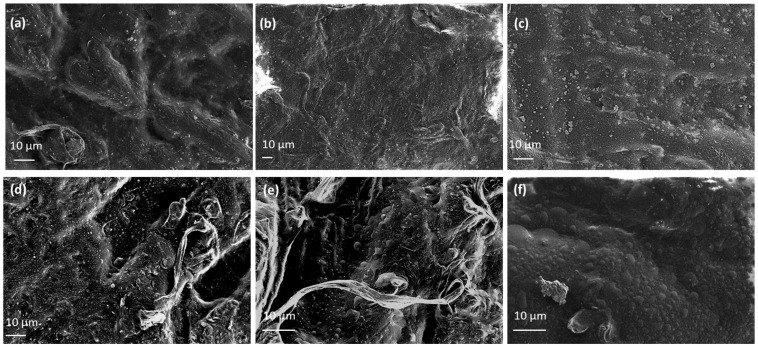
SEM images of (**a**) LDPE/5EG@Lap, (**b**) LDPE/10EG@Lap, (**c**) LDPE/15EG@Lap, (**d**) LDPE/5EG@Mt, (**e**) LDPE/10EG@Mt, and (**f**) LDPE/15EG@Mt active films.

Representative SEM images of EG@Lap incorporated into LDPE show homogeneous dispersion of clay platelets within the polymer matrix (Figure 7a–c). The eugenol-modified laponite platelets can be seen embedded within the LDPE film, resulting in relatively smooth surfaces. In the case of EG@Lap a rougher surface compared to laponite nanohybrid is present (Figure 7d–f). Overall, for both cases, as EG@clay content increases larger particles are observed implying particle agglomeration. We also note that the SEM images show a more compact morphology, indicating effective integration of the two types of clays and EG within the LDPE matrix. These features are advantageous for applications in packaging, where enhanced barrier properties and antimicrobial activity are desired.

### 3.3. Tensile Properties of LDPE/xEG@Lap and LDPE/xEG@Mt Active Films

**Figure 8:**
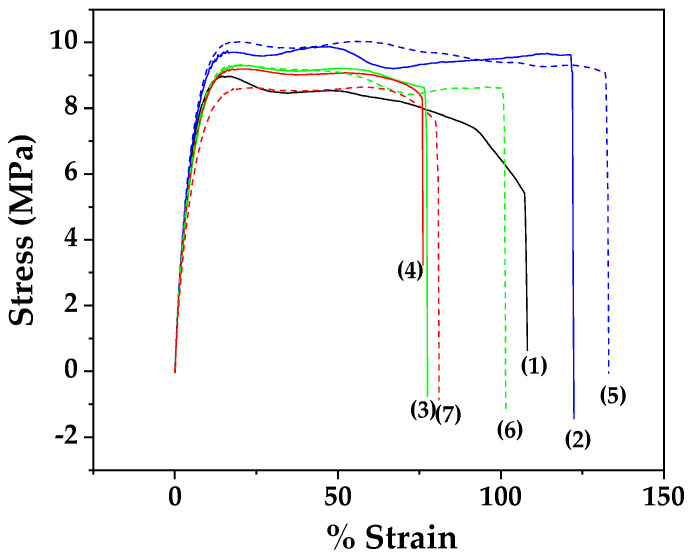
Stress-strain curves for (1) black line, LDPE, (2) blue line, LDPE/5EG@Lap, (3) green line, LDPE/10EG@Lap, (4) red line, LDPE/15EG@Lap, (5) dashed blue line, LDPE/5EG@Mt, (6) dashed green line, LDPE/10EG@Mt, and (7) dashed red line, LDPE/15EG@Mt.

Figure 8 shows the stress-strain curves for all LDPE/xEG@Lap and LDPE/xEG@Mt active films as well as for pure LDPE film.

From the stress-strain curves of Figure 8, the Elastic modulus E (MPa), ultimate strength σ_uts_ (MPa), and % elongation at break values were calculated and are listed in Table 3 for comparison.

Addition of EG@clay nanohybrids in LDPE leads to a softening of the films compared to the neat polymer. Interestingly, the softer films containing 5% clay led to increased ductility (higher % ε at break see Table 3). However, when the clay content is increased further, the elongation at break decreases probably due to the introduction of more defects with the more agglomerated clay particles. Consistent with the above, the ultimate strength of the 5%-containing clay also increased somewhat (perhaps not statistically significantly) compared to the neat polymer.

### 3.4. Water/Oxygen Barrier Properties of LDPE/xEG@Lap and LDPE/xEG@Mt Active Films

Table 4 summarizes the water vapor transmission rate (WVTR) and oxygen transmission rate (OTR) mean values as well as the calculated water vapor diffusion coefficient (D_wv_) and oxygen permeability Pe_O2_ mean values of all tested LDPE/xEG@Lap and LDPE/xEG@Mt active films as well as pure LDPE film.

It is obvious that both EG@Lap and EG@Mt nanohybrids enhance both the water and oxygen barrier properties compared to pure LDPE. The result agrees with previous reports that EO-modified halloysite, natural zeolite, activated carbon, and mesoporous silica SBA-15 dispersed in LDPE decreased the water and oxygen permeability [28,29,68,88]. The decrease in barrier properties is the result of the incorporation of the EG@Lap and EG@Mt nanohybrids [89,90,91]. Calculated D_wv_ values are not statistically changed between LDPE/xEG@Lap and LDPE/xEG@Mt active films while the lowest P_eO2_ values are obtained for LDPE films loaded with the lowest EG@Lap and EG@Mt nanohybrid content (LDPE/5EG@Lap and LDPE/5EG@Mt).

### 3.5. EG Release Kinetics from LDPE/xEG@Lap and LDPE/xEG@Mt Active Films

**Figure 9:**
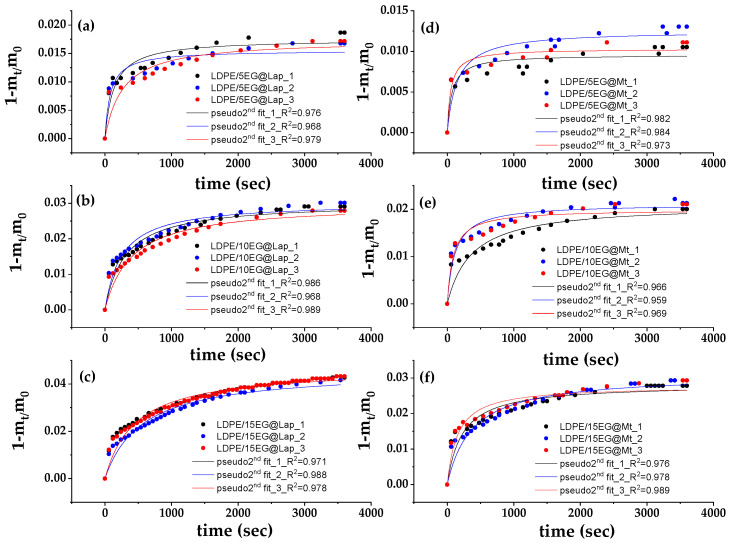
EG desorption isotherm kinetic plots (in triplicates) for all LDPE/xEG@Lap (**left** part (**a**–**c**) plots) and all LDPE/xEG@Mt (**right** part (**d**–**f**) plots) nanohybrids at 70 °C. The red line shows the simulation plots according to the pseudo-second-order kinetic model.

Figure 9 shows the (1 − m_t_/m_0_) values as a function of time (t) for all LDPE/xEG@Lap and all LDPE/xEG@Mt active films (in triplicate) at 70 °C. The data are fitted with the pseudo-second-order kinetic model to calculate the k_2_ and q_e_ values by using Equation (1). The resulting k_2_ and q_e_ mean values are listed in Table 5 for all LDPE/xEG@Lap and all LDPE/xEG@Mt active films.

As the EG@Lap and EG@Mt %wt. content increased, the k_2_ values decreased and q_e_ mean values increased for all LDPE/xEG@Lap and all LDPE/xEG@Mt active films. This means that as the EG@Lap and EG@Mt %wt. content increased the desorbed EG amount increased and the EG release rate decreased. This is because the dispersion of higher EG@Lap and EG@Mt amounts in the LDPE matrix decreases the diffusivity and mobility of EG molecules inside the LDPE matrix in accordance with previous reports [27,28,88]. Finally, all LDPE/xEG@Lap active films release higher amounts of EG with lower release rates than all LDPE/xEG@Mt active films. This is expected because of the adsorption of EG mostly on the external surfaces of Lap compared to Mt as shown above.

### 3.6. Antioxidant Activity of LDPE/xEG@Lap and LDPE/xEG@Mt Active Films

The EC_50,DPPH_ mean values for all LDPE/xEG@Lap and LDPE/xEG@Mt active films are listed in Table 5 for comparison. As the amount of EG@Lap and EG@Mt nanohybrids in LDPE active films increased, the EC_50,DPPH_ values decreased, which means that antioxidant activity increases. In addition, the LDPE/xEG@Lap active films achieved lower EC_50,DPPH_ than the LDPE/xEG@Mt active films suggesting higher antioxidant activity for the former. This result agrees with the higher release rates and higher % EG loaded amounts as described above for all LDPE/xEG@Lap compared to LDPE/xEG@Mt active films.

### 3.7. Packaging Preservation Test of Fresh Minced Pork Wrapped with LDPE/15EG@Lap and LDPE/15EG@Mt Active Films and Pure LDPE Film

#### 3.7.1. Total Viable Counts (TVCs)

Table 6 presents the results of TVCs in minced meat Samples coated with the examined films across the days of storage, allowing comparison of microbial growth between the different coatings.

The results underline that the LDPE/15EG@Lap and LDPE/15EG@Mt films are more effective in controlling microbial growth throughout the storage period compared to the other samples. Specifically, by Day 6, minced meat covered with LDPE film surpassed the 7 log CFU/g threshold, while the other two samples only reached this limit by Day 8, demonstrating the superior performance of both films. To conclude, the LDPE/15EG@Lap film achieved a 1log reduction in TVC compared to LDPE by Day 6, giving a shelf-life extension of two days more which is also a statistically significant result (see Supplementary Materials).

#### 3.7.2. Sensory Analysis Results

Table 7 presents the results of the impact of the tested films on the sensory attributes of minced meat, specifically aroma, color, and texture, throughout a 10-day storage period.

The results from the table above show that both LDPE/15EG@Lap and LDPE/15EG@Mt films exhibited the highest efficacy in preserving the aroma, color, and texture of minced meat throughout the storage period compared to LDPE. In terms of aroma and color, minced meat covered with LDPE exhibited a significant decline from Day 2 to Day 10. In contrast, both LDPE/15EG@Lap and LDPE/15EG@Mt films kept the meat’s sensory attributes stable, with no significant decline. Texture followed a similar trend, with LDPE showing significant deterioration between Day 2 and Day 10, while LDPE/15EG@Lap and LDPE/15EG@Mt remained more consistent over the storage period.

#### 3.7.3. pH Analysis

Table 8 lists the pH evolution of the coated minced meat by the three examined samples over 10 days.

As can be clearly seen from the results above, LDPE/15EG@Lap film seems to be the most effective in controlling pH over time without statistically significant fluctuations reaching 5.39. Moreover, minced meat coated with LDPE/15EG@Mt film shows high stability over the passage of storage days. In contrary LDPE film was observed exhibited a significant pH decline starting from Day 4, reaching the lowest value of 5.37 by Day 10.

#### 3.7.4. Lab* Colorimetry Analysis

Table 9 illustrates the changes in Lab* colorimetry parameters of coated mined meat over a 10 days storage period, underlining the impact of the coating on color stability throughout the examined period.

## 4. Discussion

While the adsorption of essential oils (EOs) and their derivatives onto nanoclays is well established, it is known that these compounds typically adhere to the external surfaces of nanoclays rather than intercalating into their interlayer spaces [26,36,92]. In non-intercalated EO@nanoclay hybrids, the interlayer space remains hydrophilic, making it incompatible with the hydrophobic chains of low-density polyethylene (LDPE), a commonly used packaging material. Achieving intercalation of EOs and their derivatives within the nanoclay interlayers could improve the controlled release properties of EO@nanoclay hybrids and enhance their functionality at higher loadings in polymer matrices like LDPE, thus enhancing the active packaging capabilities of these nanocomposite films [93].

In this study, we demonstrate for the first time successful intercalation of eugenol (EG) molecules into the interlayer spaces of montmorillonite (Mt) and laponite (Lap) nanoclays using a vacuum-assisted adsorption method, as confirmed by XRD, FTIR, and SEM analyses of the resulting EG@Mt and EG@Lap nanohybrids. The vacuum-assisted adsorption method also led to higher EG loading on Mt than previously reported. Desorption kinetics experiments showed that EG@Lap desorbs more EG and at a faster rate than EG@Mt, likely due to the greater adsorption of EG on the external surfaces of Lap.

The EG@Lap and EG@Mt nanohybrids were successfully incorporated into the LDPE matrix at low (5%wt.), medium (10%wt.), and high (15%wt.) concentrations via extrusion, resulting in LDPE/EG@Lap and LDPE/EG@Mt active packaging films. XRD analysis of the LDPE/xEG@Lap and LDPE/xEG@Mt films confirmed that there was no intercalation of LDPE chains into the nanohybrids, with both EG@Lap and EG@Mt dispersed uniformly throughout the LDPE matrix. FTIR analysis indicated effective integration of nanohybrids with LDPE chains without specific interactions, while SEM morphology studies revealed a compact structure that supports the effective integration of the nanoclays and EG, enhancing barrier and antimicrobial properties desirable for packaging applications.

The addition of EG@clay nanohybrids to LDPE resulted in a softening of the films compared to pure LDPE. However, as the clay content increased, elongation at break decreased, likely due to defect formation associated with clay particle agglomeration. Both EG@Lap and EG@Mt nanohybrids improved the water and oxygen barrier properties of LDPE, consistent with previous studies.

Increasing the EG@Lap and EG@Mt content in the films enhanced their antioxidant activity. Higher loadings also led to lower EG release rates (k_2_) and higher desorbed EG contents (q_e_) across all LDPE/xEG@Lap and LDPE/xEG@Mt films, likely due to reduced diffusivity and mobility of EG molecules within the LDPE matrix.

Overall, LDPE/15EG@Lap and LDPE/15EG@Mt active films showed mechanical strengths comparable to pure LDPE, but with reduced water and oxygen permeability, the highest antioxidant activity, and the most favorable release characteristics (highest q_e_ and lowest k_2_ values). Testing these optimized films as wrapping materials for fresh minced pork indicated that both LDPE/15EG@Lap and LDPE/15EG@Mt extended the meat’s shelf life, with LDPE/15EG@Lap providing an approximately two-day longer shelf-life extension than LDPE/15EG@Mt. This enhanced performance of LDPE/15EG@Lap is attributed to its higher EG content and faster EG release rate.

As shown in Table 1, the total EO released fraction per gram of nanohybrid (q_e_)) for EG@Lap is consistently higher than for EG@Mt (approximately 35% and 25%, respectively). This indicates a greater degree of intercalation and stronger bonding between the clay’s internal surface and the EO molecules in EG@Mt. Additionally, the k_2_ values indicate that below 70 °C, EG@Lap releases EO at a faster rate than EG@Mt likely due to differences in bonding. However, at higher temperatures (90 °C and 110 °C), EG@Mt shows an increased EO release rate, possibly due to enhanced diffusion. This trend is consistent with the desorption activation energy values (E_des_^0^) derived from the Arrhenius plots in Figure 3. The desorption energy for EG@Mt is 17.8 kcal/mol, which is higher than the 12.6 kcal/mol observed for EG@Lap, indicating that EG@Mt interactions more closely resemble chemisorption, while EG@Lap bonds are closer to physisorption. The activation energies fall within the 10–20 kcal/mol range, suggesting a combination of both physisorption and chemisorption mechanisms, supporting the pseudo-second-order kinetics assumption that these two phenomena coexist.

The diffusion coefficient values suggest that adding the EG nanohybrids enhances the water vapor barrier properties of the films while the oxygen permeability coefficient remains nearly constant, exhibiting a slight improvement. This effect may be due to the hydrophobic nature of EO. Comparing diffusion coefficients for LDPE/xEG@Lap and LDPE/xEG@Mt, it is evident that EG@Mt nanohybrids reduce these values more effectively, consistent with the lower EO release rate observed for the EG@Mt materials. Combined with the higher EO release (k_2_) of EG@Lap at temperatures below 70 °C, these findings align with the results of the minced meat storage experiments where the LDPE/15EG@Lap film demonstrated superior preservation performance.

## 5. Conclusions

It is well known that many of the recently developed novel biofilms could be applied as active food packaging means. Nevertheless, the transition from the old fashion to the new revolutionary techniques is a difficult enterprise for industries because of the financial resources needed to do this. This study is targeted to create an intermediate cheaper stage for this transition, combining the old with the new. It demonstrates the effectiveness of Lap and Mt as natural nanocarriers for EG and the incorporation of this bio-based nanohybrid in the commercially and widely used LDPE packaging films. This idea aims to improve shelf-life and quality preservation of perishable food items following the spirit of the circular economy and environmentally friendly methods. The research shows that both EG@Lap and EG@Mt nanohybrids enhance the barrier properties and antimicrobial effectiveness of LDPE films, though with distinct behaviors due to the different intercalation capacities and surface areas of Lap and Mt. The EG@Lap nanohybrids showed higher initial release rates and total desorbed eugenol quantities, attributed to Lap’s discoidal structure, which supports a more substantial eugenol loading and release profile compared to Mt.

In packaging trials with minced pork, the LDPE/15EG@Lap films extended shelf life by approximately two days longer than LDPE/15EG@Mt films. This finding highlights the potential of EG@Lap nanohybrids in active packaging applications where high release rates and enhanced food preservation are critical. Overall, this study underscores the advantages of using nanoclays, particularly laponite, for controlled release active packaging systems. Future studies could focus on scaling up production processes of such active packaging films and testing additional natural antimicrobials in combination with EG@Lap to further expand the applications of this active packaging material. Furthermore, future studies could focus on the addition of surfactants or surface modifiers to achieve the uniform distribution of nanoclays within the polymer matrix.

## Figures and Tables

**Table 1:** Sample names, contents of LDPE, EG@Lap, and the EG@Mt nanohybrid, and the twin-screw extruder operating conditions (temperature, rotation speed, and time) used for the development of all LDPE/xEG@Lap and LDPE/xEG@Mt active films.

Sample Name	LDPE (g)	EG@Lap (g-wt.%)	EG@Mt (g-wt.%)	Twin-Screw Extruder Operating Conditions		
				T (°C)	Speed (rpm)	Time (min)
LDPE	5	-	-	200	100	3
LDPE/5EG@Lap	4.75	0.25–5	-	200	100	3
LDPE/10EG@Lap	4.50	0.50–10	-	200	100	3
LDPE/15EG@Lap	4.25	0.75–15	-	200	100	3
LDPE/5EG@Mt	4.75	-	0.25–5	200	100	3
LDPE/10EG@Mt	4.50	-	0.50–10	200	100	3
LDPE/15EG@Mt	4.25	-	0.75–15	200	100	3

**Table 2:** Calculated k_2_, q_e_, and RR_EG,initial_ mean values from EG desorption kinetic plots for both EG@Lap and EG@Mt nanohybrids.

70 °C	EG@Lap	EG@Mt
**k_2_ (s^−1^)**	5.29 × 10^−4^ ± 0.24 × 10^−4^	3.34 × 10^−4^ ± 1.7 × 10^−4^
**q_e_**	0.338 ± 0.053	0.235 ± 0.031
**90 °C**	**EG@Lap**	**EG@Mt**
**k_2_ (s^−1^)**	1.07 × 10^−3^ ± 0.12 × 10^−3^	2.12 × 10^−3^ ± 0.31 × 10^−3^
**q_e_**	0.356 ± 0.013	0.264 ± 0.012
**110 °C**	**EG@Lap**	**EG@Mt**
**k_2_ (s^−1^)**	3.79 × 10^−3^ ± 0.8 × 10^−3^	5.27 × 10^−3^ ± 1.47 × 10^−3^
**q_e_**	0.365 ± 0.014	0.299 ± 0.025

**Table 3:** Elastic modulus E (MPa), ultimate strength σ_uts_ (MPa), and % elongation at break values were calculated for all LDPE/xEG@Lap, and LDPE/xEG@Mt active films as well as pure LDPE film.

Sample Name	E (Mpa)	σ_uts_ (MPa)	%ε
LDPE	206.8 ± 16.2 ^a^	9.5 ± 1.0 ^a,b^	106.5 ± 3.7 ^a,b^
LDPE/5EG@Lap	186.7 ± 13.9 ^a,b^	10.2 ± 0.4 ^a^	122.5 ± 4.6 ^a,d^
LDPE/10EG@Lap	162.9 ± 13.4 ^b,c^	9.5 ± 0.3 ^a,b^	76.2 ± 3.4 ^b,c^
LDPE/15EG@Lap	140.4 ± 3.2 ^c^	8.7 ± 0.2 ^b^	74.8 ± 3.0 ^c^
LDPE/5EG@Mt	182.8 ± 4.6 ^a,b^	10.0 ± 0.4 ^a^	132.6 ± 4.7 ^a^
LDPE/10EG@Mt	161.6 ± 3.1 ^b,c^	9.5 ± 0.4 ^a^.^b^	100.6 ± 4.5 ^a,c^
LDPE/15EG@Mt	159.5 ± 2.0 ^b,c^	9.4 ± 0.4 ^a,b^	85.0 ± 4.3 ^b,c,d^

^a,b,c,d^ Different letters in each column indicate statistically significant differences according to Kruskal–Wallis test at the confidence level *p* < 0.05.

**Table 4:** Water vapor transmission rate (WVTR) and oxygen transmission rate (OTR) mean values as well as the calculated water vapor diffusion coefficient (D_wv_) and oxygen permeability (Pe_O2_) mean values of all tested films.

	D_wv_ (cm^2^·s^−1^) × 10^−5^	P_eO2_ (cm^2^·s^−1^) × 10^−8^
LDPE	48.7 ± 9.5 ^a^	2.61 ± 0.013 ^a^
LDPE/5EG@Lap	9.3 ± 3.4 ^b^	1.77 ± 0.343 ^b^
LDPE/10EG@Lap	11.1 ± 6.8 ^b^	2.34 ± 0.082 ^a,c^
LDPE/15EG@Lap	11.7 ± 4.4 ^a,b^	2.03 ± 0.136 ^b,c,d^
LDPE/5EG@Mt	12.9 ± 6.2 ^a,b^	1.84 ± 0.026 ^b,d^
LDPE/10EG@Mt	8.8 ± 2.1 ^b^	2.08 ± 0.012 ^b,c^
LDPE/15EG@Mt	6.4 ± 4.1 ^b^	2.52 ± 0.265 ^a,c^

^a,b,c,d^ Different letters in each column indicate statistically significant differences according to Kruskal–Wallis test at the confidence level *p* < 0.05.

**Table 5:** Calculated k_2_, q_e_, and EC_50,DPPH_ mean values for all LDPE/xEG@Lap and LDPE/xEG@Mt active films.

	k_2_ (s^−1^)	q_e_	EC_50,DPPH_ (mg/L)
**LDPE**	-	-	n.d.
**LDPE/5EG@Lap**	0.480 ± 0.090	0.017 ± 0.001	12.1 ± 0.3
**LDPE/10EG@Lap**	0.110 ± 0.023	0.030 ± 0.002	0.33 ± 0.02
**LDPE/15EG@Lap**	0.040 ± 0.004	0.046 ± 0.003	0.11 ± 0.01
**LDPE/5EG@Mt**	1.280 ± 0.271	0.011 ± 0.002	18.14 ± 2.3
**LDPE/10EG@Mt**	0.370 ± 0.016	0.021 ± 0.001	3.57 ± 0.54
**LDPE/15EG@Mt**	0.160 ± 0.072	0.029 ± 0.001	1.29 ± 0.23

**Table 6:** Total viable counts (TVCs) in minced meat samples coated with the films over the examined storage period.

Samples	Day 0	Day 2	Day 4	Day 6	Day 8	Day 10
**LDPE**	4.68 ^Aa^	6.20 ^Bb^	6.44 ^Bc^	7.14 ^Cd^	8.13 ^De^	8.27 ^De^
**LDPE/15EG@Lap**	4.68 ^Aa^	5.49 ^Bb^	5.7 ^Bc^	6.08 ^Cd^	7.09 ^Ce^	7.78 ^Df^
**LDPE/15EG@Mt**	4.68 ^Aa^	6.09 ^Bb^	5.72 ^Bc^	6.30 ^Cd^	7.54 ^Ce^	8.09 ^Df^

^a,b,c,d,e,f^ Values with different small letters within the same row indicate statistically significant differences according to Kruskal–Wallis test at the 5% level (see Supplementary Materials). ^A,B,C,D^ Values with different capital letters within the same column indicate statistically significant differences according to Kruskal–Wallis test at the 5% level (See Supplementary Table S6).

**Table 7:** Sensory attribute changes in coated minced meat over 10 days of storage.

Parameters	Sample	Day 0	Day 2	Day 4	Day 6	Day 8	Day 10
	LDPE	5.00 ^Aa^	3.97 ^Bb^	3.40 ^Cc^	3.27 ^Cd^	2.77 ^De^	2.10 ^Ef^
**Aroma**	LDPE/15EG@Mt	5.00 ^Aa^	4.53 ^Bb^	3.87 ^Bc^	3.50 ^Cd^	3.30 ^Ce^	2.53 ^Df^
	LDPE/15EG@Lap	5.00 ^Aa^	4.47 ^Bb^	3.93 ^Bc^	3.40 ^Cd^	3.33 ^Ce^	2.73 ^Df^
**Color**	LDPE	5.00 ^Aa^	4.10 ^Bb^	4.10 ^Bb^	3.63 ^Cc^	3.53 ^Cd^	2.17 ^Df^
	LDPE/15EG@Mt	5.00 ^Aa^	4.37 ^Bb^	3.80 ^Bc^	3.43 ^Cd^	3.23 ^Ce^	2.43 ^Df^
	LDPE/15EG@Lap	5.00 ^Aa^	4.57 ^Bb^	3.90 ^Bc^	3.43 ^Cd^	3.27 ^Ce^	2.57 ^Df^
**Texture**	LDPE	5.00 ^Aa^	4.03 ^Bb^	3.73 ^Cc^	3.80 ^Bd^	3.67 ^Be^	2.57 ^Cf^
	LDPE/15EG@Mt	5.00 ^Aa^	4.57 ^Bb^	3.87 ^Bc^	3.63 ^Bd^	3.57 ^Be^	2.73 ^Cf^
	LDPE/15EG@Lap	5.00 ^Aa^	4.57 ^Bb^	3.93 ^Bc^	3.87 ^Bc^	3.77 ^Bd^	2.87 ^Ce^

^a,b,c,d,e,f^ Values with different small letters within the same row indicate statistically significant differences according to Kruskal–Wallis test at the 5% level (see Supplementary Materials). ^A,B,C,D,E^ Values with different capital letters within the same column indicate statistically significant differences according to Kruskal–Wallis test at the 5% level (see Supplementary Materials).

**Table 8:** pH changes in minced meat samples over 10 days.

Samples	Day 0	Day 2	Day 4	Day 6	Day 8	Day 10
LDPE	5.66 (±0.04) ^Aa^	5.57 (±0.04) ^Aa^	5.47 (±0.04) ^Bb^	5.50 (±0.04) ^Ab^	5.46 (±0.04) ^Ab^	5.37 (±0.04) ^Ac^
LDPE/15EG@Mt	5.66 (±0.04) ^Aa^	5.53 (±0.04) ^Aa^	5.54 (±0.04) ^Ab^	5.58 (±0.04) ^Bc^	5.52 (±0.04) ^Ac^	5.44 (±0.04) ^Bd^
LDPE/15EG@Lap	5.66 (±0.04) ^Aa^	5.53 (±0.04) ^Aa^	5.52 (±0.04) ^Ab^	5.55 (±0.04) ^Ab^	5.52 (±0.04) ^Bc^	5.39 (±0.04) ^Ad^

^a,b,c,d^ Values with different small letters within the same row indicate statistically significant differences according to Kruskal–Wallis test at the 5% level (see Supplementary Materials). ^A,B^ Values with different capital letters within the same column indicate statistically significant differences according to Kruskal–Wallis test at the 5% level (see Supplementary Materials).

**Table 9:** Variation in Lab* colorimetry parameters of coated minced meat during 10 days of storage.

Sample	Days	L*	a*	b*	ΔL (±std)	Δa (±std)	Δb (±std)	ΔE (±std)
LDPE	Day 0	56.27 (±0.52) ^Aa^	16.96 (±0.64) ^Aa^	10.37 (±0.19) ^Aa^	—	—	—	—
LDPE/15EG@Mt	Day 0	56.27 (±0.52) ^Aa^	16.96 (±0.64) ^Aa^	10.37 (±0.19) ^Aa^	—	—	—	—
LDPE/15EG@Lap	Day 0	56.27 (±0.52) ^Aa^	16.96 (±0.64) ^Aa^	10.37 (±0.19) ^Aa^	—	—	—	—
LDPE	Day 2	53.34 (±0.38) ^Ba^	12.49 (±0.53) ^Bb^	12.19 (±0.86) ^Ba^	−2.93 (±0.38)	−4.47 (±0.53)	1.82 (±0.86)	5.65 (±0.38)
LDPE/15EG@Mt	Day 2	55.34 (±0.39) ^Aa^	16.33 (±0.35) ^Aa^	10.53 (±0.45) ^Aa^	−0.93 (±0.39)	−0.63 (±0.35)	0.16 (±0.45)	1.13 (±0.39)
LDPE/15EG@Lap	Day 2	56.09 (±0.86) ^Aa^	16.06 (±0.26) ^Aa^	10.41 (±0.36) ^Aa^	−0.18 (±0.86)	−0.9 (±0.26)	0.04 (±0.36)	0.92 (±0.86)
LDPE	Day 4	50.75 (±0.47) ^Ca^	10.24 (±0.68) ^Cb^	12.92 (±0.91) ^Bb^	−2.59 (±0.47)	−2.25 (±0.68)	0.73 (±0.91)	3.51 (±0.47)
LDPE/15EG@Mt	Day 4	53.18 (±0.54) ^Ba^	14.19 (±0.76) ^Ba^	12.03 (±0.41) ^Ab^	−2.16 (±0.54)	−2.14 (±0.76)	1.5 (±0.41)	3.39 (±0.54)
LDPE/15EG@Lap	Day 4	53.00 (±0.53) ^Ba^	14.75 (±0.48) ^Bb^	11.86 (±0.45) ^Ab^	−3.09 (±0.53)	−1.31 (±0.48)	1.45 (±0.45)	3.66 (±0.53)
LDPE	Day 6	47.23 (±0.46) ^Db^	13.35 (±0.59) ^Db^	13.79 (±1.04) ^Bb^	−3.52 (±0.46)	3.11 (±0.59)	0.87 (±1.04)	4.78 (±0.46)
LDPE/15EG@Mt	Day 6	51.05 (±0.52) ^Ca^	13.62 (±0.73) ^Ca^	12.51 (±0.42) ^Ab^	−2.13 (±0.52)	−0.57 (±0.73)	0.48 (±0.42)	2.26 (±0.52)
LDPE/15EG@Lap	Day 6	50.35 (±0.50) ^Ca^	13.87 (±0.45) ^Cb^	12.57 (±0.48) ^Ab^	−2.65 (±0.50)	−0.88 (±0.45)	0.71 (±0.48)	2.88 (±0.50)
LDPE	Day 8	41.06 (±0.38) ^Ec^	11.48 (±0.61) ^Ec^	15.62 (±1.10) ^Cc^	−6.17 (±0.38)	−1.87 (±0.61)	1.83 (±1.10)	6.7 (±0.38)
LDPE/15EG@Mt	Day 8	49.01 (±0.50) ^Cb^	13.08 (±0.70) ^Ca^	13.01 (±0.44) ^Ab^	−2.04 (±0.50)	−0.54 (±0.70)	0.5 (±0.44)	2.17 (±0.50)
LDPE/15EG@Lap	Day 8	47.83 (±0.47) ^Db^	13.03 (±0.43) ^Cb^	13.33 (±0.51) ^Ab^	−2.52 (±0.47)	−0.84 (±0.43)	0.76 (±0.51)	2.76 (±0.47)
LDPE	Day 10	35.72 (±0.33) ^Fd^	9.99 (±0.53) ^Fd^	17.65 (±1.24) ^Cd^	−5.34 (±0.33)	−1.49 (±0.53)	2.03 (±1.24)	5.9 (±0.33)
LDPE/15EG@Mt	Day 10	47.05 (±0.48) ^Cb^	12.55 (±0.67) ^Ca^	13.53 (±0.46) ^Ab^	−1.96 (±0.48)	−0.53 (±0.67)	0.52 (±0.46)	2.1 (±0.48)
LDPE/15EG@Lap	Day 10	45.44 (±0.45) ^Db^	12.25 (±0.40) ^Cb^	14.13 (±0.54) ^Bd^	−2.39 (±0.45)	−0.78 (±0.40)	0.8 (±0.54)	2.64 (±0.45)

^a,b,c,d^ Values with different small letters within the same row indicate statistically significant differences according to Kruskal–Wallis test at the 5% level (see Supplementary Materials). ^A,B,C,D,E,F^ Values with different capital letters within the same column indicate statistically significant differences according to Kruskal–Wallis test at the 5% level (see Supplementary Materials).

## Data Availability

The datasets generated for this study are available on request to the corresponding author.

## Supplementary Materials

The following supporting information can be downloaded at: https://www.mdpi.com/article/10.3390/nano14231938/s1.
