# Supplementary material for: Laponite vs. Montmorillonite as Eugenol Nanocarriers for Low Density Polyethylene Active Packaging Films
Source: Nanomaterials (Basel). 2024 Dec 2;14(23):1938. doi: 10.3390/nano14231938 (PMC11643650; doi:10.3390/nano14231938)
Supplement: Supplementary file 1 [file nanomaterials-14-01938-s001.zip › nanomaterials-3324036-supplementary.pdf]

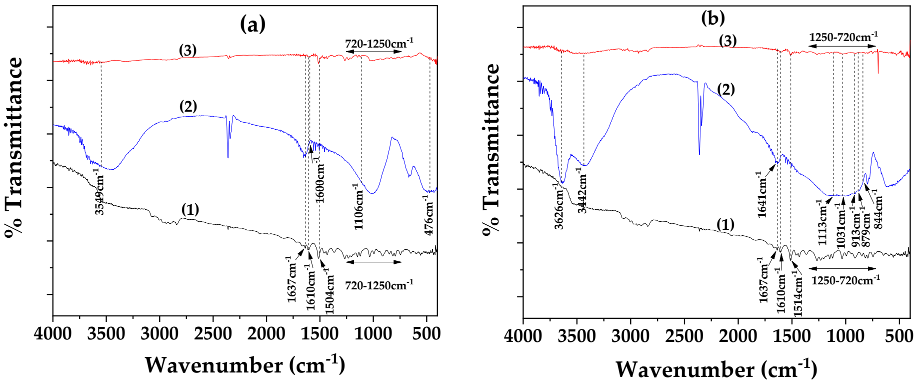

Figure S1. (a) FTIR plots of (1) pure EG, (2) pure Lap and (3) EG@Lap nanohybrid, **(b)** FTIR plots of (1) pure EG, (2) pure Mt and (3) EG@Mt nanohybrid.

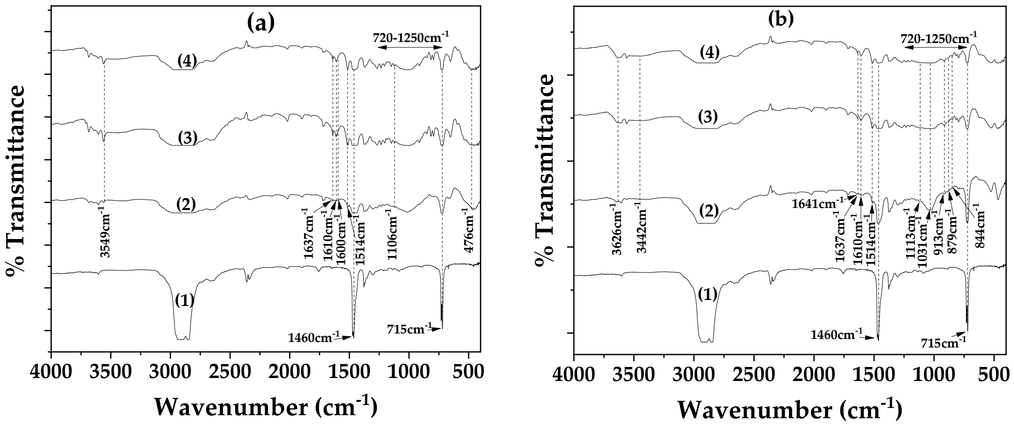

Figure S2. (a) FTIR plots of (1) pure LDPE film, (2) LDPE/5EG@Lap active film, (3) LDPE/10EG@Lap active film, and (4) LDPE/15EG@Lap active film **(b)** FTIR plots of (1) pure LDPE film, (2) LDPE/5EG@Mt active film, (3) LDPE/10EG@Mt active film, and (4) LDPE/15EG@Mt active film

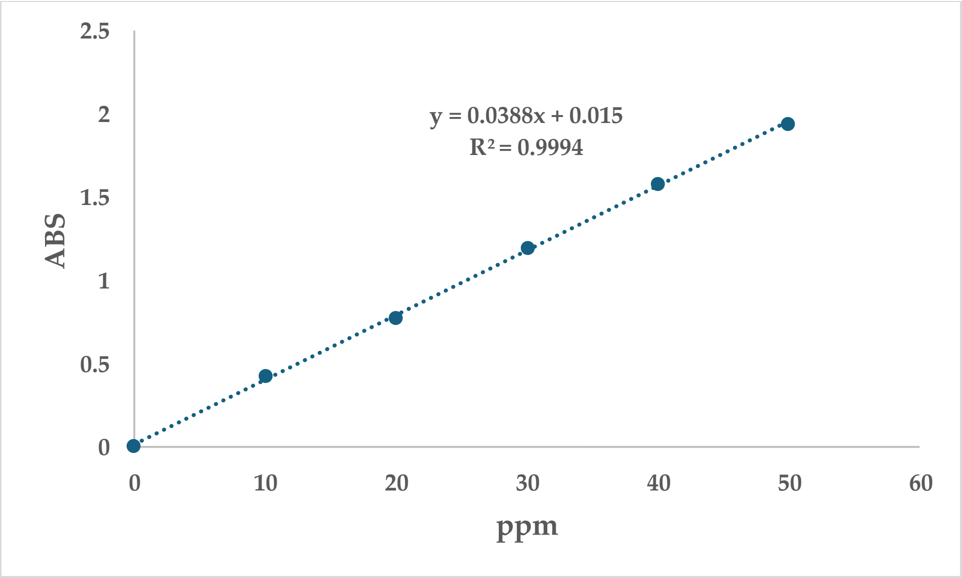

Figure S3. Calibration curve used for the EC50 experiments

STATISTICAL ANALYSIS FOR TENSILE PROPERTIES

Explore

Notes

Output Created		18-OCT-2024 09:29:14
Comments		
Input	Data	G:\To Drive μου\1. PAPERS\64. Cornell Eugenol Nanomaterials\STATISTICS \tensile.sav
	Active Dataset	DataSet1
	Filter	<none>
	Weight	<none>
	Split File	<none>
	N of Rows in Working Data File	21
Missing Value Handling	Definition of Missing	User-defined missing values for dependent variables are treated as missing.
	Cases Used	Statistics are based on cases with no missing values for

		any dependent variable or factor used.
Syntax		EXAMINE VARIABLES=E_Young_Mod σuts ε_elongation BY sample /PLOT NONE /STATISTICS DESCRIPTIVES /CINTERVAL 95 /MISSING LISTWISE /NOTOTAL.
Resources	Processor Time	00:00:00.02
	Elapsed Time	00:00:00.05

[DataSet1] G:\To Drive μου\1. PAPERS\64. Cornell Eugenol Nanomaterials\STATISTICS\tensile.sav

sample

Case Processing Summary

		Cases					
		Valid		Missing		Total	
	sample	N	Percent	N	Percent	N	Percent
E_Young_Mod	LDPE	3	100,0%	0	0,0%	3	100,0%
	LDPE_5EG_Lap	3	100,0%	0	0,0%	3	100,0%
	LDPE_10EG_Lap	3	100,0%	0	0,0%	3	100,0%
	LDPE_15EG_Lap	3	100,0%	0	0,0%	3	100,0%
	LDPE_5EG_Mt	3	100,0%	0	0,0%	3	100,0%
	LDPE_10EG_Mt	3	100,0%	0	0,0%	3	100,0%
	LDPE_15EG_Mt	3	100,0%	0	0,0%	3	100,0%
σuts	LDPE	3	100,0%	0	0,0%	3	100,0%
	LDPE_5EG_Lap	3	100,0%	0	0,0%	3	100,0%
	LDPE_10EG_Lap	3	100,0%	0	0,0%	3	100,0%
	LDPE_15EG_Lap	3	100,0%	0	0,0%	3	100,0%
	LDPE_5EG_Mt	3	100,0%	0	0,0%	3	100,0%
	LDPE_10EG_Mt	3	100,0%	0	0,0%	3	100,0%

	LDPE_15EG_Mt	3	100,0%	0	0,0%	3	100,0%
ε_elongation	LDPE	3	100,0%	0	0,0%	3	100,0%
	LDPE_5EG_Lap	3	100,0%	0	0,0%	3	100,0%
	LDPE_10EG_Lap	3	100,0%	0	0,0%	3	100,0%
	LDPE_15EG_Lap	3	100,0%	0	0,0%	3	100,0%
	LDPE_5EG_Mt	3	100,0%	0	0,0%	3	100,0%
	LDPE_10EG_Mt	3	100,0%	0	0,0%	3	100,0%
	LDPE_15EG_Mt	3	100,0%	0	0,0%	3	100,0%

Descriptives

	sample			Statistic	Std. Error
E_Young_Mod	LDPE	Mean		206,808333	9,3401758
		95% Confidence Interval for Mean	Lower Bound	166,620800	
			Upper Bound	246,995866	
		5% Trimmed Mean		.	
		Median		201,475000	
		Variance		261,717	
		Std. Deviation		16,1776590	
		Minimum		193,9707	
		Maximum		224,9793	
		Range		31,0086	
		Interquartile Range		.	
		Skewness		1,322	1,225
		Kurtosis		.	.
	LDPE_5EG_Lap	Mean		186,733000	8,0393138
		95% Confidence Interval for Mean	Lower Bound	152,142624	
			Upper Bound	221,323376	
		5% Trimmed Mean		.	
		Median		186,733000	
		Variance		193,892	
		Std. Deviation		13,9245000	
		Minimum		172,8085	

		Maximum		200,6575	
		Range		27,8490	
		Interquartile Range		.	
		Skewness		,000	1,225
		Kurtosis		.	.
	LDPE_10EG_Lap	Mean		162,939000	7,7606895
		95% Confidence Interval for Mean	Lower Bound	129,547448	
			Upper Bound	196,330552	
		5% Trimmed Mean		.	
		Median		163,060000	
		Variance		180,685	
		Std. Deviation		13,4419085	
		Minimum		149,4370	
		Maximum		176,3200	
		Range		26,8830	
		Interquartile Range		.	
		Skewness		-,041	1,225
		Kurtosis		.	.
	LDPE_15EG_Lap	Mean		140,410000	1,8391937
		95% Confidence Interval for Mean	Lower Bound	132,496588	
			Upper Bound	148,323412	
		5% Trimmed Mean		.	
		Median		139,790000	
		Variance		10,148	
		Std. Deviation		3,1855769	
		Minimum		137,5800	
		Maximum		143,8600	
		Range		6,2800	
		Interquartile Range		.	
		Skewness		,843	1,225

		Kurtosis		.	.
	LDPE_5EG_Mt	Mean		182,829333	2,6286182
		95% Confidence Interval for Mean	Lower Bound	171,519302	
			Upper Bound	194,139365	
		5% Trimmed Mean		.	
		Median		182,540000	
		Variance		20,729	
		Std. Deviation		4,5529003	
		Minimum		178,4280	
		Maximum		187,5200	
		Range		9,0920	
		Interquartile Range		.	
		Skewness		,285	1,225
		Kurtosis		.	.
	LDPE_10EG_Mt	Mean		161,556667	1,7881493
		95% Confidence Interval for Mean	Lower Bound	153,862881	
			Upper Bound	169,250452	
		5% Trimmed Mean		.	
		Median		162,350000	
		Variance		9,592	
		Std. Deviation		3,0971654	
		Minimum		158,1400	
		Maximum		164,1800	
		Range		6,0400	
		Interquartile Range		.	
		Skewness		-1,077	1,225
		Kurtosis		.	.
	LDPE_15EG_Mt	Mean		159,450000	1,1401316
		95% Confidence Interval for Mean	Lower Bound	154,544410	
			Upper Bound	164,355590	

		5% Trimmed Mean		.	
		Median		159,810000	
		Variance		3,900	
		Std. Deviation		1,9747658	
		Minimum		157,3200	
		Maximum		161,2200	
		Range		3,9000	
		Interquartile Range		.	
		Skewness		-,793	1,225
		Kurtosis		.	.
σuts	LDPE	Mean		9,533100	,5581000
		95% Confidence Interval for Mean	Lower Bound	7,131790	
			Upper Bound	11,934410	
		5% Trimmed Mean		.	
		Median		8,975000	
		Variance		,934	
		Std. Deviation		,9666576	
		Minimum		8,9750	
		Maximum		10,6493	
		Range		1,6743	
		Interquartile Range		.	
		Skewness		1,732	1,225
		Kurtosis		.	.
	LDPE_5EG_Lap	Mean		10,183600	,2136000
		95% Confidence Interval for Mean	Lower Bound	9,264553	
			Upper Bound	11,102647	
		5% Trimmed Mean		.	
		Median		9,970000	
		Variance		,137	
		Std. Deviation		,3699661	

		Minimum		9,9700	
		Maximum		10,6108	
		Range		,6408	
		Interquartile Range		.	
		Skewness		1,732	1,225
		Kurtosis		.	.
	LDPE_10EG_Lap	Mean		9,463700	,1777000
		95% Confidence Interval for Mean	Lower Bound	8,699119	
			Upper Bound	10,228281	
		5% Trimmed Mean		.	
		Median		9,286000	
		Variance		,095	
		Std. Deviation		,3077854	
		Minimum		9,2860	
		Maximum		9,8191	
		Range		,5331	
		Interquartile Range		.	
		Skewness		1,732	1,225
		Kurtosis		.	.
	LDPE_15EG_Lap	Mean		8,702133	,1101333
		95% Confidence Interval for Mean	Lower Bound	8,228268	
			Upper Bound	9,175999	
		5% Trimmed Mean		.	
		Median		8,592000	
		Variance		,036	
		Std. Deviation		,1907565	
		Minimum		8,5920	
		Maximum		8,9224	
		Range		,3304	
		Interquartile Range		.	

		Skewness		1,732	1,225
		Kurtosis		.	.
	LDPE_5EG_Mt	Mean		9,967200	,2582000
		95% Confidence Interval for Mean	Lower Bound	8,856255	
			Upper Bound	11,078145	
		5% Trimmed Mean		.	
		Median		9,709000	
		Variance		,200	
		Std. Deviation		,4472155	
		Minimum		9,7090	
		Maximum		10,4836	
		Range		,7746	
		Interquartile Range		.	
		Skewness		1,732	1,225
		Kurtosis		.	.
	LDPE_10EG_Mt	Mean		9,490500	,2045000
		95% Confidence Interval for Mean	Lower Bound	8,610608	
			Upper Bound	10,370392	
		5% Trimmed Mean		.	
		Median		9,286000	
		Variance		,125	
		Std. Deviation		,3542044	
		Minimum		9,2860	
		Maximum		9,8995	
		Range		,6135	
		Interquartile Range		.	
		Skewness		1,732	1,225
		Kurtosis		.	.
	LDPE_15EG_Mt	Mean		9,379167	,2111667
		95% Confidence Interval for Mean	Lower Bound	8,470590	
			Upper Bound	10,287744	

		5% Trimmed Mean		.	
		Median		9,168000	
		Variance		,134	
		Std. Deviation		,3657514	
		Minimum		9,1680	
		Maximum		9,8015	
		Range		,6335	
		Interquartile Range		.	
		Skewness		1,732	1,225
		Kurtosis		.	.
ε_elongation	LDPE	Mean		106,533333	2,1419098
		95% Confidence Interval for Mean	Lower Bound	97,317439	
			Upper Bound	115,749228	
		5% Trimmed Mean		.	
		Median		107,300000	
		Variance		13,763	
		Std. Deviation		3,7098967	
		Minimum		102,5000	
		Maximum		109,8000	
		Range		7,3000	
		Interquartile Range		.	
		Skewness		-,890	1,225
		Kurtosis		.	.
	LDPE_5EG_Lap	Mean		122,533333	2,6459613
		95% Confidence Interval for Mean	Lower Bound	111,148681	
			Upper Bound	133,917986	
		5% Trimmed Mean		.	
		Median		121,900000	
		Variance		21,003	
		Std. Deviation		4,5829394	

		Minimum		118,3000	
		Maximum		127,4000	
		Range		9,1000	
		Interquartile Range		.	
		Skewness		,610	1,225
		Kurtosis		.	.
	LDPE_10EG_Lap	Mean		76,166667	1,9810211
		95% Confidence Interval for Mean	Lower Bound	67,643021	
			Upper Bound	84,690312	
		5% Trimmed Mean		.	
		Median		76,700000	
		Variance		11,773	
		Std. Deviation		3,4312291	
		Minimum		72,5000	
		Maximum		79,3000	
		Range		6,8000	
		Interquartile Range		.	
		Skewness		-,683	1,225
		Kurtosis		.	.
	LDPE_15EG_Lap	Mean		74,800000	1,7473790
		95% Confidence Interval for Mean	Lower Bound	67,281635	
			Upper Bound	82,318365	
		5% Trimmed Mean		.	
		Median		75,800000	
		Variance		9,160	
		Std. Deviation		3,0265492	
		Minimum		71,4000	
		Maximum		77,2000	
		Range		5,8000	
		Interquartile Range		.	

		Skewness		-1,325	1,225
		Kurtosis		.	.
	LDPE_5EG_Mt	Mean		132,566667	2,7339430
		95% Confidence Interval for Mean	Lower Bound	120,803459	
			Upper Bound	144,329874	
		5% Trimmed Mean		.	
		Median		131,900000	
		Variance		22,423	
		Std. Deviation		4,7353282	
		Minimum		128,2000	
		Maximum		137,6000	
		Range		9,4000	
		Interquartile Range		.	
		Skewness		,621	1,225
		Kurtosis		.	.
	LDPE_10EG_Mt	Mean		100,633333	2,5718562
		95% Confidence Interval for Mean	Lower Bound	89,567529	
			Upper Bound	111,699138	
		5% Trimmed Mean		.	
		Median		100,400000	
		Variance		19,843	
		Std. Deviation		4,4545857	
		Minimum		96,3000	
		Maximum		105,2000	
		Range		8,9000	
		Interquartile Range		.	
		Skewness		,235	1,225
		Kurtosis		.	.
	LDPE_15EG_Mt	Mean		85,013333	2,4616074
		95% Confidence Interval for Mean	Lower Bound	74,421891	
			Upper Bound	95,604775	

	5% Trimmed Mean	.	
	Median	87,200000	
	Variance	18,179	
	Std. Deviation	4,2636291	
	Minimum	80,1000	
	Maximum	87,7400	
	Range	7,6400	
	Interquartile Range	.	
	Skewness	-1,701	1,225
	Kurtosis	.	.

Nonparametric Tests

Notes

Output Created		18-OCT-2024 09:29:46
Comments		
Input	Data	G:\To Drive μου\1. PAPERS\64. Cornell Eugenol Nanomaterials\STATISTICS \tensile.sav
	Active Dataset	DataSet1
	Filter	<none>
	Weight	<none>
	Split File	<none>
	N of Rows in Working Data File	21
Syntax		NPTESTS /INDEPENDENT TEST (E_Young_Mod σuts ε_elongation) GROUP (sample) KRUSKAL_WALLIS(COMPARE=PAIRWISE) /MISSING SCOPE=ANALYSIS

		USERMISSING=EXCLUDE /CRITERIA ALPHA=0.05 CILEVEL=95.
Resources	Processor Time	00:00:03.77
	Elapsed Time	00:00:03.10

Hypothesis Test Summary

	Null Hypothesis	Test	Sig.[a,b]
1	The distribution of E_Young_Mod is the same across categories of sample.	Independent-Samples Kruskal-Wallis Test	,008
2	The distribution of σuts is the same across categories of sample.	Independent-Samples Kruskal-Wallis Test	,060
3	The distribution of ε_elongation is the same across categories of sample.	Independent-Samples Kruskal-Wallis Test	,004

Hypothesis Test Summary

	Decision
1	Reject the null hypothesis.
2	Retain the null hypothesis.
3	Reject the null hypothesis.

a. The significance level is ,050.

b. Asymptotic significance is displayed.

Independent-Samples Kruskal-Wallis Test

E_Young_Mod across sample

Independent-Samples Kruskal-Wallis Test Summary

Total N	21
Test Statistic	17,524[a]
Degree Of Freedom	6
Asymptotic Sig.(2-sided test)	,008

a. The test statistic is adjusted for ties.

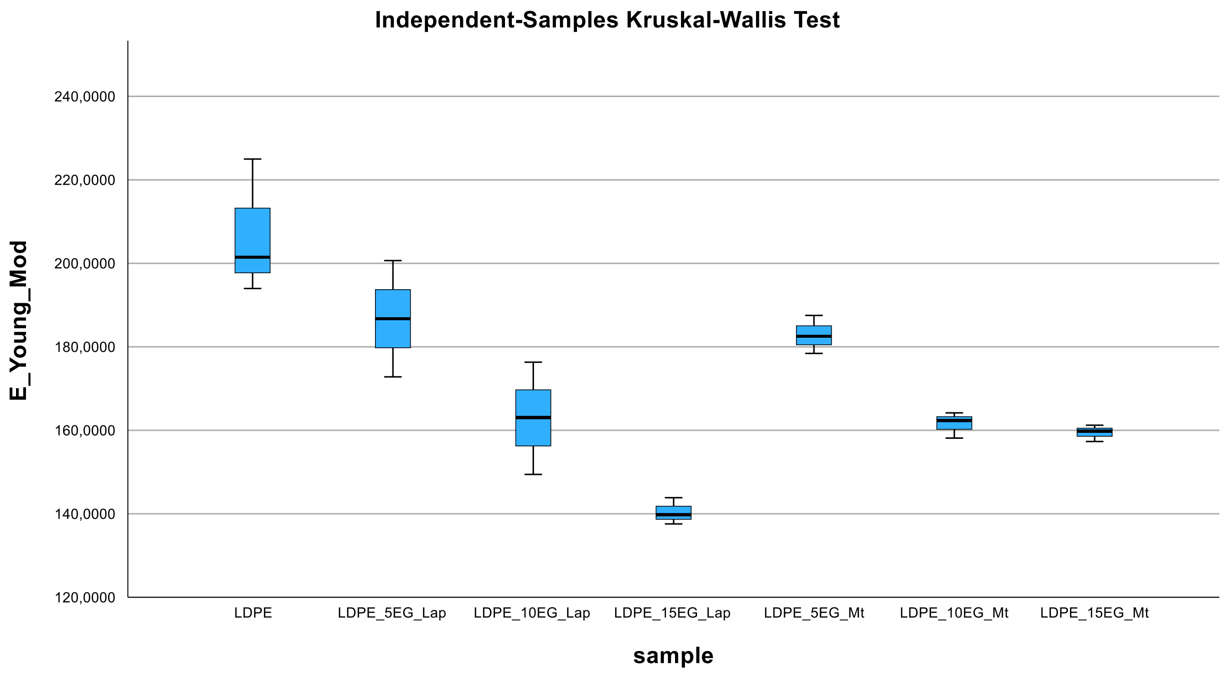

Pairwise Comparisons of sample

Sample 1-Sample 2	Test Statistic	Std. Error	Std. Test Statistic	Sig.	Adj. Sig.[a]
LDPE_15EG_Lap-LDPE_15EG_Mt	-4,667	5,066	-,921	,357	1,000
LDPE_15EG_Lap-LDPE_10EG_Mt	-6,667	5,066	-1,316	,188	1,000
LDPE_15EG_Lap-LDPE_10EG_Lap	7,000	5,066	1,382	,167	1,000
LDPE_15EG_Lap-LDPE_5EG_Mt	-13,333	5,066	-2,632	,008	,178
LDPE_15EG_Lap-LDPE_5EG_Lap	13,667	5,066	2,698	,007	,147
LDPE_15EG_Lap-LDPE	17,667	5,066	3,487	<,001	,010
LDPE_15EG_Mt-LDPE_10EG_Mt	2,000	5,066	,395	,693	1,000

LDPE_15EG_Mt-LDPE_10EG_Lap	2,333	5,066	,461	,645	1,000
LDPE_15EG_Mt-LDPE_5EG_Mt	8,667	5,066	1,711	,087	1,000
LDPE_15EG_Mt-LDPE_5EG_Lap	9,000	5,066	1,776	,076	1,000
LDPE_15EG_Mt-LDPE	13,000	5,066	2,566	,010	,216
LDPE_10EG_Mt-LDPE_10EG_Lap	,333	5,066	,066	,948	1,000
LDPE_10EG_Mt-LDPE_5EG_Mt	6,667	5,066	1,316	,188	1,000
LDPE_10EG_Mt-LDPE_5EG_Lap	7,000	5,066	1,382	,167	1,000
LDPE_10EG_Mt-LDPE	11,000	5,066	2,171	,030	,628
LDPE_10EG_Lap-LDPE_5EG_Mt	-6,333	5,066	-1,250	,211	1,000
LDPE_10EG_Lap-LDPE_5EG_Lap	6,667	5,066	1,316	,188	1,000
LDPE_10EG_Lap-LDPE	10,667	5,066	2,105	,035	,740
LDPE_5EG_Mt-LDPE_5EG_Lap	,333	5,066	,066	,948	1,000
LDPE_5EG_Mt-LDPE	4,333	5,066	,855	,392	1,000
LDPE_5EG_Lap-LDPE	4,000	5,066	,790	,430	1,000

Each row tests the null hypothesis that the Sample 1 and Sample 2 distributions are the same.
Asymptotic significances (2-sided tests) are displayed. The significance level is ,050.

a. Significance values have been adjusted by the Bonferroni correction for multiple tests.

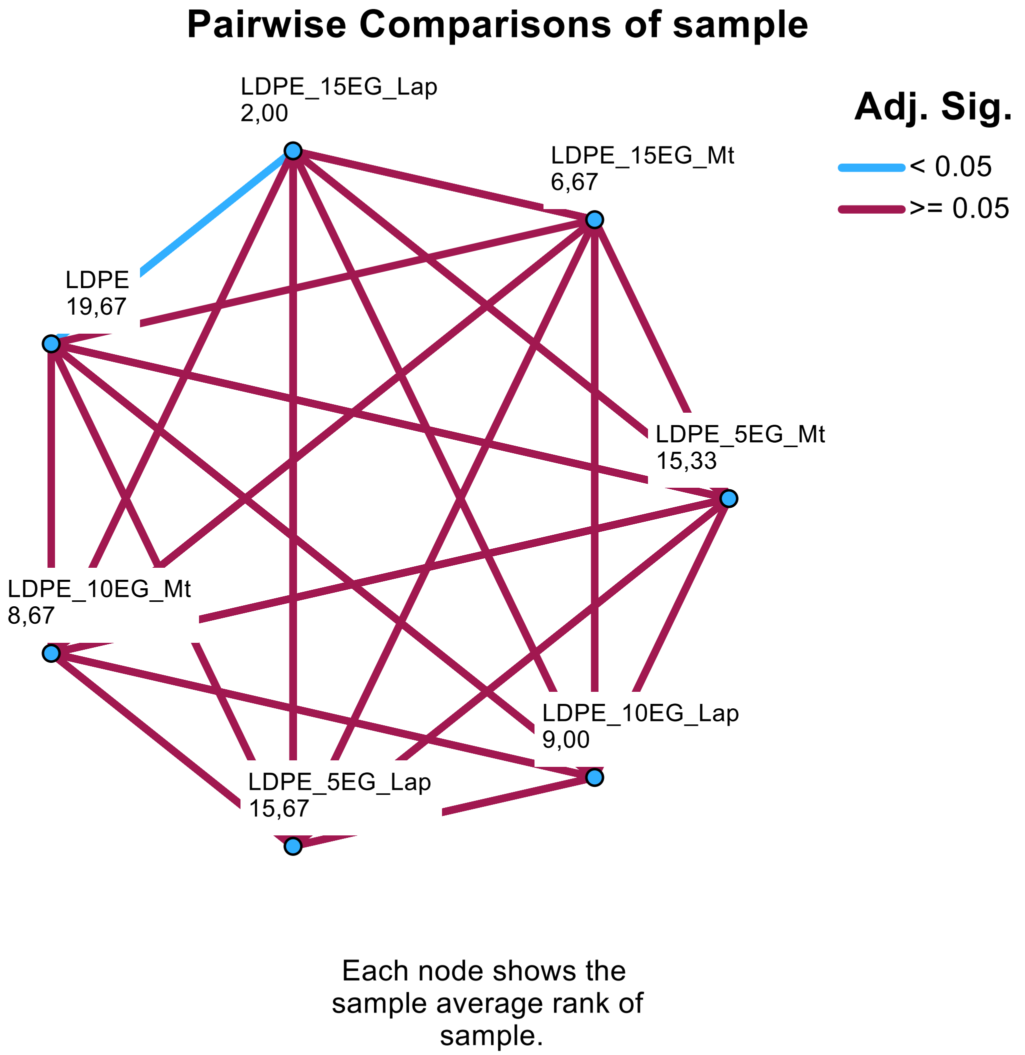

σuts across sample

Independent-Samples Kruskal-Wallis Test Summary

Total N	21
Test Statistic	12,101[a]
Degree Of Freedom	6

Asymptotic Sig.(2-sided test)	,060

a. The test statistic is adjusted for ties.

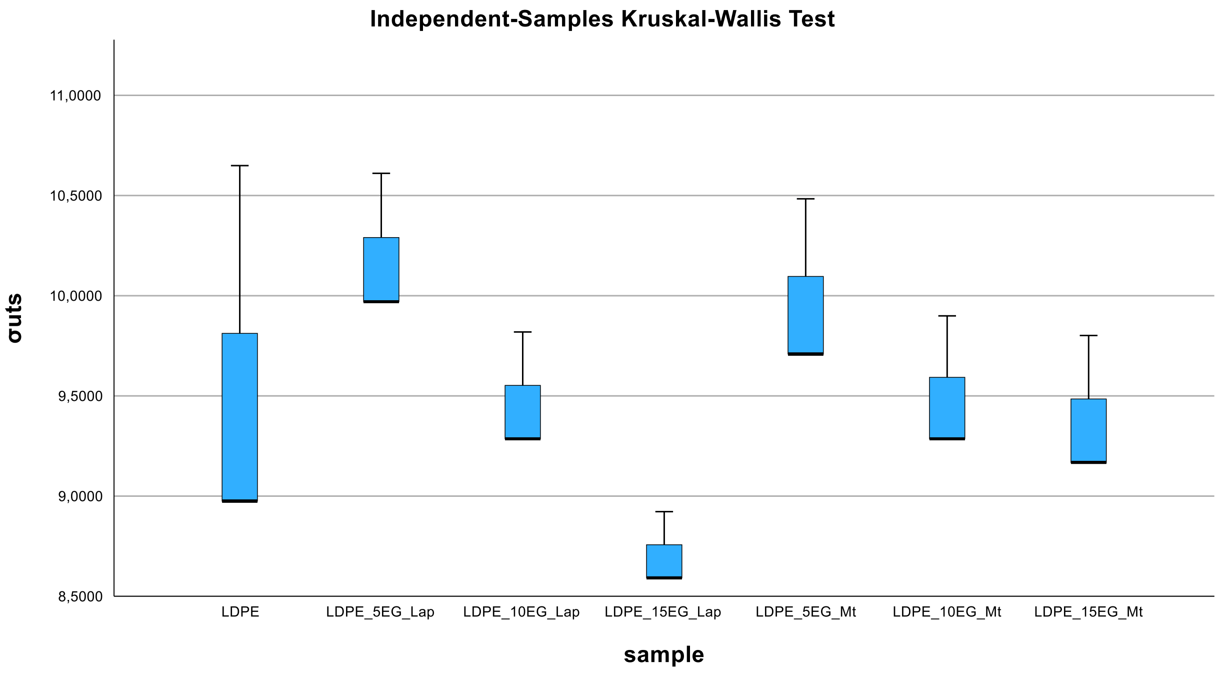

Pairwise Comparisons of sample

Sample 1-Sample 2	Test Statistic	Std. Error	Std. Test Statistic	Sig.	Adj. Sig.[a]
LDPE_15EG_Lap-LDPE_15EG_Mt	-7,000	5,041	-1,388	,165	1,000
LDPE_15EG_Lap-LDPE	8,000	5,041	1,587	,113	1,000
LDPE_15EG_Lap-LDPE_10EG_Lap	9,333	5,041	1,851	,064	1,000
LDPE_15EG_Lap-LDPE_10EG_Mt	-9,667	5,041	-1,917	,055	1,000
LDPE_15EG_Lap-LDPE_5EG_Mt	-12,667	5,041	-2,512	,012	,252
LDPE_15EG_Lap-LDPE_5EG_Lap	16,333	5,041	3,240	,001	,025
LDPE_15EG_Mt-LDPE	1,000	5,041	,198	,843	1,000
LDPE_15EG_Mt-LDPE_10EG_Lap	2,333	5,041	,463	,643	1,000
LDPE_15EG_Mt-LDPE_10EG_Mt	2,667	5,041	,529	,597	1,000

LDPE_15EG_Mt-LDPE_5EG_Mt	5,667	5,041	1,124	,261	1,000
LDPE_15EG_Mt-LDPE_5EG_Lap	9,333	5,041	1,851	,064	1,000
LDPE-LDPE_10EG_Lap	-1,333	5,041	-,264	,791	1,000
LDPE-LDPE_10EG_Mt	-1,667	5,041	-,331	,741	1,000
LDPE-LDPE_5EG_Mt	-4,667	5,041	-,926	,355	1,000
LDPE-LDPE_5EG_Lap	-8,333	5,041	-1,653	,098	1,000
LDPE_10EG_Lap-LDPE_10EG_Mt	-,333	5,041	-,066	,947	1,000
LDPE_10EG_Lap-LDPE_5EG_Mt	-3,333	5,041	-,661	,508	1,000
LDPE_10EG_Lap-LDPE_5EG_Lap	7,000	5,041	1,388	,165	1,000
LDPE_10EG_Mt-LDPE_5EG_Mt	3,000	5,041	,595	,552	1,000
LDPE_10EG_Mt-LDPE_5EG_Lap	6,667	5,041	1,322	,186	1,000
LDPE_5EG_Mt-LDPE_5EG_Lap	3,667	5,041	,727	,467	1,000

Each row tests the null hypothesis that the Sample 1 and Sample 2 distributions are the same.
Asymptotic significances (2-sided tests) are displayed. The significance level is ,050.

a. Significance values have been adjusted by the Bonferroni correction for multiple tests.

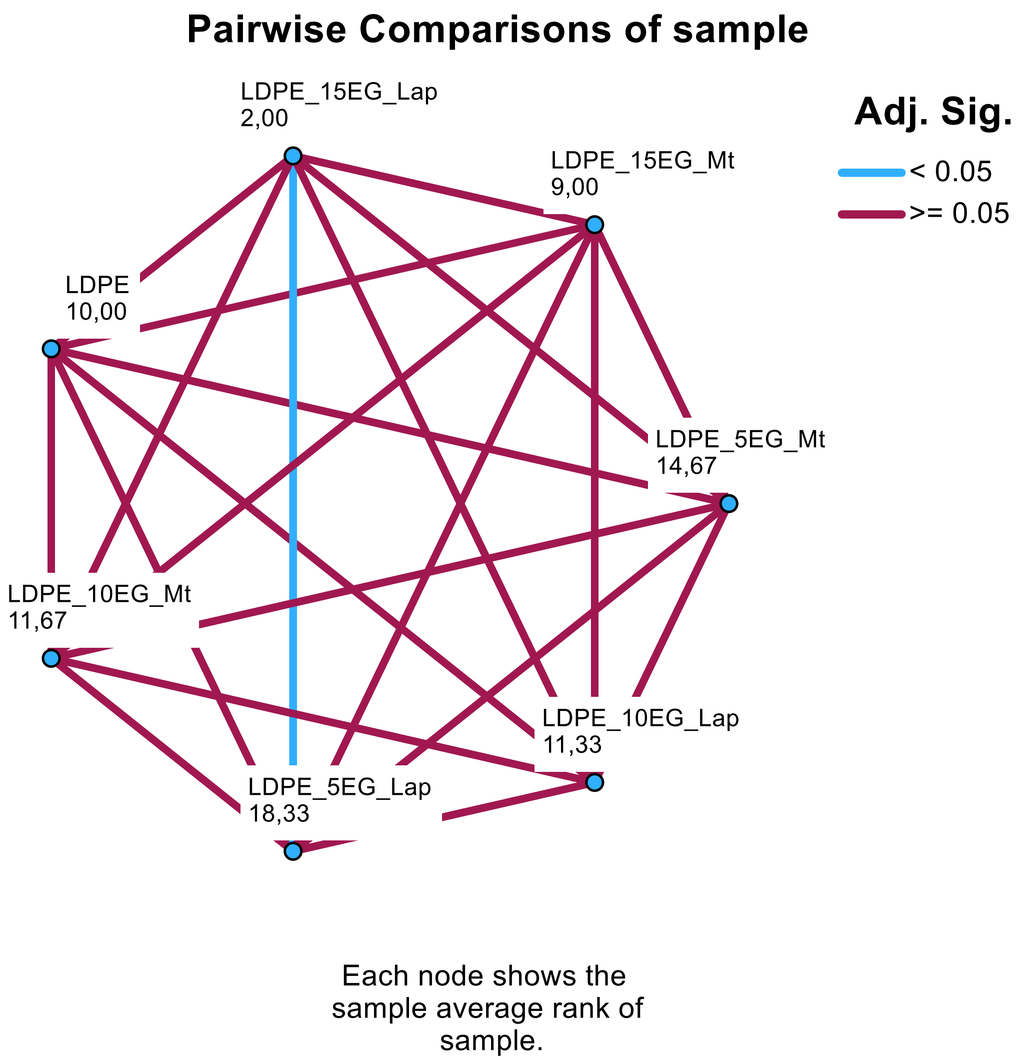

ε_elongation across sample

Independent-Samples Kruskal-Wallis Test Summary

Total N	21
Test Statistic	19,186[a]
Degree Of Freedom	6

Asymptotic Sig.(2-sided test)	,004

a. The test statistic is adjusted for ties.

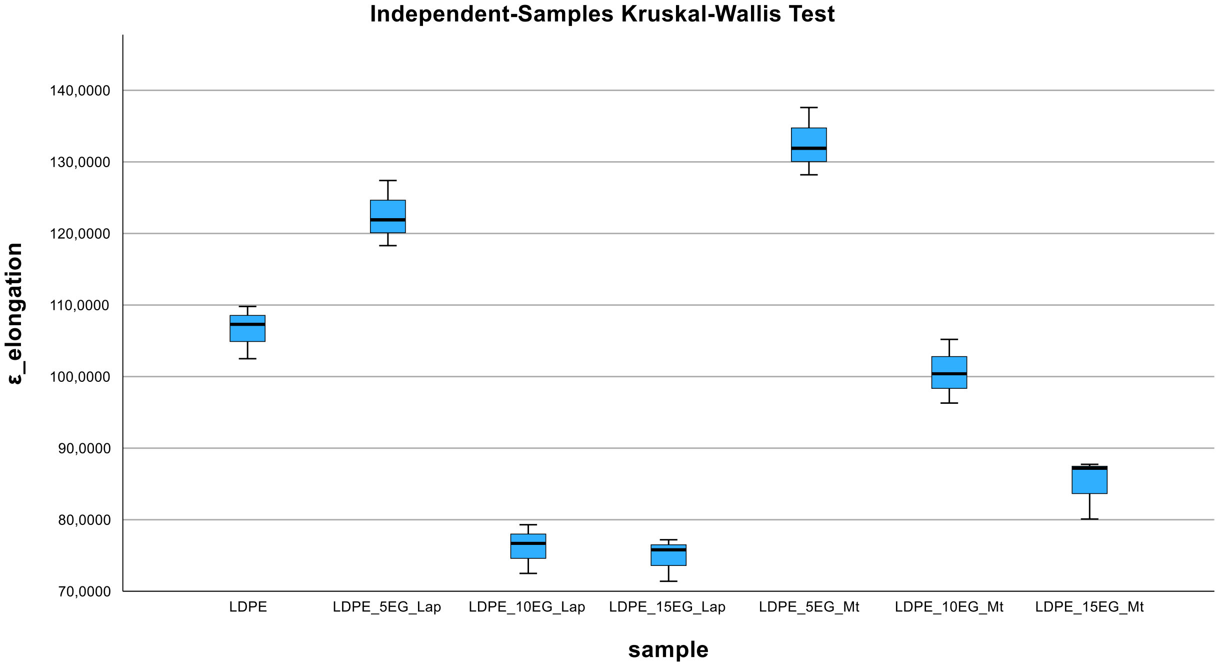

Pairwise Comparisons of sample

Sample 1-Sample 2	Test Statistic	Std. Error	Std. Test Statistic	Sig.	Adj. Sig.[a]
LDPE_15EG_Lap-LDPE_10EG_Lap	1,000	5,066	,197	,844	1,000
LDPE_15EG_Lap-LDPE_15EG_Mt	-5,000	5,066	-,987	,324	1,000
LDPE_15EG_Lap-LDPE_10EG_Mt	-8,333	5,066	-1,645	,100	1,000
LDPE_15EG_Lap-LDPE	10,667	5,066	2,105	,035	,740
LDPE_15EG_Lap-LDPE_5EG_Lap	14,000	5,066	2,763	,006	,120
LDPE_15EG_Lap-LDPE_5EG_Mt	-17,000	5,066	-3,356	<,001	,017
LDPE_10EG_Lap-LDPE_15EG_Mt	-4,000	5,066	-,790	,430	1,000
LDPE_10EG_Lap-LDPE_10EG_Mt	-7,333	5,066	-1,447	,148	1,000
LDPE_10EG_Lap-LDPE	9,667	5,066	1,908	,056	1,000

LDPE_10EG_Lap-LDPE_5EG_Lap	13,000	5,066	2,566	,010	,216
LDPE_10EG_Lap-LDPE_5EG_Mt	-16,000	5,066	-3,158	,002	,033
LDPE_15EG_Mt-LDPE_10EG_Mt	3,333	5,066	,658	,511	1,000
LDPE_15EG_Mt-LDPE	5,667	5,066	1,119	,263	1,000
LDPE_15EG_Mt-LDPE_5EG_Lap	9,000	5,066	1,776	,076	1,000
LDPE_15EG_Mt-LDPE_5EG_Mt	12,000	5,066	2,369	,018	,375
LDPE_10EG_Mt-LDPE	2,333	5,066	,461	,645	1,000
LDPE_10EG_Mt-LDPE_5EG_Lap	5,667	5,066	1,119	,263	1,000
LDPE_10EG_Mt-LDPE_5EG_Mt	8,667	5,066	1,711	,087	1,000
LDPE-LDPE_5EG_Lap	-3,333	5,066	-,658	,511	1,000
LDPE-LDPE_5EG_Mt	-6,333	5,066	-1,250	,211	1,000
LDPE_5EG_Lap-LDPE_5EG_Mt	-3,000	5,066	-,592	,554	1,000

Each row tests the null hypothesis that the Sample 1 and Sample 2 distributions are the same.
Asymptotic significances (2-sided tests) are displayed. The significance level is ,050.

a. Significance values have been adjusted by the Bonferroni correction for multiple tests.

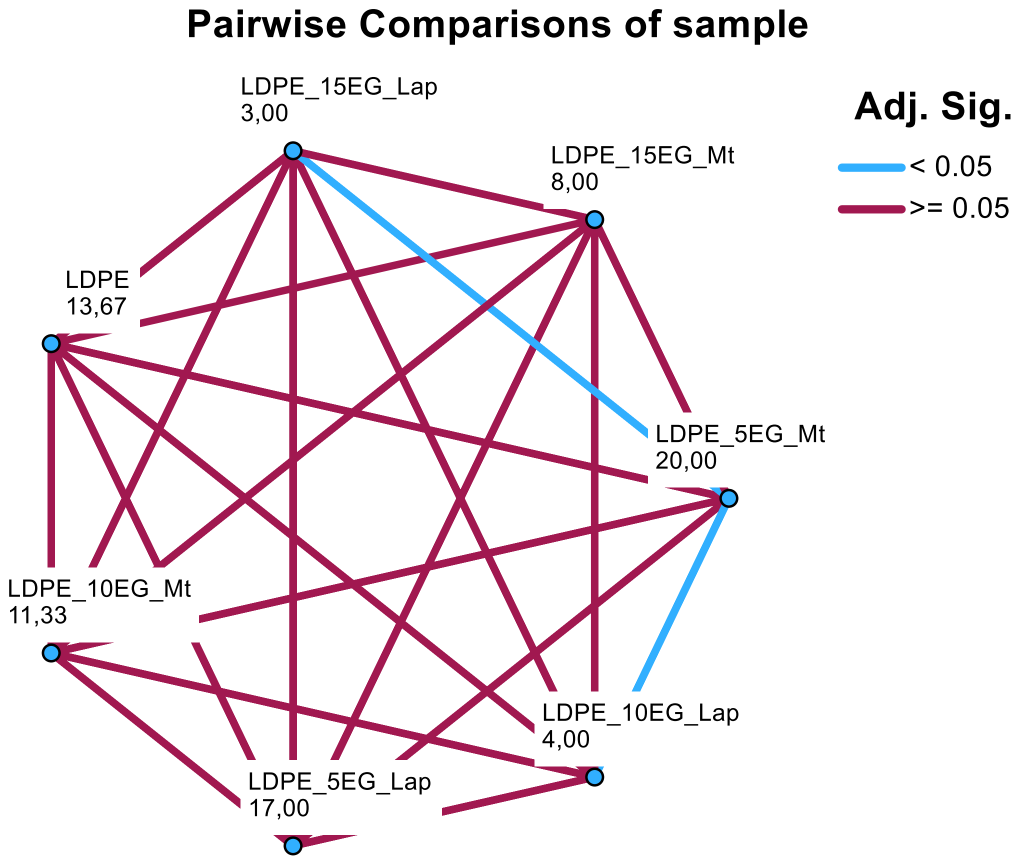

Each node shows the sample average rank of sample.

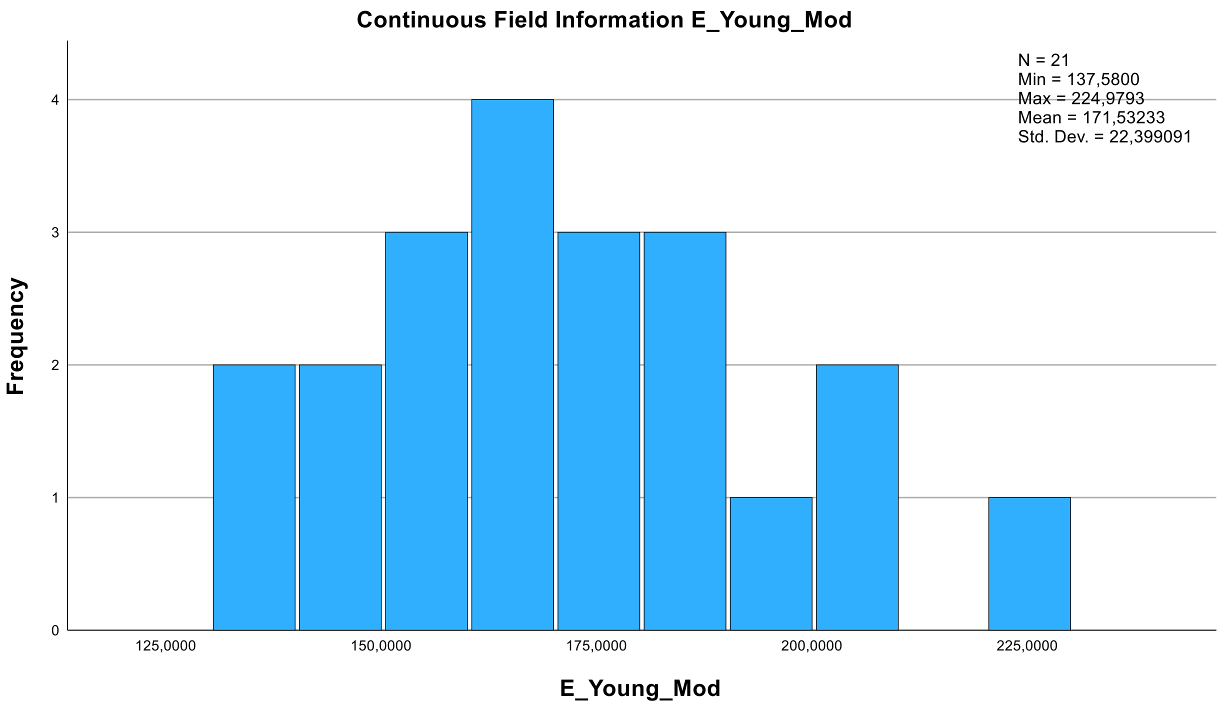
Continuous Field Information E_Young_Mod
N = 21
Min = 137,5800
Max = 224,9793
Mean = 171,53233
Std. Dev. = 22,399091
Frequency
0
1
2
3
4
125,0000
150,0000
175,0000
200,0000
225,0000
E_Young_Mod

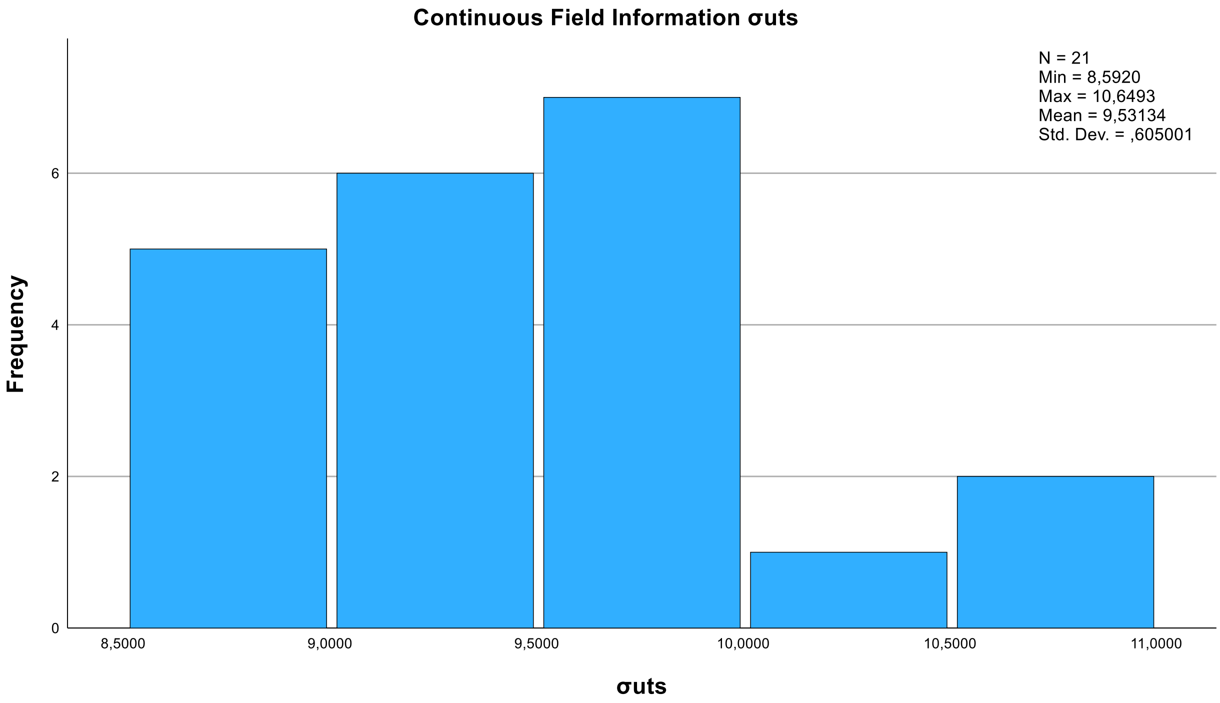
Continuous Field Information σuts
N = 21
Min = 8,5920
Max = 10,6493
Mean = 9,53134
Std. Dev. = ,605001
Frequency
0
2
4
6
8,5000
9,0000
9,5000
10,0000
10,5000
11,0000
σuts

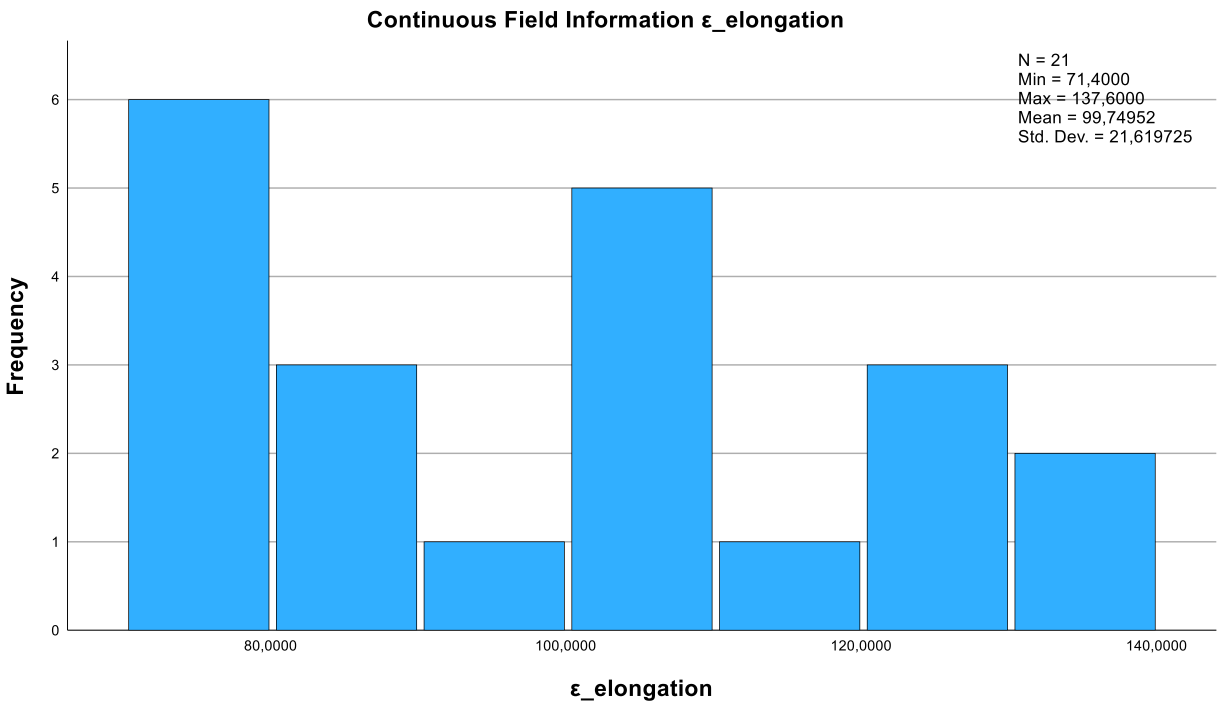
Continuous Field Information ε_elongation
N = 21
Min = 71,4000
Max = 137,6000
Mean = 99,74952
Std. Dev. = 21,619725
Frequency
6
5
4
3
2
1
0
80,0000
100,0000
120,0000
140,0000
ε_elongation

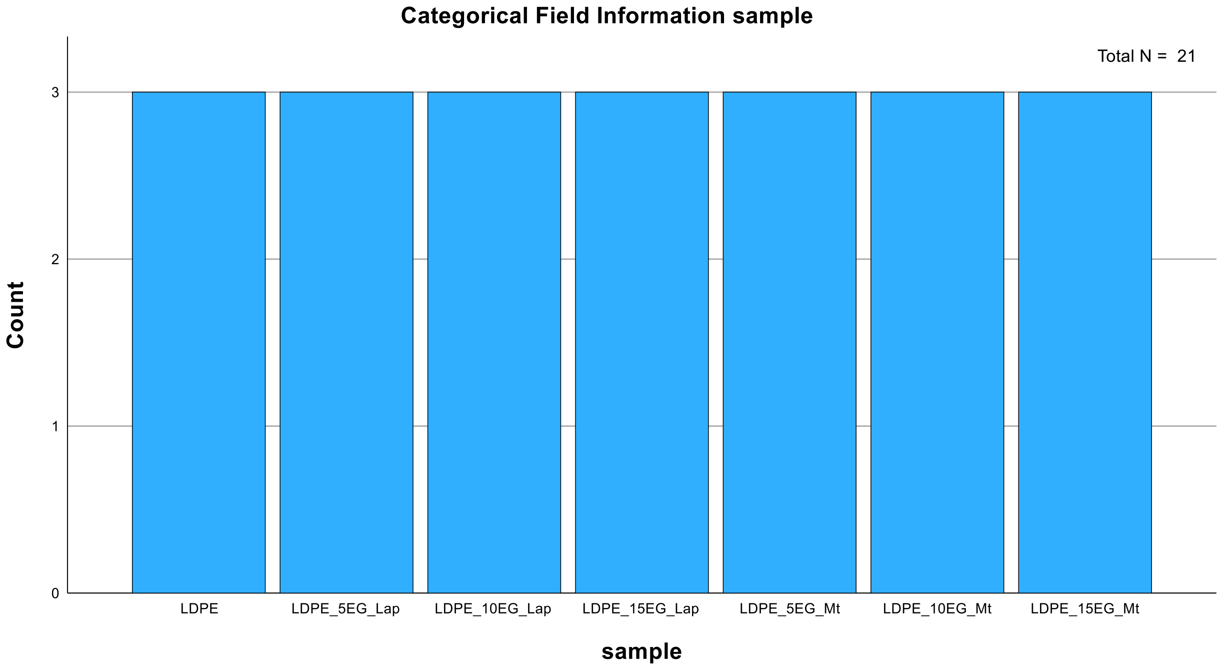
Categorical Field Information sample
Total N = 21
Count
3
2
1
0
LDPE
LDPE_5EG_Lap
LDPE_10EG_Lap
LDPE_15EG_Lap
LDPE_5EG_Mt
LDPE_10EG_Mt
LDPE_15EG_Mt
sample

STATISTICAL ANALYSIS FOR WVTR RESULTS

Explore

Notes

Output Created		18-OCT-2024 09:40:58
Comments		
Input	Data	G:\To Drive μου\1. PAPERS\64. Cornell Eugenol Nanomaterials\STATISTICS \WVTR.sav
	Active Dataset	DataSet1
	Filter	<none>
	Weight	<none>
	Split File	<none>
	N of Rows in Working Data File	21
Missing Value Handling	Definition of Missing	User-defined missing values for dependent variables are treated as missing.
	Cases Used	Statistics are based on cases with no missing values for any dependent variable or factor used.
Syntax		EXAMINE VARIABLES=Diff_Coef BY sample<br>/PLOT NONE<br>/STATISTICS DESCRIPTIVES<br>/CINTERVAL 95<br>/MISSING LISTWISE<br>/NOTOTAL.
Resources	Processor Time	00:00:00.00
	Elapsed Time	00:00:00.03

[DataSet1] G:\To Drive μου\1. PAPERS\64. Cornell Eugenol

Nanomaterials\STATISTICS\WVTR.sav

sample

Case Processing Summary

		Cases					
		Valid		Missing		Total	
	sample	N	Percent	N	Percent	N	Percent
Diff_Coef	LDPE	3	100,0%	0	0,0%	3	100,0%
	LDPE_5EG_Lap	3	100,0%	0	0,0%	3	100,0%
	LDPE_10EG_Lap	3	100,0%	0	0,0%	3	100,0%
	LDPE_15EG_Lap	3	100,0%	0	0,0%	3	100,0%
	LDPE_5EG_Mt	3	100,0%	0	0,0%	3	100,0%
	LDPE_10EG_Mt	3	100,0%	0	0,0%	3	100,0%
	LDPE_15EG_Mt	3	100,0%	0	0,0%	3	100,0%

Descriptives

	sample			Statistic	Std. Error
Diff_Coef	LDPE	Mean		4,874537e-004	5,4664316e-005
		95% Confidence Interval for Mean	Lower Bound	2,522521e-004	
			Upper Bound	7,226553e-004	
		5% Trimmed Mean		.	
		Median		4,537683e-004	
		Variance		,000	
		Std. Deviation		9,4681373e-005	
		Minimum		4,1422e-004	
		Maximum		5,9437e-004	
		Range		1,8015e-004	
		Interquartile Range		.	
		Skewness		1,398	1,225
		Kurtosis		.	.

	LDPE_5EG_Lap	Mean		9,281195e-005	1,9793058e-005
		95% Confidence Interval for Mean	Lower Bound	7,649291e-006	
			Upper Bound	1,779746e-004	
		5% Trimmed Mean		.	
		Median		1,093504e-004	
		Variance		,000	
		Std. Deviation		3,4282583e-005	
		Minimum		5,3395e-005	
		Maximum		1,1569e-004	
		Range		6,2295e-005	
		Interquartile Range		.	
		Skewness		-1,666	1,225
		Kurtosis		.	.
	LDPE_10EG_Lap	Mean		1,110997e-004	3,9312144e-005
		95% Confidence Interval for Mean	Lower Bound	-5,804683e-005	
			Upper Bound	2,802462e-004	
		5% Trimmed Mean		.	
		Median		1,078311e-004	
		Variance		,000	
		Std. Deviation		6,8090630e-005	
		Minimum		4,4702e-005	
		Maximum		1,8077e-004	
		Range		1,3606e-004	
		Interquartile Range		.	
		Skewness		,216	1,225
		Kurtosis		.	.
	LDPE_15EG_Lap	Mean		1,170291e-004	2,5351280e-005
		95% Confidence Interval for Mean	Lower Bound	7,951307e-006	
			Upper Bound	2,261068e-004	
		5% Trimmed Mean		.	

		Median		1,400806e-004	
		Variance		,000	
		Std. Deviation		4,3909704e-005	
		Minimum		6,6394e-005	
		Maximum		1,4461e-004	
		Range		7,8219e-005	
		Interquartile Range		.	
		Skewness		-1,711	1,225
		Kurtosis		.	.
	LDPE_5EG_Mt	Mean		1,294114e-004	3,5842212e-005
		95% Confidence Interval for Mean	Lower Bound	-2,480517e-005	
			Upper Bound	2,836280e-004	
		5% Trimmed Mean		.	
		Median		1,524921e-004	
		Variance		,000	
		Std. Deviation		6,2080532e-005	
		Minimum		5,9096e-005	
		Maximum		1,7665e-004	
		Range		1,1755e-004	
		Interquartile Range		.	
		Skewness		-1,442	1,225
		Kurtosis		.	.
	LDPE_10EG_Mt	Mean		8,841383e-005	1,2379310e-005
		95% Confidence Interval for Mean	Lower Bound	3,514996e-005	
			Upper Bound	1,416777e-004	
		5% Trimmed Mean		.	
		Median		8,777179e-005	
		Variance		,000	
		Std. Deviation		2,1441595e-005	
		Minimum		6,7300e-005	

		Maximum		1,1017e-004	
		Range		4,2869e-005	
		Interquartile Range		.	
		Skewness		,135	1,225
		Kurtosis		.	.
	LDPE_15EG_Mt	Mean		6,438555e-005	2,3923769e-005
		95% Confidence Interval for Mean	Lower Bound	-3,855012e-005	
			Upper Bound	1,673212e-004	
		5% Trimmed Mean		.	
		Median		7,201788e-005	
		Variance		,000	
		Std. Deviation		4,1437183e-005	
		Minimum		1,9663e-005	
		Maximum		1,0148e-004	
		Range		8,1813e-005	
		Interquartile Range		.	
		Skewness		-,801	1,225
		Kurtosis		.	.

Nonparametric Tests

Notes

Output Created		18-OCT-2024 09:41:24
Comments		
Input	Data	G:\To Drive μου\1. PAPERS\64. Cornell Eugenol Nanomaterials\STATISTICS \WVTR.sav
	Active Dataset	DataSet1
	Filter	<none>
	Weight	<none>
	Split File	<none>

	N of Rows in Working Data File	21
Syntax		NPTESTS /INDEPENDENT TEST (Diff_Coef) GROUP (sample) KRUSKAL_WALLIS(COMPARE=PAIRWISE) /MISSING SCOPE=ANALYSIS USERMISSING=EXCLUDE /CRITERIA ALPHA=0.05 CILEVEL=95.
Resources	Processor Time	00:00:02.77
	Elapsed Time	00:00:01.79

Hypothesis Test Summary

	Null Hypothesis	Test	Sig.[a,b]
1	The distribution of Diff_Coef is the same across categories of sample.	Independent-Samples Kruskal-Wallis Test	,148

Hypothesis Test Summary

	Decision
1	Retain the null hypothesis.

a. The significance level is .050.

b. Asymptotic significance is displayed.

Independent-Samples Kruskal-Wallis Test

Diff_Coef across sample

Independent-Samples Kruskal-Wallis Test Summary

Total N	21
Test Statistic	9,489[a]
Degree Of Freedom	6
Asymptotic Sig.(2-sided test)	,148

a. The test statistic is adjusted for ties.

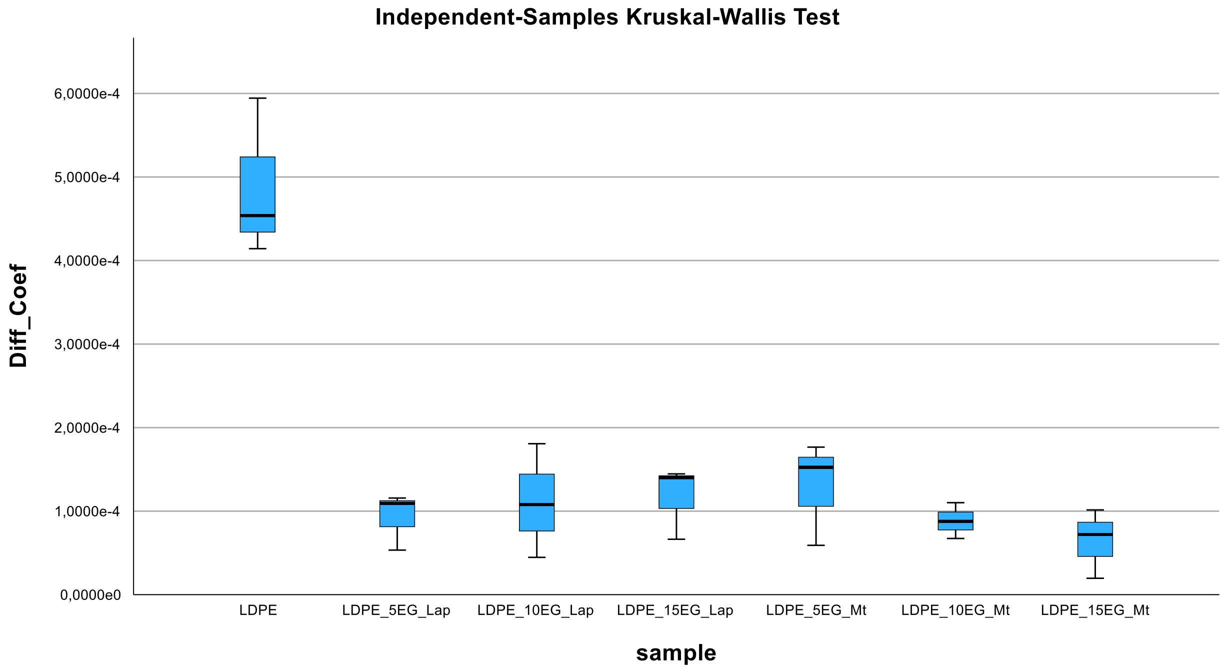

Pairwise Comparisons of sample

Sample 1-Sample 2	Test Statistic	Std. Error	Std. Test Statistic	Sig.	Adj. Sig.[a]
LDPE_15EG_Mt-LDPE_10EG_Mt	3,000	5,066	,592	,554	1,000
LDPE_15EG_Mt-LDPE_5EG_Lap	3,333	5,066	,658	,511	1,000
LDPE_15EG_Mt-LDPE_10EG_Lap	4,333	5,066	,855	,392	1,000
LDPE_15EG_Mt-LDPE_15EG_Lap	5,667	5,066	1,119	,263	1,000
LDPE_15EG_Mt-LDPE_5EG_Mt	6,667	5,066	1,316	,188	1,000

LDPE_15EG_Mt-LDPE	14,333	5,066	2,829	,005	,098
LDPE_10EG_Mt-LDPE_5EG_Lap	,333	5,066	,066	,948	1,000
LDPE_10EG_Mt-LDPE_10EG_Lap	1,333	5,066	,263	,792	1,000
LDPE_10EG_Mt-LDPE_15EG_Lap	2,667	5,066	,526	,599	1,000
LDPE_10EG_Mt-LDPE_5EG_Mt	3,667	5,066	,724	,469	1,000
LDPE_10EG_Mt-LDPE	11,333	5,066	2,237	,025	,531
LDPE_5EG_Lap-LDPE_10EG_Lap	-1,000	5,066	-,197	,844	1,000
LDPE_5EG_Lap-LDPE_15EG_Lap	-2,333	5,066	-,461	,645	1,000
LDPE_5EG_Lap-LDPE_5EG_Mt	-3,333	5,066	-,658	,511	1,000
LDPE_5EG_Lap-LDPE	11,000	5,066	2,171	,030	,628
LDPE_10EG_Lap-LDPE_15EG_Lap	-1,333	5,066	-,263	,792	1,000
LDPE_10EG_Lap-LDPE_5EG_Mt	-2,333	5,066	-,461	,645	1,000
LDPE_10EG_Lap-LDPE	10,000	5,066	1,974	,048	1,000
LDPE_15EG_Lap-LDPE_5EG_Mt	-1,000	5,066	-,197	,844	1,000
LDPE_15EG_Lap-LDPE	8,667	5,066	1,711	,087	1,000
LDPE_5EG_Mt-LDPE	7,667	5,066	1,513	,130	1,000

Each row tests the null hypothesis that the Sample 1 and Sample 2 distributions are the same.
Asymptotic significances (2-sided tests) are displayed. The significance level is .050.

a. Significance values have been adjusted by the Bonferroni correction for multiple tests.

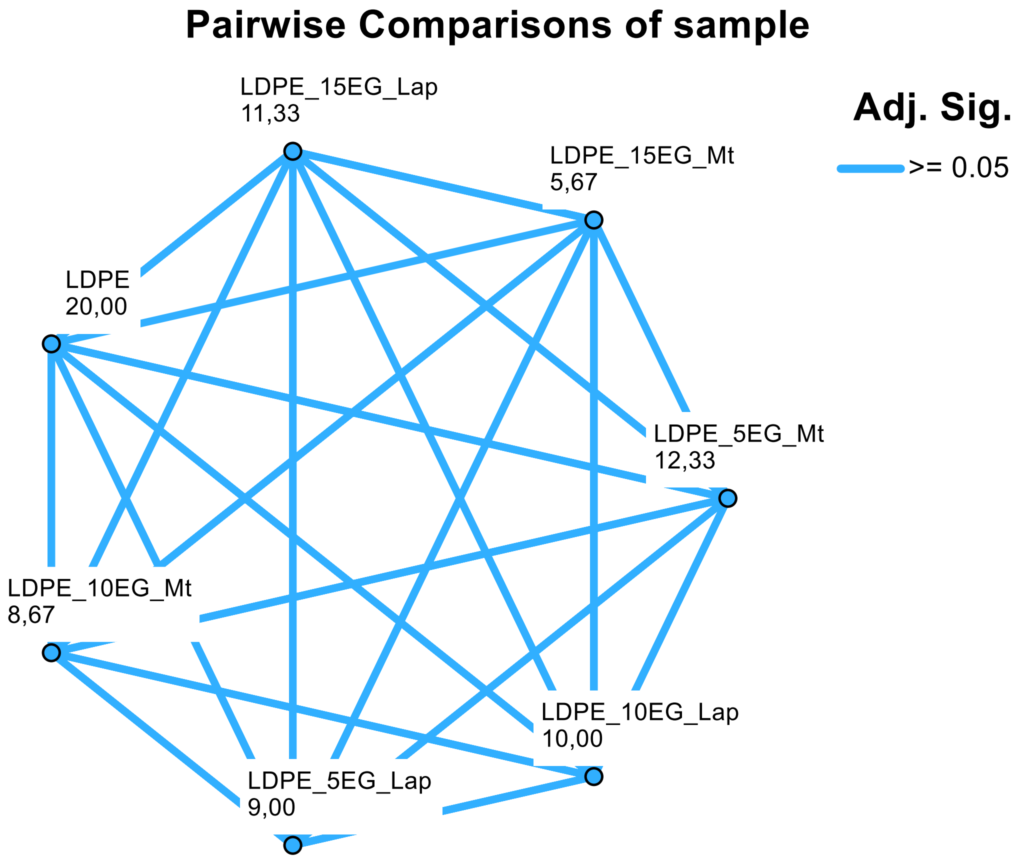

Each node shows the sample average rank of sample.

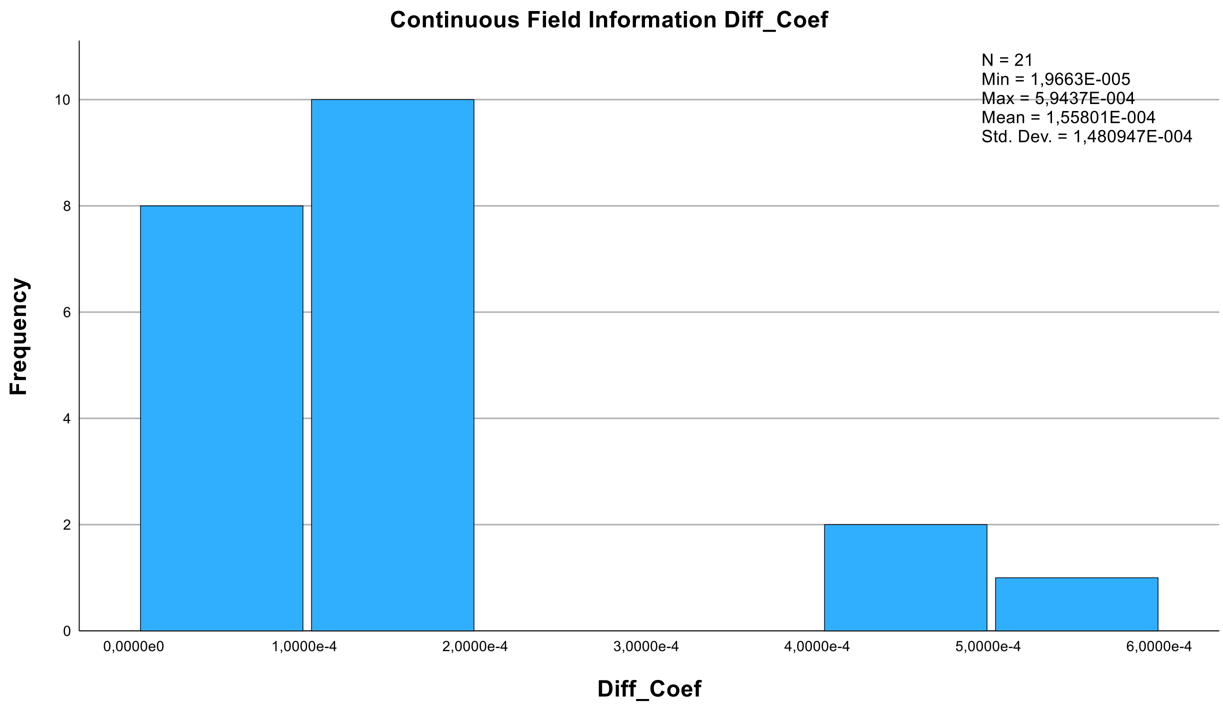
Continuous Field Information Diff_Coef
N = 21
Min = 1,9663E-005
Max = 5,9437E-004
Mean = 1,55801E-004
Std. Dev. = 1,480947E-004
Frequency
0
2
4
6
8
10
0,0000e0
1,0000e-4
2,0000e-4
3,0000e-4
4,0000e-4
5,0000e-4
6,0000e-4
Diff_Coef

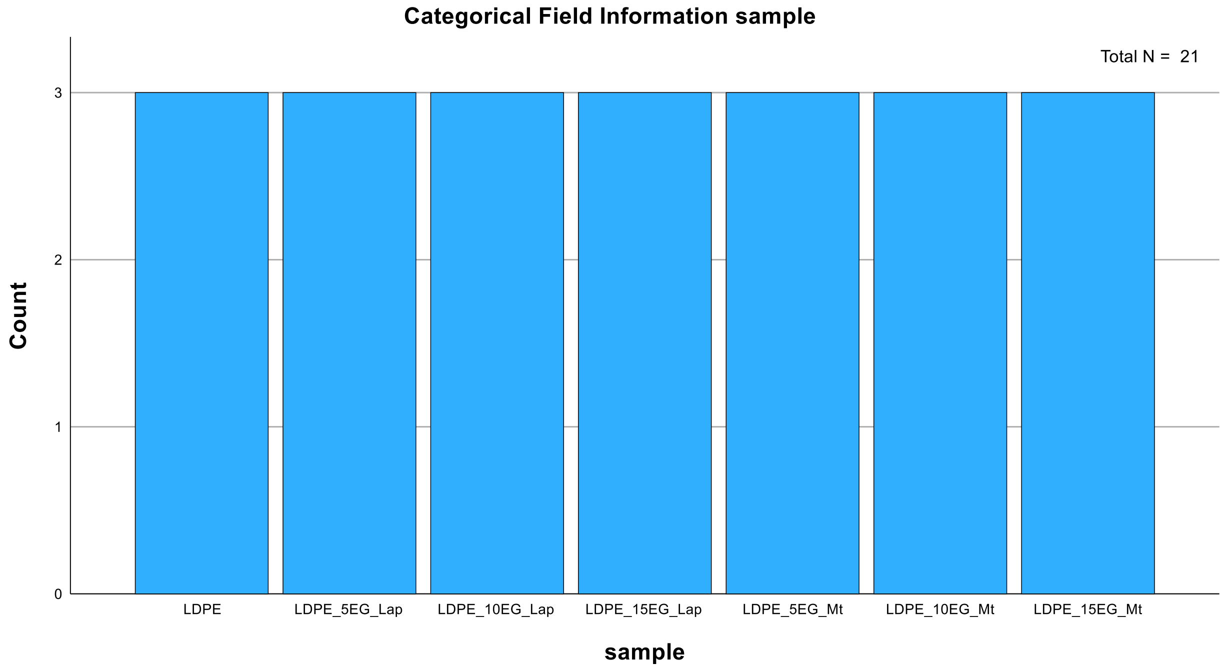
Categorical Field Information sample
Total N = 21
Count
0
1
2
3
LDPE
LDPE_5EG_Lap
LDPE_10EG_Lap
LDPE_15EG_Lap
LDPE_5EG_Mt
LDPE_10EG_Mt
LDPE_15EG_Mt
sample

STATISTICAL ANALYSIS FOR OTR

Explore

Notes

Output Created		18-OCT-2024 09:37:21
Comments		
Input	Data	G:\To Drive μου\1. PAPERS\64. Cornell Eugenol Nanomaterials\STATISTICS \OTR.sav
	Active Dataset	DataSet1
	Filter	<none>
	Weight	<none>
	Split File	<none>
	N of Rows in Working Data File	21
Missing Value Handling	Definition of Missing	User-defined missing values for dependent variables are treated as missing.
	Cases Used	Statistics are based on cases with no missing values for any dependent variable or factor used.
Syntax		EXAMINE VARIABLES=Perm_Coef BY sample /PLOT NONE /STATISTICS DESCRIPTIVES /CINTERVAL 95 /MISSING LISTWISE /NOTOTAL.
Resources	Processor Time	00:00:00.00
	Elapsed Time	00:00:00.02

[DataSet1] G:\To Drive μου\1. PAPERS\64. Cornell Eugenol Nanomaterials\STATISTICS\OTR.sav

sample

Case Processing Summary

		Cases					
		Valid		Missing		Total	
	sample	N	Percent	N	Percent	N	Percent
Perm_Coef	LDPE	3	100,0%	0	0,0%	3	100,0%
	LDPE_5EG_Lap	3	100,0%	0	0,0%	3	100,0%
	LDPE_10EG_Lap	3	100,0%	0	0,0%	3	100,0%
	LDPE_15EG_Lap	3	100,0%	0	0,0%	3	100,0%
	LDPE_5EG_Mt	3	100,0%	0	0,0%	3	100,0%
	LDPE_10EG_Mt	3	100,0%	0	0,0%	3	100,0%
	LDPE_15EG_Mt	3	100,0%	0	0,0%	3	100,0%

Descriptives

	sample			Statistic	Std. Error
Perm_Coef	LDPE	Mean		2,610163e-008	7,4713166e-011
		95% Confidence Interval for Mean	Lower Bound	2,578016e-008	
			Upper Bound	2,642309e-008	
		5% Trimmed Mean		.	
		Median		2,610163e-008	
		Variance		,000	
		Std. Deviation		1,2940700e-010	
		Minimum		2,5972e-008	
		Maximum		2,6231e-008	
		Range		2,5881e-010	
		Interquartile Range		.	

		Skewness		,000	1,225
		Kurtosis		.	.
	LDPE_5EG_Lap	Mean		1,765663e-008	1,9819165e-009
		95% Confidence Interval for Mean	Lower Bound	9,129132e-009	
			Upper Bound	2,618413e-008	
		5% Trimmed Mean		.	
		Median		1,765663e-008	
		Variance		,000	
		Std. Deviation		3,4327800e-009	
		Minimum		1,4224e-008	
		Maximum		2,1089e-008	
		Range		6,8656e-009	
		Interquartile Range		.	
		Skewness		,000	1,225
		Kurtosis		.	.
	LDPE_10EG_Lap	Mean		2,343251e-008	4,7398350e-010
		95% Confidence Interval for Mean	Lower Bound	2,139312e-008	
			Upper Bound	2,547190e-008	
		5% Trimmed Mean		.	
		Median		2,343251e-008	
		Variance		,000	
		Std. Deviation		8,2096350e-010	
		Minimum		2,2612e-008	
		Maximum		2,4253e-008	
		Range		1,6419e-009	
		Interquartile Range		.	
		Skewness		,000	1,225
		Kurtosis		.	.
	LDPE_15EG_Lap	Mean		2,031005e-008	7,8324377e-010
		95% Confidence Interval for Mean	Lower Bound	1,694002e-008	
			Upper Bound	2,368007e-008	

		5% Trimmed Mean		.	
		Median		2,031005e-008	
		Variance		,000	
		Std. Deviation		1,3566180e-009	
		Minimum		1,8953e-008	
		Maximum		2,1667e-008	
		Range		2,7132e-009	
		Interquartile Range		.	
		Skewness		,000	1,225
		Kurtosis		.	.
	LDPE_5EG_Mt	Mean		1,835860e-008	1,4766830e-010
		95% Confidence Interval for Mean	Lower Bound	1,772323e-008	
			Upper Bound	1,899396e-008	
		5% Trimmed Mean		.	
		Median		1,835860e-008	
		Variance		,000	
		Std. Deviation		2,5576900e-010	
		Minimum		1,8103e-008	
		Maximum		1,8614e-008	
		Range		5,1154e-010	
		Interquartile Range		.	
		Skewness		,000	1,225
		Kurtosis		.	.
	LDPE_10EG_Mt	Mean		2,077500e-008	6,7357724e-011
		95% Confidence Interval for Mean	Lower Bound	2,048518e-008	
			Upper Bound	2,106482e-008	
		5% Trimmed Mean		.	
		Median		2,077500e-008	
		Variance		,000	
		Std. Deviation		1,1666700e-010	

		Minimum		2,0658e-008	
		Maximum		2,0892e-008	
		Range		2,3333e-010	
		Interquartile Range		.	
		Skewness		,000	1,225
		Kurtosis		.	.
	LDPE_15EG_Mt	Mean		2,516348e-008	1,5302141e-009
		95% Confidence Interval for Mean	Lower Bound	1,857950e-008	
			Upper Bound	3,174746e-008	
		5% Trimmed Mean		.	
		Median		2,516348e-008	
		Variance		,000	
		Std. Deviation		2,6504085e-009	
		Minimum		2,2513e-008	
		Maximum		2,7814e-008	
		Range		5,3008e-009	
		Interquartile Range		.	
		Skewness		,000	1,225
		Kurtosis		.	.

Nonparametric Tests

Notes

Output Created		18-OCT-2024 09:37:50
Comments		
Input	Data	G:\To Drive μου\1. PAPERS\64. Cornell Eugenol Nanomaterials\STATISTICS \OTR.sav
	Active Dataset	DataSet1
	Filter	<none>

	Weight	<none>
	Split File	<none>
	N of Rows in Working Data File	21
Syntax		NPTESTS /INDEPENDENT TEST (Perm_Coef) GROUP (sample) KRUSKAL_WALLIS(COMPARE=PAIRWISE) /MISSING SCOPE=ANALYSIS USERMISSING=EXCLUDE /CRITERIA ALPHA=0.05 CILEVEL=95.
Resources	Processor Time	00:00:02.30
	Elapsed Time	00:00:01.58

Hypothesis Test Summary

	Null Hypothesis	Test	Sig.[a,b]
1	The distribution of Perm_Coef is the same across categories of sample.	Independent-Samples Kruskal-Wallis Test	,010

Hypothesis Test Summary

	Decision
1	Reject the null hypothesis.

a. The significance level is ,050.

b. Asymptotic significance is displayed.

Independent-Samples Kruskal-Wallis Test

Perm_Coef across sample

Independent-Samples Kruskal-Wallis Test Summary

Total N	21
Test Statistic	16,848[a]
Degree Of Freedom	6
Asymptotic Sig.(2-sided test)	,010

a. The test statistic is adjusted for ties.

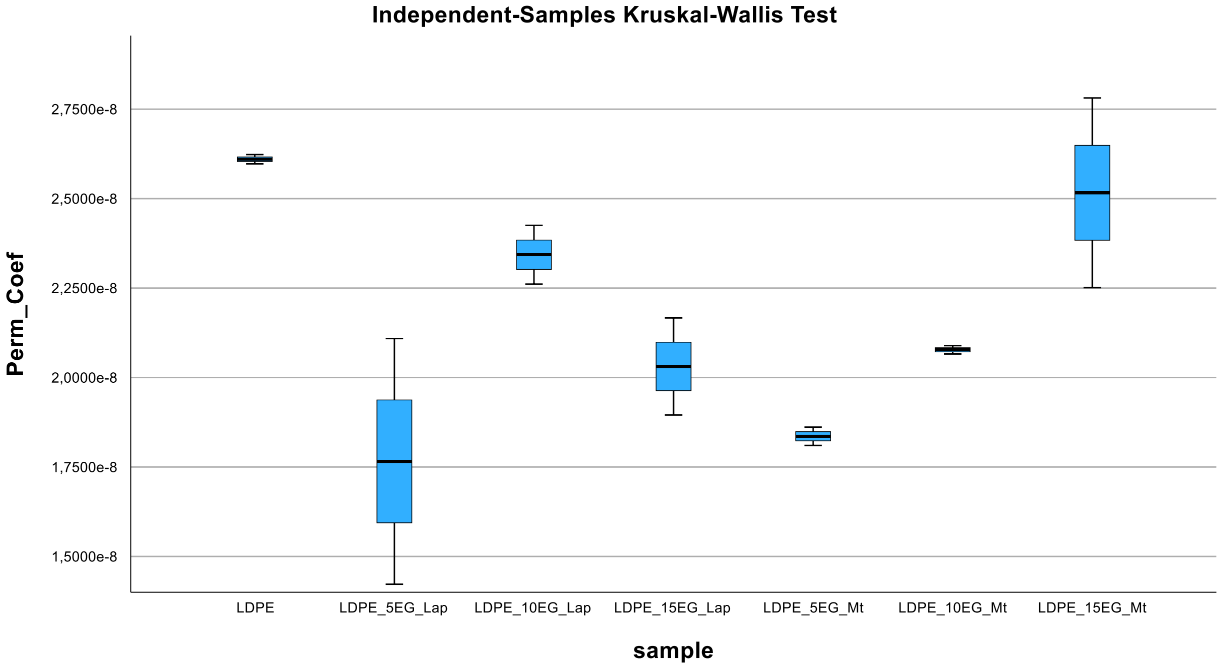

Pairwise Comparisons of sample

Sample 1-Sample 2	Test Statistic	Std. Error	Std. Test Statistic	Sig.	Adj. Sig.[a]
LDPE_5EG_Mt-LDPE_5EG_Lap	,667	5,066	,132	,895	1,000
LDPE_5EG_Mt-LDPE_15EG_Lap	4,333	5,066	,855	,392	1,000
LDPE_5EG_Mt-LDPE_10EG_Mt	-5,000	5,066	-,987	,324	1,000

LDPE_5EG_Mt-LDPE_10EG_Lap	11,000	5,066	2,171	,030	,628
LDPE_5EG_Mt-LDPE_15EG_Mt	-13,000	5,066	-2,566	,010	,216
LDPE_5EG_Mt-LDPE	15,000	5,066	2,961	,003	,064
LDPE_5EG_Lap-LDPE_15EG_Lap	-3,667	5,066	-,724	,469	1,000
LDPE_5EG_Lap-LDPE_10EG_Mt	-4,333	5,066	-,855	,392	1,000
LDPE_5EG_Lap-LDPE_10EG_Lap	-10,333	5,066	-2,040	,041	,869
LDPE_5EG_Lap-LDPE_15EG_Mt	-12,333	5,066	-2,434	,015	,313
LDPE_5EG_Lap-LDPE	14,333	5,066	2,829	,005	,098
LDPE_15EG_Lap-LDPE_10EG_Mt	-,667	5,066	-,132	,895	1,000
LDPE_15EG_Lap-LDPE_10EG_Lap	6,667	5,066	1,316	,188	1,000
LDPE_15EG_Lap-LDPE_15EG_Mt	-8,667	5,066	-1,711	,087	1,000
LDPE_15EG_Lap-LDPE	10,667	5,066	2,105	,035	,740
LDPE_10EG_Mt-LDPE_10EG_Lap	6,000	5,066	1,184	,236	1,000
LDPE_10EG_Mt-LDPE_15EG_Mt	-8,000	5,066	-1,579	,114	1,000
LDPE_10EG_Mt-LDPE	10,000	5,066	1,974	,048	1,000
LDPE_10EG_Lap-LDPE_15EG_Mt	-2,000	5,066	-,395	,693	1,000
LDPE_10EG_Lap-LDPE	4,000	5,066	,790	,430	1,000
LDPE_15EG_Mt-LDPE	2,000	5,066	,395	,693	1,000

Each row tests the null hypothesis that the Sample 1 and Sample 2 distributions are the same.
Asymptotic significances (2-sided tests) are displayed. The significance level is ,050.

a. Significance values have been adjusted by the Bonferroni correction for multiple tests.

Pairwise Comparisons of sample

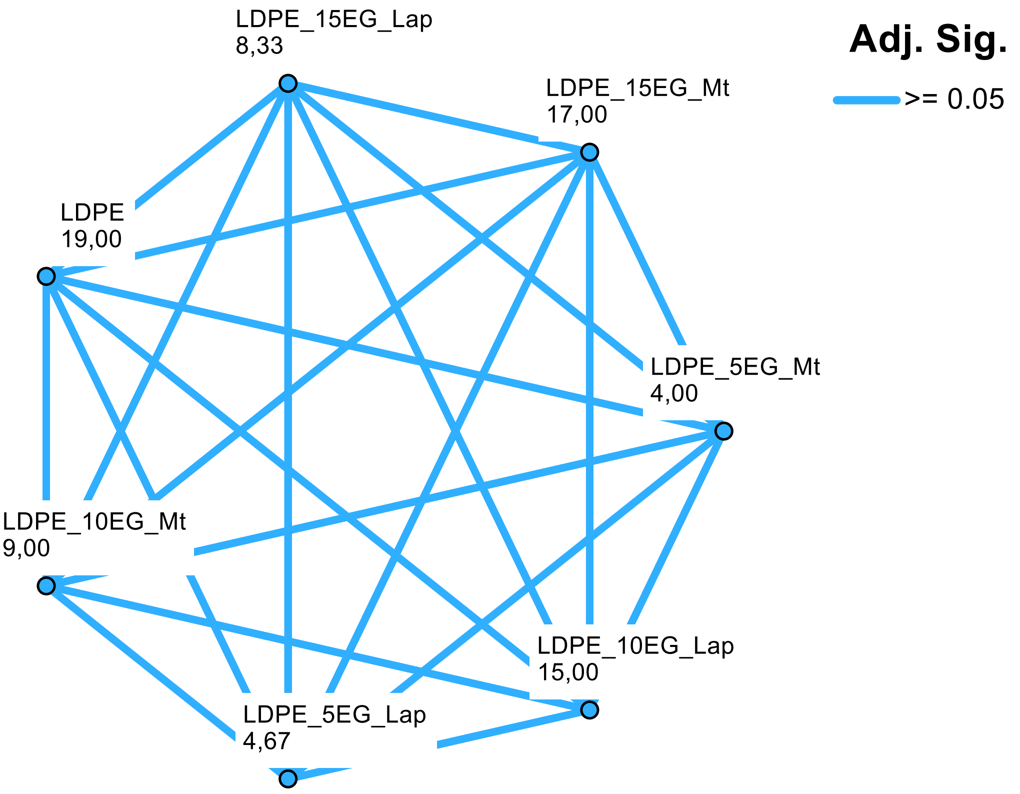

Each node shows the sample average rank of sample.

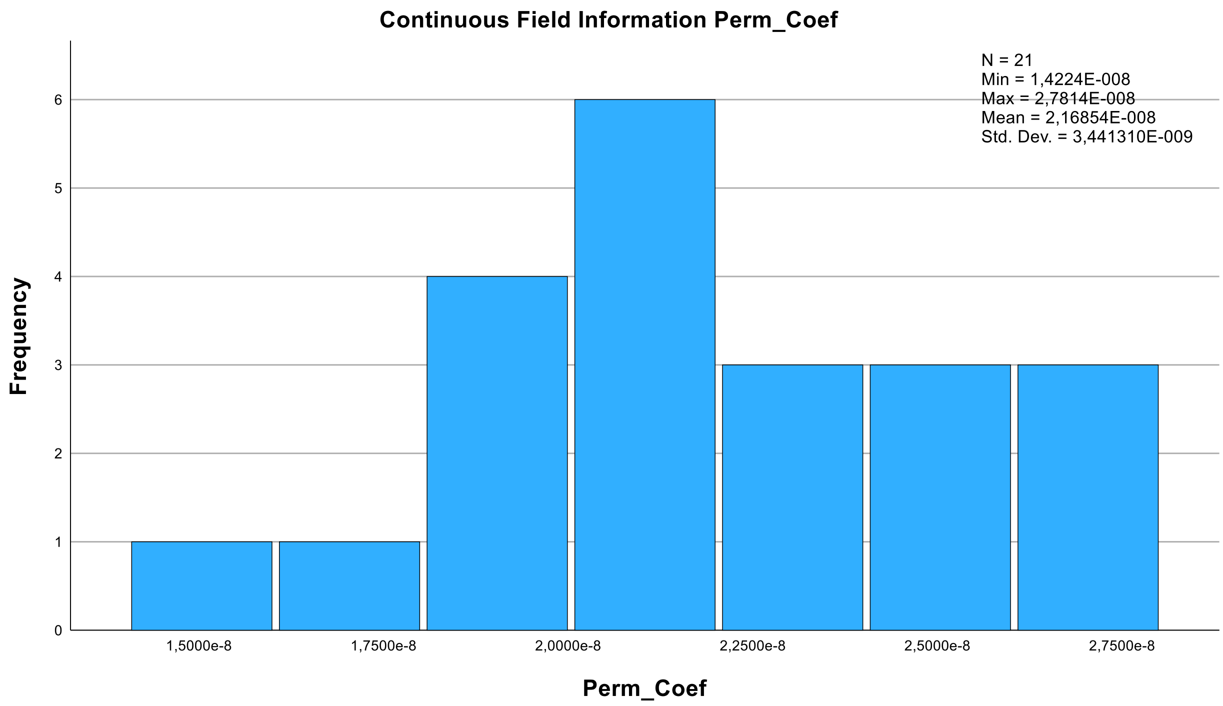
Continuous Field Information Perm_Coef
N = 21
Min = 1,4224E-008
Max = 2,7814E-008
Mean = 2,16854E-008
Std. Dev. = 3,441310E-009
Frequency
0
1
2
3
4
5
6
1,5000e-8
1,7500e-8
2,0000e-8
2,2500e-8
2,5000e-8
2,7500e-8
Perm_Coef

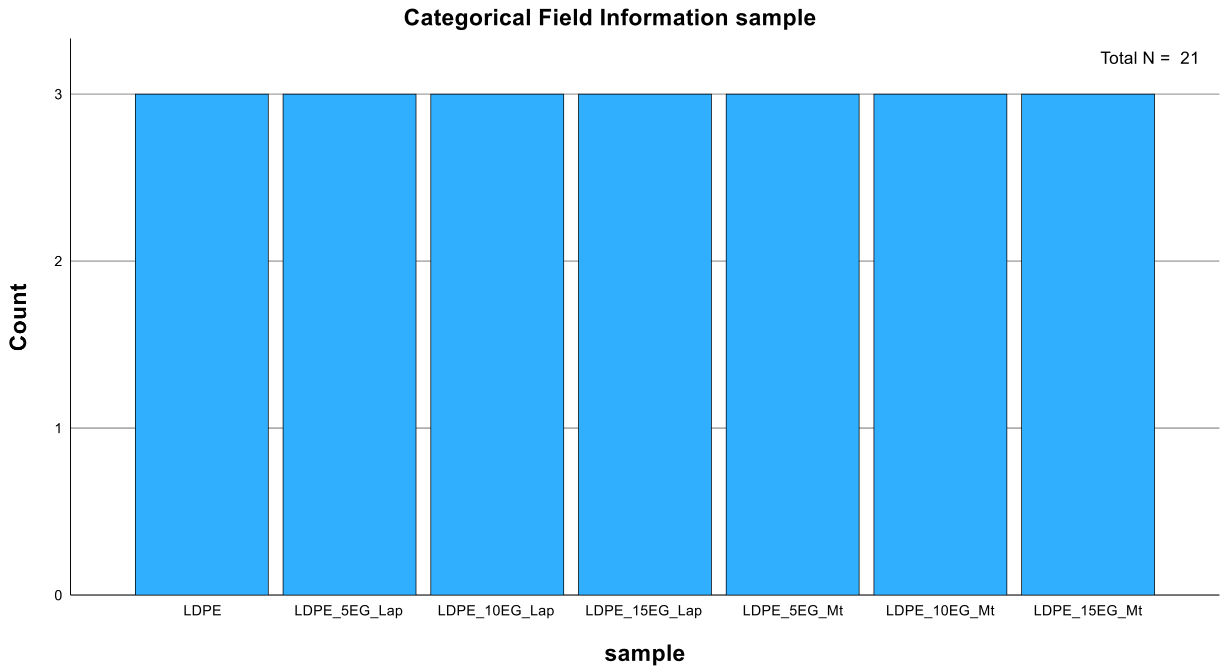
Categorical Field Information sample
Total N = 21
Count
0
1
2
3
LDPE
LDPE_5EG_Lap
LDPE_10EG_Lap
LDPE_15EG_Lap
LDPE_5EG_Mt
LDPE_10EG_Mt
LDPE_15EG_Mt
sample

pH analysis

pH across samples

Supplementary Table 1 pH across samples

Hypothesis Test Summary

	Null Hypothesis	Test	Sig.[a,b]	Decision
1	The distribution of pH_Day_0 is the same across categories of Samples.	Independent-Samples Kruskal-Wallis Test	1,000	Retain the null hypothesis.
2	The distribution of pH_Day_2 is the same across categories of Samples.	Independent-Samples Kruskal-Wallis Test	,172	Retain the null hypothesis.
3	The distribution of pH_Day_4 is the same across categories of Samples.	Independent-Samples Kruskal-Wallis Test	,025	Reject the null hypothesis.
4	The distribution of pH_Day_6 is the same across categories of Samples.	Independent-Samples Kruskal-Wallis Test	,035	Reject the null hypothesis.
5	The distribution of pH_Day_8 is the same across categories of Samples.	Independent-Samples Kruskal-Wallis Test	,063	Retain the null hypothesis.
6	The distribution of pH_Day_10 is the same across categories of Samples.	Independent-Samples Kruskal-Wallis Test	,099	Retain the null hypothesis.

a. The significance level is ,050.

b. Asymptotic significance is displayed.

Day 0

Sample 1-Sample 2	Test Statistic	Std. Error	Std. Test Statistic	Sig.
LDPE-MT	,000	2,121	,000	1,000
LDPE-LAP	,000	2,121	,000	1,000
MT-LAP	,000	2,121	,000	1,000

Each row tests the null hypothesis that the Sample 1 and Sample 2 distributions are the same.

Asymptotic significances (2-sided tests) are displayed. The significance level is ,050.

Day 2

Sample 1-Sample 2	Test Statistic	Std. Error	Std. Test Statistic	Sig.
LAP-MT	,333	2,160	,154	,877
LAP-LDPE	3,667	2,160	1,697	,090
MT-LDPE	3,333	2,160	1,543	,123

Each row tests the null hypothesis that the Sample 1 and Sample 2 distributions are the same.

Asymptotic significances (2-sided tests) are displayed. The significance level is ,050.

Day 4

Sample 1-Sample 2	Test Statistic	Std. Error	Std. Test Statistic	Sig.
MT-LAP	-3,000	2,208	-1,359	,174
MT-LDPE	6,000	2,208	2,717	,007
LAP-LDPE	3,000	2,208	1,359	,174

Each row tests the null hypothesis that the Sample 1 and Sample 2 distributions are the same.

Asymptotic significances (2-sided tests) are displayed. The significance level is ,050.

Day 6

Sample 1-Sample 2	Test Statistic	Std. Error	Std. Test Statistic	Sig.
LDPE-LAP	-3,333	2,198	-1,516	,129
LDPE-MT	-5,667	2,198	-2,578	,010
LAP-MT	2,333	2,198	1,061	,289

Each row tests the null hypothesis that the Sample 1 and Sample 2 distributions are the same.

Asymptotic significances (2-sided tests) are displayed. The significance level is ,050.

Day 8

Sample 1-Sample 2	Test Statistic	Std. Error	Std. Test Statistic	Sig.
LDPE-MT	-4,167	2,227	-1,871	,061
LDPE-LAP	-4,833	2,227	-2,171	,030
MT-LAP	-,667	2,227	-,299	,765

Each row tests the null hypothesis that the Sample 1 and Sample 2 distributions are the same.

Asymptotic significances (2-sided tests) are displayed. The significance level is ,050.

Day 10

Sample 1-Sample 2	Test Statistic	Std. Error	Std. Test Statistic	Sig.
LDPE-LAP	-1,500	2,131	-,704	,482
LDPE-MT	-4,500	2,131	-2,112	,035
LAP-MT	3,000	2,131	1,408	,159

Each row tests the null hypothesis that the Sample 1 and Sample 2 distributions are the same.

Asymptotic significances (2-sided tests) are displayed. The significance level is ,050.

pH across days

Supplementary Table 2 pH across days

Hypothesis Test Summary

	Null Hypothesis	Test	Sig.[a,b]	Decision
1	The distribution of LDPE is the same across categories of Days_pH.	Independent-Samples Kruskal-Wallis Test	,006	Reject the null hypothesis.
2	The distribution of MT is the same across categories of Days_pH.	Independent-Samples Kruskal-Wallis Test	,006	Reject the null hypothesis.
3	The distribution of LAP is the same across categories of Days_pH.	Independent-Samples Kruskal-Wallis Test	,013	Reject the null hypothesis.

a. The significance level is ,050.

b. Asymptotic significance is displayed.

LDPE across pH

Sample 1-Sample 2	Test Statistic	Std. Error	Std. Test Statistic	Sig.
Day 10-Day 8	3,000	4,350	,690	,490
Day 10-Day 6	6,000	4,350	1,379	,168
Day 10-Day 4	9,667	4,350	2,222	,026
Day 10-Day 2	11,333	4,350	2,605	,009
Day 10-Day 0	15,000	4,350	3,448	<,001
Day 8-Day 6	3,000	4,350	,690	,490
Day 8-Day 4	6,667	4,350	1,533	,125
Day 8-Day 2	8,333	4,350	1,916	,055
Day 8-Day 0	12,000	4,350	2,759	,006
Day 6-Day 4	3,667	4,350	,843	,399
Day 6-Day 2	5,333	4,350	1,226	,220
Day 6-Day 0	9,000	4,350	2,069	,039
Day 4-Day 2	1,667	4,350	,383	,702
Day 4-Day 0	5,333	4,350	1,226	,220
Day 2-Day 0	3,667	4,350	,843	,399

MT across Days pH

Sample 1-Sample 2	Test Statistic	Std. Error	Std. Test Statistic	Sig.
Day 10-Day 4	2,333	4,345	,537	,591
Day 10-Day 8	6,167	4,345	1,419	,156

Day 10-Day 2	8,167	4,345	1,879	,060
Day 10-Day 6	11,667	4,345	2,685	,007
Day 10-Day 0	14,667	4,345	3,375	<,001
Day 4-Day 8	-3,833	4,345	-,882	,378
Day 4-Day 2	5,833	4,345	1,342	,179
Day 4-Day 6	-9,333	4,345	-2,148	,032
Day 4-Day 0	12,333	4,345	2,838	,005
Day 8-Day 2	2,000	4,345	,460	,645
Day 8-Day 6	5,500	4,345	1,266	,206
Day 8-Day 0	8,500	4,345	1,956	,050
Day 2-Day 6	-3,500	4,345	-,805	,421
Day 2-Day 0	6,500	4,345	1,496	,135
Day 6-Day 0	3,000	4,345	,690	,490

LAP across Days pH

Sample 1-Sample 2	Test Statistic	Std. Error	Std. Test Statistic	Sig.
Day 10-Day 4	4,667	4,323	1,080	,280
Day 10-Day 8	6,500	4,323	1,504	,133
Day 10-Day 2	7,667	4,323	1,774	,076
Day 10-Day 6	11,167	4,323	2,583	,010
Day 10-Day 0	15,000	4,323	3,470	<,001
Day 4-Day 8	-1,833	4,323	-,424	,671

Day 4-Day 2	3,000	4,323	,694	,488
Day 4-Day 6	-6,500	4,323	-1,504	,133
Day 4-Day 0	10,333	4,323	2,390	,017
Day 8-Day 2	1,167	4,323	,270	,787
Day 8-Day 6	4,667	4,323	1,080	,280
Day 8-Day 0	8,500	4,323	1,966	,049
Day 2-Day 6	-3,500	4,323	-,810	,418
Day 2-Day 0	7,333	4,323	1,696	,090
Day 6-Day 0	3,833	4,323	,887	,375

Lab*

Lab* across samples

Supplementary Table 3 Lab across samples*

Hypothesis Test Summary

	Null Hypothesis	Test	Sig.[a,b]	Decision
1	The distribution of L_Day_0 is the same across categories of Lab_Samples.	Independent-Samples Kruskal-Wallis Test	1,000	Retain the null hyp
2	The distribution of L_Day_2 is the same across categories of Lab_Samples.	Independent-Samples Kruskal-Wallis Test	<,001	Reject the null hyp
3	The distribution of L_Day_4 is the same across categories of Lab_Samples.	Independent-Samples Kruskal-Wallis Test	<,001	Reject the null hyp
4	The distribution of L_Day_6 is the same across categories of Lab_Samples.	Independent-Samples Kruskal-Wallis Test	<,001	Reject the null hyp

5	The distribution of L_Day_8 is the same across categories of Lab_Samples.	Independent-Samples Kruskal-Wallis Test	<,001	Reject the null hyp
6	The distribution of L_Day_10 is the same across categories of Lab_Samples.	Independent-Samples Kruskal-Wallis Test	<,001	Reject the null hyp
7	The distribution of a_Day_0 is the same across categories of Lab_Samples.	Independent-Samples Kruskal-Wallis Test	1,000	Retain the null hyp
8	The distribution of a_Day_2 is the same across categories of Lab_Samples.	Independent-Samples Kruskal-Wallis Test	<,001	Reject the null hyp
9	The distribution of a_Day_4 is the same across categories of Lab_Samples.	Independent-Samples Kruskal-Wallis Test	<,001	Reject the null hyp
10	The distribution of a_Day_6 is the same across categories of Lab_Samples.	Independent-Samples Kruskal-Wallis Test	,239	Retain the null hyp
11	The distribution of a_Day_8 is the same across categories of Lab_Samples.	Independent-Samples Kruskal-Wallis Test	<,001	Reject the null hyp
12	The distribution of a_Day_10 is the same across categories of Lab_Samples.	Independent-Samples Kruskal-Wallis Test	<,001	Reject the null hyp
13	The distribution of b_Day_0 is the same across categories of Lab_Samples.	Independent-Samples Kruskal-Wallis Test	1,000	Retain the null hyp
14	The distribution of b_Day_2 is the same across categories of Lab_Samples.	Independent-Samples Kruskal-Wallis Test	<,001	Reject the null hyp
15	The distribution of b_Day_4 is the same across categories of Lab_Samples.	Independent-Samples Kruskal-Wallis Test	,047	Reject the null hyp
16	The distribution of b_Day_6 is the same across categories of Lab_Samples.	Independent-Samples Kruskal-Wallis Test	,024	Reject the null hyp

17	The distribution of b_Day_8 is the same across categories of Lab_Samples.	Independent-Samples Kruskal-Wallis Test	<,001	Reject the null hyp
18	The distribution of b_Day_10 is the same across categories of Lab_Samples.	Independent-Samples Kruskal-Wallis Test	<,001	Reject the null hyp

a. The significance level is ,050.

b. Asymptotic significance is displayed.

L* parameter results

Day 0

Pairwise Comparisons of Lab_Samples

Sample 1-Sample 2	Test Statistic	Std. Error	Std. Test Statistic	Sig.	Adj. Sig.[a]
LDPE-MT	,000	3,511	,000	1,000	1,000
LDPE-LAP	,000	3,511	,000	1,000	1,000
MT-LAP	,000	3,511	,000	1,000	1,000

Each row tests the null hypothesis that the Sample 1 and Sample 2 distributions are the same.

Asymptotic significances (2-sided tests) are displayed. The significance level is ,050.

a. Significance values have been adjusted by the Bonferroni correction for multiple tests.

Day 2

Pairwise Comparisons of Lab_Samples

Sample 1-Sample 2	Test Statistic	Std. Error	Std. Test Statistic	Sig.	Adj. Sig.[a]
LDPE-MT	-9,562	3,533	-2,706	,007	,020
LDPE-LAP	-14,437	3,533	-4,086	<,001	,000
MT-LAP	-4,875	3,533	-1,380	,168	,503

Each row tests the null hypothesis that the Sample 1 and Sample 2 distributions are the same.

Asymptotic significances (2-sided tests) are displayed. The significance level is ,050.

a. Significance values have been adjusted by the Bonferroni correction for multiple tests.

Day 4

Pairwise Comparisons of Lab_Samples

Sample 1-Sample 2	Test Statistic	Std. Error	Std. Test Statistic	Sig.	Adj. Sig.[a]
LDPE-LAP	-11,437	3,532	-3,239	,001	,004
LDPE-MT	-12,562	3,532	-3,557	<,001	,001
LAP-MT	1,125	3,532	,319	,750	1,000

Each row tests the null hypothesis that the Sample 1 and Sample 2 distributions are the same.

Asymptotic significances (2-sided tests) are displayed. The significance level is ,050.

a. Significance values have been adjusted by the Bonferroni correction for multiple tests.

Day 6

Pairwise Comparisons of Lab_Samples

Sample 1-Sample 2	Test Statistic	Std. Error	Std. Test Statistic	Sig.	Adj. Sig.[a]
LDPE-LAP	-9,375	3,532	-2,654	,008	,024
LDPE-MT	-14,625	3,532	-4,140	<,001	,000
LAP-MT	5,250	3,532	1,486	,137	,412

Each row tests the null hypothesis that the Sample 1 and Sample 2 distributions are the same.

Asymptotic significances (2-sided tests) are displayed. The significance level is ,050.

a. Significance values have been adjusted by the Bonferroni correction for multiple tests.

Day 8

Pairwise Comparisons of Lab_Samples

Sample 1-Sample 2	Test Statistic	Std. Error	Std. Test Statistic	Sig.	Adj. Sig.[a]
LDPE-LAP	-8,250	3,532	-2,335	,020	,059
LDPE-MT	-15,750	3,532	-4,459	<,001	,000
LAP-MT	7,500	3,532	2,123	,034	,101

Each row tests the null hypothesis that the Sample 1 and Sample 2 distributions are the same.

Asymptotic significances (2-sided tests) are displayed. The significance level is ,050.

a. Significance values have been adjusted by the Bonferroni correction for multiple tests.

Day 10

Pairwise Comparisons of Lab_Samples

Sample 1-Sample 2	Test Statistic	Std. Error	Std. Test Statistic	Sig.	Adj. Sig.[a]
LDPE-LAP	-8,000	3,532	-2,265	,024	,071
LDPE-MT	-16,000	3,532	-4,529	<,001	,000
LAP-MT	8,000	3,532	2,265	,024	,071

Each row tests the null hypothesis that the Sample 1 and Sample 2 distributions are the same.

Asymptotic significances (2-sided tests) are displayed. The significance level is ,050.

a. Significance values have been adjusted by the Bonferroni correction for multiple tests.

a* parameter

Day 0

Pairwise Comparisons of Lab_Samples

Sample 1-Sample 2	Test Statistic	Std. Error	Std. Test Statistic	Sig.	Adj. Sig.[a]
LDPE-MT	,000	3,511	,000	1,000	1,000
LDPE-LAP	,000	3,511	,000	1,000	1,000
MT-LAP	,000	3,511	,000	1,000	1,000

Each row tests the null hypothesis that the Sample 1 and Sample 2 distributions are the same.

Asymptotic significances (2-sided tests) are displayed. The significance level is ,050.

a. Significance values have been adjusted by the Bonferroni correction for multiple tests.

Day 2

Pairwise Comparisons of Lab_Samples

Sample 1-Sample 2	Test Statistic	Std. Error	Std. Test Statistic	Sig.	Adj. Sig.[a]
LDPE-LAP	-10,000	3,527	-2,835	,005	,014
LDPE-MT	-14,000	3,527	-3,969	<,001	,000
LAP-MT	4,000	3,527	1,134	,257	,770

Each row tests the null hypothesis that the Sample 1 and Sample 2 distributions are the same.

Asymptotic significances (2-sided tests) are displayed. The significance level is ,050.

a. Significance values have been adjusted by the Bonferroni correction for multiple tests.

Day 4

Pairwise Comparisons of Lab_Samples

Sample 1-Sample 2	Test Statistic	Std. Error	Std. Test Statistic	Sig.	Adj. Sig.[a]
LDPE-MT	-10,312	3,530	-2,921	,003	,010
LDPE-LAP	-13,687	3,530	-3,877	<,001	,000
MT-LAP	-3,375	3,530	-,956	,339	1,000

Each row tests the null hypothesis that the Sample 1 and Sample 2 distributions are the same.

Asymptotic significances (2-sided tests) are displayed. The significance level is ,050.

a. Significance values have been adjusted by the Bonferroni correction for multiple tests.

Day 6

Pairwise Comparisons of Lab_Samples

Sample 1-Sample 2	Test Statistic	Std. Error	Std. Test Statistic	Sig.	Adj. Sig.[a]
LDPE-MT	-3,875	3,533	-1,097	,273	,818
LDPE-LAP	-5,875	3,533	-1,663	,096	,289
MT-LAP	-2,000	3,533	-,566	,571	1,000

Each row tests the null hypothesis that the Sample 1 and Sample 2 distributions are the same.

Asymptotic significances (2-sided tests) are displayed. The significance level is ,050.

a. Significance values have been adjusted by the Bonferroni correction for multiple tests.

Day 8

Pairwise Comparisons of Lab_Samples

Sample 1-Sample 2	Test Statistic	Std. Error	Std. Test Statistic	Sig.	Adj. Sig.[a]
LDPE-LAP	-11,375	3,533	-3,219	,001	,004
LDPE-MT	-11,875	3,533	-3,361	<,001	,002
LAP-MT	,500	3,533	,142	,887	1,000

Each row tests the null hypothesis that the Sample 1 and Sample 2 distributions are the same.

Asymptotic significances (2-sided tests) are displayed. The significance level is ,050.

a. Significance values have been adjusted by the Bonferroni correction for multiple tests.

Day 10

Pairwise Comparisons of Lab_Samples

Sample 1-Sample 2	Test Statistic	Std. Error	Std. Test Statistic	Sig.	Adj. Sig.[a]
LDPE-LAP	-10,875	3,533	-3,078	,002	,006
LDPE-MT	-13,125	3,533	-3,715	<,001	,001
LAP-MT	2,250	3,533	,637	,524	1,000

Each row tests the null hypothesis that the Sample 1 and Sample 2 distributions are the same.

Asymptotic significances (2-sided tests) are displayed. The significance level is ,050.

a. Significance values have been adjusted by the Bonferroni correction for multiple tests.

b* parameter

Day 0

Pairwise Comparisons of Lab_Samples

Sample 1-Sample 2	Test Statistic	Std. Error	Std. Test Statistic	Sig.	Adj. Sig.[a]
LDPE-MT	,000	3,511	,000	1,000	1,000
LDPE-LAP	,000	3,511	,000	1,000	1,000
MT-LAP	,000	3,511	,000	1,000	1,000

Each row tests the null hypothesis that the Sample 1 and Sample 2 distributions are the same.

Asymptotic significances (2-sided tests) are displayed. The significance level is ,050.

a. Significance values have been adjusted by the Bonferroni correction for multiple tests.

Day 2

Pairwise Comparisons of Lab_Samples

Sample 1-Sample 2	Test Statistic	Std. Error	Std. Test Statistic	Sig.	Adj. Sig.[a]
LAP-MT	1,250	3,523	,355	,723	1,000
LAP-LDPE	12,250	3,523	3,477	<,001	,002
MT-LDPE	11,000	3,523	3,122	,002	,005

Each row tests the null hypothesis that the Sample 1 and Sample 2 distributions are the same.

Asymptotic significances (2-sided tests) are displayed. The significance level is ,050.

a. Significance values have been adjusted by the Bonferroni correction for multiple tests.

Day 4

Pairwise Comparisons of Lab_Samples

Sample 1-Sample 2	Test Statistic	Std. Error	Std. Test Statistic	Sig.	Adj. Sig.[a]
LAP-MT	,875	3,524	,248	,804	1,000
LAP-LDPE	7,938	3,524	2,252	,024	,073
MT-LDPE	7,063	3,524	2,004	,045	,135

Each row tests the null hypothesis that the Sample 1 and Sample 2 distributions are the same.

Asymptotic significances (2-sided tests) are displayed. The significance level is ,050.

a. Significance values have been adjusted by the Bonferroni correction for multiple tests.

Day 6

Pairwise Comparisons of Lab_Samples

Sample 1-Sample 2	Test Statistic	Std. Error	Std. Test Statistic	Sig.	Adj. Sig.[a]
MT-LAP	-1,500	3,532	-,425	,671	1,000
MT-LDPE	9,000	3,532	2,548	,011	,032
LAP-LDPE	7,500	3,532	2,124	,034	,101

Each row tests the null hypothesis that the Sample 1 and Sample 2 distributions are the same.

Asymptotic significances (2-sided tests) are displayed. The significance level is ,050.

a. Significance values have been adjusted by the Bonferroni correction for multiple tests.

Day 8

Pairwise Comparisons of Lab_Samples

Sample 1-Sample 2	Test Statistic	Std. Error	Std. Test Statistic	Sig.	Adj. Sig.[a]
MT-LAP	-2,750	3,532	-,779	,436	1,000
MT-LDPE	13,000	3,532	3,681	<,001	,001

LAP-LDPE	10,250	3,532	2,902	,004	,011

Each row tests the null hypothesis that the Sample 1 and Sample 2 distributions are the same.

Asymptotic significances (2-sided tests) are displayed. The significance level is ,050.

a. Significance values have been adjusted by the Bonferroni correction for multiple tests.

Day 10

Pairwise Comparisons of Lab_Samples

Sample 1-Sample 2	Test Statistic	Std. Error	Std. Test Statistic	Sig.	Adj. Sig.[a]
MT-LAP	-5,250	3,532	-1,487	,137	,411
MT-LDPE	14,625	3,532	4,141	<,001	,000
LAP-LDPE	9,375	3,532	2,655	,008	,024

Each row tests the null hypothesis that the Sample 1 and Sample 2 distributions are the same.

Asymptotic significances (2-sided tests) are displayed. The significance level is ,050.

a. Significance values have been adjusted by the Bonferroni correction for multiple tests.

Lab* across days

Supplementary Table 4 Lab across days*

Hypothesis Test Summary

Null Hypothesis	Test	Sig.[a,b]	Decision

1	The distribution of L_LDPE is the same across categories of Lab_Days.	Independent-Samples Kruskal-Wallis Test	<,001	Reject the null hypothesis.
2	The distribution of L_MT is the same across categories of Lab_Days.	Independent-Samples Kruskal-Wallis Test	<,001	Reject the null hypothesis.
3	The distribution of L_LAP is the same across categories of Lab_Days.	Independent-Samples Kruskal-Wallis Test	<,001	Reject the null hypothesis.
4	The distribution of a_LDPE is the same across categories of Lab_Days.	Independent-Samples Kruskal-Wallis Test	<,001	Reject the null hypothesis.
5	The distribution of a_MT is the same across categories of Lab_Days.	Independent-Samples Kruskal-Wallis Test	<,001	Reject the null hypothesis.
6	The distribution of a_LAP is the same across categories of Lab_Days.	Independent-Samples Kruskal-Wallis Test	<,001	Reject the null hypothesis.
7	The distribution of b_LDPE is the same across categories of Lab_Days.	Independent-Samples Kruskal-Wallis Test	<,001	Reject the null hypothesis.
8	The distribution of b_MT is the same across categories of Lab_Days.	Independent-Samples Kruskal-Wallis Test	<,001	Reject the null hypothesis.
9	The distribution of b_LAP is the same across categories of Lab_Days.	Independent-Samples Kruskal-Wallis Test	<,001	Reject the null hypothesis.

a. The significance level is ,050.

b. Asymptotic significance is displayed.

L_LDPE

Pairwise Comparisons of Lab_Days

Sample 1-Sample 2	Test Statistic	Std. Error	Std. Test Statistic	Sig.	Adj. Sig.[a]
Day 10-Day 8	8,000	6,998	1,143	,253	1,000
Day 10-Day 6	16,000	6,998	2,286	,022	,334
Day 10-Day 4	24,000	6,998	3,429	<,001	,009
Day 10-Day 2	32,000	6,998	4,572	<,001	,000
Day 10-Day 0	40,000	6,998	5,716	<,001	,000
Day 8-Day 6	8,000	6,998	1,143	,253	1,000
Day 8-Day 4	16,000	6,998	2,286	,022	,334
Day 8-Day 2	24,000	6,998	3,429	<,001	,009
Day 8-Day 0	32,000	6,998	4,572	<,001	,000
Day 6-Day 4	8,000	6,998	1,143	,253	1,000
Day 6-Day 2	16,000	6,998	2,286	,022	,334
Day 6-Day 0	24,000	6,998	3,429	<,001	,009
Day 4-Day 2	8,000	6,998	1,143	,253	1,000
Day 4-Day 0	16,000	6,998	2,286	,022	,334
Day 2-Day 0	8,000	6,998	1,143	,253	1,000

Each row tests the null hypothesis that the Sample 1 and Sample 2 distributions are the same.

Asymptotic significances (2-sided tests) are displayed. The significance level is ,050.

a. Significance values have been adjusted by the Bonferroni correction for multiple tests.

L_MT

Pairwise Comparisons of Lab_Days

Sample 1-Sample 2	Test Statistic	Std. Error	Std. Test Statistic	Sig.	Adj. Sig.[a]
Day 10-Day 8	8,000	6,999	1,143	,253	1,000

Day 10-Day 6	16,000	6,999	2,286	,022	,334
Day 10-Day 4	24,000	6,999	3,429	<,001	,009
Day 10-Day 2	32,500	6,999	4,643	<,001	,000
Day 10-Day 0	39,500	6,999	5,644	<,001	,000
Day 8-Day 6	8,000	6,999	1,143	,253	1,000
Day 8-Day 4	16,000	6,999	2,286	,022	,334
Day 8-Day 2	24,500	6,999	3,500	<,001	,007
Day 8-Day 0	31,500	6,999	4,501	<,001	,000
Day 6-Day 4	8,000	6,999	1,143	,253	1,000
Day 6-Day 2	16,500	6,999	2,357	,018	,276
Day 6-Day 0	23,500	6,999	3,358	<,001	,012
Day 4-Day 2	8,500	6,999	1,214	,225	1,000
Day 4-Day 0	15,500	6,999	2,215	,027	,402
Day 2-Day 0	7,000	6,999	1,000	,317	1,000

Each row tests the null hypothesis that the Sample 1 and Sample 2 distributions are the same.

Asymptotic significances (2-sided tests) are displayed. The significance level is ,050.

a. Significance values have been adjusted by the Bonferroni correction for multiple tests.

L_Lap

Pairwise Comparisons of Lab_Days

Sample 1-Sample 2	Test Statistic	Std. Error	Std. Test Statistic	Sig.	Adj. Sig.[a]
Day 10-Day 8	8,000	6,998	1,143	,253	1,000
Day 10-Day 6	16,000	6,998	2,286	,022	,334
Day 10-Day 4	24,000	6,998	3,429	<,001	,009
Day 10-Day 2	34,625	6,998	4,948	<,001	,000

Day 10-Day 0	37,375	6,998	5,340	<,001	,000
Day 8-Day 6	8,000	6,998	1,143	,253	1,000
Day 8-Day 4	16,000	6,998	2,286	,022	,334
Day 8-Day 2	26,625	6,998	3,804	<,001	,002
Day 8-Day 0	29,375	6,998	4,197	<,001	,000
Day 6-Day 4	8,000	6,998	1,143	,253	1,000
Day 6-Day 2	18,625	6,998	2,661	,008	,117
Day 6-Day 0	21,375	6,998	3,054	,002	,034
Day 4-Day 2	10,625	6,998	1,518	,129	1,000
Day 4-Day 0	13,375	6,998	1,911	,056	,840
Day 2-Day 0	2,750	6,998	,393	,694	1,000

Each row tests the null hypothesis that the Sample 1 and Sample 2 distributions are the same.

Asymptotic significances (2-sided tests) are displayed. The significance level is ,050.

a. Significance values have been adjusted by the Bonferroni correction for multiple tests.

a_LDPE

Pairwise Comparisons of Lab_Days

Sample 1-Sample 2	Test Statistic	Std. Error	Std. Test Statistic	Sig.	Adj. Sig.[a]
Day 10-Day 4	2,375	6,998	,339	,734	1,000
Day 10-Day 8	14,000	6,998	2,001	,045	,681
Day 10-Day 2	19,875	6,998	2,840	,005	,068
Day 10-Day 6	26,875	6,998	3,841	<,001	,002
Day 10-Day 0	36,625	6,998	5,234	<,001	,000
Day 4-Day 8	-11,625	6,998	-1,661	,097	1,000
Day 4-Day 2	17,500	6,998	2,501	,012	,186

Day 4-Day 6	-24,500	6,998	-3,501	<,001	,007
Day 4-Day 0	34,250	6,998	4,895	<,001	,000
Day 8-Day 2	5,875	6,998	,840	,401	1,000
Day 8-Day 6	12,875	6,998	1,840	,066	,987
Day 8-Day 0	22,625	6,998	3,233	,001	,018
Day 2-Day 6	-7,000	6,998	-1,000	,317	1,000
Day 2-Day 0	16,750	6,998	2,394	,017	,250
Day 6-Day 0	9,750	6,998	1,393	,164	1,000

Each row tests the null hypothesis that the Sample 1 and Sample 2 distributions are the same.

Asymptotic significances (2-sided tests) are displayed. The significance level is ,050.

a. Significance values have been adjusted by the Bonferroni correction for multiple tests.

a_MT

Pairwise Comparisons of Lab_Days

Sample 1-Sample 2	Test Statistic	Std. Error	Std. Test Statistic	Sig.	Adj. Sig.[a]
Day 10-Day 8	5,375	6,999	,768	,442	1,000
Day 10-Day 6	10,625	6,999	1,518	,129	1,000
Day 10-Day 4	16,000	6,999	2,286	,022	,334
Day 10-Day 2	29,625	6,999	4,233	<,001	,000
Day 10-Day 0	34,375	6,999	4,912	<,001	,000
Day 8-Day 6	5,250	6,999	,750	,453	1,000
Day 8-Day 4	10,625	6,999	1,518	,129	1,000
Day 8-Day 2	24,250	6,999	3,465	<,001	,008
Day 8-Day 0	29,000	6,999	4,144	<,001	,001
Day 6-Day 4	5,375	6,999	,768	,442	1,000

Day 6-Day 2	19,000	6,999	2,715	,007	,099
Day 6-Day 0	23,750	6,999	3,394	<,001	,010
Day 4-Day 2	13,625	6,999	1,947	,052	,773
Day 4-Day 0	18,375	6,999	2,625	,009	,130
Day 2-Day 0	4,750	6,999	,679	,497	1,000

Each row tests the null hypothesis that the Sample 1 and Sample 2 distributions are the same.

Asymptotic significances (2-sided tests) are displayed. The significance level is ,050.

a. Significance values have been adjusted by the Bonferroni correction for multiple tests.

Pairwise Comparisons of Lab_Days

Sample 1-Sample 2	Test Statistic	Std. Error	Std. Test Statistic	Sig.	Adj. Sig.[a]
Day 10-Day 8	7,500	6,999	1,072	,284	1,000
Day 10-Day 6	15,500	6,999	2,215	,027	,402
Day 10-Day 4	23,000	6,999	3,286	,001	,015
Day 10-Day 2	32,250	6,999	4,608	<,001	,000
Day 10-Day 0	38,750	6,999	5,536	<,001	,000
Day 8-Day 6	8,000	6,999	1,143	,253	1,000
Day 8-Day 4	15,500	6,999	2,215	,027	,402
Day 8-Day 2	24,750	6,999	3,536	<,001	,006
Day 8-Day 0	31,250	6,999	4,465	<,001	,000
Day 6-Day 4	7,500	6,999	1,072	,284	1,000
Day 6-Day 2	16,750	6,999	2,393	,017	,251
Day 6-Day 0	23,250	6,999	3,322	<,001	,013
Day 4-Day 2	9,250	6,999	1,322	,186	1,000

Day 4-Day 0	15,750	6,999	2,250	,024	,366
Day 2-Day 0	6,500	6,999	,929	,353	1,000

Each row tests the null hypothesis that the Sample 1 and Sample 2 distributions are the same.

Asymptotic significances (2-sided tests) are displayed. The significance level is ,050.

a. Significance values have been adjusted by the Bonferroni correction for multiple tests.

b_ LDPE

Pairwise Comparisons of Lab_Days

Sample 1-Sample 2	Test Statistic	Std. Error	Std. Test Statistic	Sig.	Adj. Sig.[a]
Day 0-Day 2	-11,000	6,999	-1,572	,116	1,000
Day 0-Day 4	-15,750	6,999	-2,250	,024	,366
Day 0-Day 6	-22,000	6,999	-3,143	,002	,025
Day 0-Day 8	-32,000	6,999	-4,572	<,001	,000
Day 0-Day 10	-39,250	6,999	-5,608	<,001	,000
Day 2-Day 4	-4,750	6,999	-,679	,497	1,000
Day 2-Day 6	-11,000	6,999	-1,572	,116	1,000
Day 2-Day 8	-21,000	6,999	-3,000	,003	,040
Day 2-Day 10	-28,250	6,999	-4,036	<,001	,001
Day 4-Day 6	-6,250	6,999	-,893	,372	1,000
Day 4-Day 8	-16,250	6,999	-2,322	,020	,304
Day 4-Day 10	-23,500	6,999	-3,358	<,001	,012
Day 6-Day 8	-10,000	6,999	-1,429	,153	1,000
Day 6-Day 10	-17,250	6,999	-2,465	,014	,206
Day 8-Day 10	-7,250	6,999	-1,036	,300	1,000

Each row tests the null hypothesis that the Sample 1 and Sample 2 distributions are the same.

Asymptotic significances (2-sided tests) are displayed. The significance level is ,050.

a. Significance values have been adjusted by the Bonferroni correction for multiple tests.

b_ MT

Pairwise Comparisons of Lab_Days

Sample 1-Sample 2	Test Statistic	Std. Error	Std. Test Statistic	Sig.	Adj. Sig.[a]
Day 0-Day 2	-3,000	6,999	-,429	,668	1,000
Day 0-Day 4	-15,625	6,999	-2,232	,026	,384
Day 0-Day 6	-21,875	6,999	-3,125	,002	,027
Day 0-Day 8	-29,125	6,999	-4,161	<,001	,000
Day 0-Day 10	-35,375	6,999	-5,054	<,001	,000
Day 2-Day 4	-12,625	6,999	-1,804	,071	1,000
Day 2-Day 6	-18,875	6,999	-2,697	,007	,105
Day 2-Day 8	-26,125	6,999	-3,732	<,001	,003
Day 2-Day 10	-32,375	6,999	-4,625	<,001	,000
Day 4-Day 6	-6,250	6,999	-,893	,372	1,000
Day 4-Day 8	-13,500	6,999	-1,929	,054	,806
Day 4-Day 10	-19,750	6,999	-2,822	,005	,072
Day 6-Day 8	-7,250	6,999	-1,036	,300	1,000
Day 6-Day 10	-13,500	6,999	-1,929	,054	,806
Day 8-Day 10	-6,250	6,999	-,893	,372	1,000

Each row tests the null hypothesis that the Sample 1 and Sample 2 distributions are the same.

Asymptotic significances (2-sided tests) are displayed. The significance level is ,050.

a. Significance values have been adjusted by the Bonferroni correction for multiple tests.

b_LAP

Pairwise Comparisons of Lab_Days

Sample 1-Sample 2	Test Statistic	Std. Error	Std. Test Statistic	Sig.	Adj. Sig.[a]
Day 2-Day 0	,313	6,995	,045	,964	1,000
Day 2-Day 4	-13,187	6,995	-1,885	,059	,891
Day 2-Day 6	-20,250	6,995	-2,895	,004	,057
Day 2-Day 8	-28,000	6,995	-4,003	<,001	,001
Day 2-Day 10	-35,000	6,995	-5,003	<,001	,000
Day 0-Day 4	-12,875	6,995	-1,840	,066	,985
Day 0-Day 6	-19,937	6,995	-2,850	,004	,066
Day 0-Day 8	-27,687	6,995	-3,958	<,001	,001
Day 0-Day 10	-34,687	6,995	-4,959	<,001	,000
Day 4-Day 6	-7,062	6,995	-1,010	,313	1,000
Day 4-Day 8	-14,812	6,995	-2,117	,034	,513
Day 4-Day 10	-21,812	6,995	-3,118	,002	,027
Day 6-Day 8	-7,750	6,995	-1,108	,268	1,000
Day 6-Day 10	-14,750	6,995	-2,109	,035	,525
Day 8-Day 10	-7,000	6,995	-1,001	,317	1,000

Each row tests the null hypothesis that the Sample 1 and Sample 2 distributions are the same.

Asymptotic significances (2-sided tests) are displayed. The significance level is ,050.

a. Significance values have been adjusted by the Bonferroni correction for multiple tests.

Sensory Analysis

Sensory across samples

Supplementary Table 5 Sensory analysis across samples

Hypothesis Test Summary

	Null Hypothesis	Test	Sig.[a,b]	Decision
1	The distribution of Color_day0 is the same across categories of Sensory_samples.	Independent-Samples Kruskal-Wallis Test	1,000	Retain the null hypothesis.
2	The distribution of Color_day2 is the same across categories of Sensory_samples.	Independent-Samples Kruskal-Wallis Test	,045	Reject the null hypothesis.
3	The distribution of Color_day4 is the same across categories of Sensory_samples.	Independent-Samples Kruskal-Wallis Test	,111	Retain the null hypothesis.
4	The distribution of Color_day6 is the same across categories of Sensory_samples.	Independent-Samples Kruskal-Wallis Test	,358	Retain the null hypothesis.
5	The distribution of Color_day8 is the same across categories of Sensory_samples.	Independent-Samples Kruskal-Wallis Test	,230	Retain the null hypothesis.
6	The distribution of Color_day10 is the same across categories of Sensory_samples.	Independent-Samples Kruskal-Wallis Test	,214	Retain the null hypothesis.
7	The distribution of Aroma_day0 is the same across categories of Sensory_samples.	Independent-Samples Kruskal-Wallis Test	1,000	Retain the null hypothesis.
8	The distribution of Aroma_day2 is the same across categories of Sensory_samples.	Independent-Samples Kruskal-Wallis Test	,059	Retain the null hypothesis.
9	The distribution of Aroma_day4 is the same across categories of Sensory_samples.	Independent-Samples Kruskal-Wallis Test	,050	Reject the null hypothesis.

10	The distribution of Aroma_day6 is the same across categories of Sensory_samples.	Independent-Samples Kruskal-Wallis Test	,748	Retain the null hypothesis.
11	The distribution of Aroma_day8 is the same across categories of Sensory_samples.	Independent-Samples Kruskal-Wallis Test	,064	Retain the null hypothesis.
12	The distribution of Aroma_day10 is the same across categories of Sensory_samples.	Independent-Samples Kruskal-Wallis Test	,043	Reject the null hypothesis.
13	The distribution of Texture_day0 is the same across categories of Sensory_samples.	Independent-Samples Kruskal-Wallis Test	1,000	Retain the null hypothesis.
14	The distribution of Texture_day2 is the same across categories of Sensory_samples.	Independent-Samples Kruskal-Wallis Test	,063	Retain the null hypothesis.
15	The distribution of Texture_day4 is the same across categories of Sensory_samples.	Independent-Samples Kruskal-Wallis Test	,229	Retain the null hypothesis.
16	The distribution of Texture_day6 is the same across categories of Sensory_samples.	Independent-Samples Kruskal-Wallis Test	,061	Retain the null hypothesis.
17	The distribution of Texture_day8 is the same across categories of Sensory_samples.	Independent-Samples Kruskal-Wallis Test	,541	Retain the null hypothesis.
18	The distribution of Texture_day10 is the same across categories of Sensory_samples.	Independent-Samples Kruskal-Wallis Test	,118	Retain the null hypothesis.

a. The significance level is ,050.

b. Asymptotic significance is displayed.

Color results per day

Day 2

Sample 1-Sample 2	Test Statistic	Std. Error	Std. Test Statistic	Sig.
LDPE-MT	-2,500	2,208	-1,132	,258
LDPE-LAP	-5,500	2,208	-2,491	,013
MT-LAP	-3,000	2,208	-1,359	,174

Day 4

Sample 1-Sample 2	Test Statistic	Std. Error	Std. Test Statistic	Sig.
MT-LAP	-1,167	2,141	-,545	,586
MT-LDPE	4,333	2,141	2,024	,043
LAP-LDPE	3,167	2,141	1,479	,139

Day 6

Sample 1-Sample 2	Test Statistic	Std. Error	Std. Test Statistic	Sig.
LAP-MT	,167	2,217	,075	,940
LAP-LDPE	2,833	2,217	1,278	,201
MT-LDPE	2,667	2,217	1,203	,229

Day 8

Sample 1-Sample 2	Test Statistic	Std. Error	Std. Test Statistic	Sig.
MT-LAP	-,500	2,208	-,226	,821
MT-LDPE	3,500	2,208	1,585	,113
LAP-LDPE	3,000	2,208	1,359	,174

Day 10

Sample 1-Sample 2	Test Statistic	Std. Error	Std. Test Statistic	Sig.
LDPE-MT	-2,167	2,189	-,990	,322
LDPE-LAP	-3,833	2,189	-1,751	,080
MT-LAP	-1,667	2,189	-,761	,446

Aroma results per day

Sample 1-Sample 2	Test Statistic	Std. Error	Std. Test Statistic	Sig.
LDPE-LAP	-4,000	2,227	-1,796	,072
LDPE-MT	-5,000	2,227	-2,245	,025
LAP-MT	1,000	2,227	,449	,653

Day 4

Sample 1-Sample 2	Test Statistic	Std. Error	Std. Test Statistic	Sig.
LDPE-MT	,000	2,121	,000	1,000
LDPE-LAP	-4,500	2,121	-2,121	,034
MT-LAP	-4,500	2,121	-2,121	,034

Day 6

Sample 1-Sample 2	Test Statistic	Std. Error	Std. Test Statistic	Sig.
LDPE-LAP	-,833	2,189	-,381	,703
LDPE-MT	-1,667	2,189	-,761	,446
LAP-MT	,833	2,189	,381	,703

Day 8

Sample 1-Sample 2	Test Statistic	Std. Error	Std. Test Statistic	Sig.
LDPE-MT	-4,500	2,217	-2,029	,042
LDPE-LAP	-4,500	2,217	-2,029	,042
MT-LAP	,000	2,217	,000	1,000

Day 10

Sample 1-Sample 2	Test Statistic	Std. Error	Std. Test Statistic	Sig.
LDPE-MT	-3,500	2,217	-1,578	,114
LDPE-LAP	-5,500	2,217	-2,480	,013
MT-LAP	-2,000	2,217	-,902	,367

Texture results per day

Day 2

Sample 1-Sample 2	Test Statistic	Std. Error	Std. Test Statistic	Sig.
LDPE-MT	-4,500	2,208	-2,038	,042
LDPE-LAP	-4,500	2,208	-2,038	,042
MT-LAP	,000	2,208	,000	1,000

Day 4

Sample 1-Sample 2	Test Statistic	Std. Error	Std. Test Statistic	Sig.
LDPE-MT	-2,333	2,160	-1,080	,280
LDPE-LAP	-3,667	2,160	-1,697	,090

MT-LAP	-1,333	2,160	-,617	,537

Day 6

Sample 1-Sample 2	Test Statistic	Std. Error	Std. Test Statistic	Sig.
MT-LDPE	3,500	2,170	1,613	,107
MT-LAP	-5,000	2,170	-2,304	,021
LDPE-LAP	-1,500	2,170	-,691	,489

Day 8

Sample 1-Sample 2	Test Statistic	Std. Error	Std. Test Statistic	Sig.
MT-LDPE	1,667	2,170	,768	,442
MT-LAP	-2,333	2,170	-1,075	,282
LDPE-LAP	-,667	2,170	-,307	,759

Day 10

Sample 1-Sample 2	Test Statistic	Std. Error	Std. Test Statistic	Sig.
LDPE-MT	-2,500	2,179	-1,147	,251
LDPE-LAP	-4,500	2,179	-2,065	,039
MT-LAP	-2,000	2,179	-,918	,359

Sensory across days

Supplementary Table 6 Results across days

Hypothesis Test Summary

	Null Hypothesis	Test	Sig.[a,b]	Decision
1	The distribution of LDPE_color_results is the same across categories of Sensory_days.	Independent-Samples Kruskal-Wallis Test	,007	Reject the null hypothesis.
2	The distribution of MT_color_results is the same across categories of Sensory_days.	Independent-Samples Kruskal-Wallis Test	,007	Reject the null hypothesis.
3	The distribution of LAP_color_results is the same across categories of Sensory_days.	Independent-Samples Kruskal-Wallis Test	,006	Reject the null hypothesis.
4	The distribution of LDPE_aroma_results is the same across categories of Sensory_days.	Independent-Samples Kruskal-Wallis Test	,006	Reject the null hypothesis.
5	The distribution of MT_aroma_results is the same across categories of Sensory_days.	Independent-Samples Kruskal-Wallis Test	,010	Reject the null hypothesis.
6	The distribution of LAP_aroma_results is the same across categories of Sensory_days.	Independent-Samples Kruskal-Wallis Test	,006	Reject the null hypothesis.
7	The distribution of LDPE_texture_results is the same across categories of Sensory_days.	Independent-Samples Kruskal-Wallis Test	,010	Reject the null hypothesis.
8	The distribution of MT_texture_results is the same across categories of Sensory_days.	Independent-Samples Kruskal-Wallis Test	,007	Reject the null hypothesis.
9	The distribution of LAP_texture_results is the same across categories of Sensory_days.	Independent-Samples Kruskal-Wallis Test	,009	Reject the null hypothesis.

a. The significance level is ,050.

b. Asymptotic significance is displayed.

LDPE – color

Sample 1-Sample 2	Test Statistic	Std. Error	Std. Test Statistic	Sig.
Day 10-Day 8	3,833	4,339	,884	,377
Day 10-Day 6	5,167	4,339	1,191	,234
Day 10-Day 2	10,333	4,339	2,382	,017
Day 10-Day 4	10,667	4,339	2,459	,014
Day 10-Day 0	15,000	4,339	3,457	<,001
Day 8-Day 6	1,333	4,339	,307	,759
Day 8-Day 2	6,500	4,339	1,498	,134
Day 8-Day 4	6,833	4,339	1,575	,115
Day 8-Day 0	11,167	4,339	2,574	,010
Day 6-Day 2	5,167	4,339	1,191	,234
Day 6-Day 4	5,500	4,339	1,268	,205
Day 6-Day 0	9,833	4,339	2,266	,023
Day 2-Day 4	-,333	4,339	-,077	,939
Day 2-Day 0	4,667	4,339	1,076	,282
Day 4-Day 0	4,333	4,339	,999	,318

MT – color

Sample 1-Sample 2	Test Statistic	Std. Error	Std. Test Statistic	Sig.
Day 10-Day 8	3,500	4,345	,805	,421
Day 10-Day 6	6,000	4,345	1,381	,167
Day 10-Day 4	8,500	4,345	1,956	,050
Day 10-Day 2	12,000	4,345	2,762	,006
Day 10-Day 0	15,000	4,345	3,452	<,001

Day 8-Day 6	2,500	4,345	,575	,565
Day 8-Day 4	5,000	4,345	1,151	,250
Day 8-Day 2	8,500	4,345	1,956	,050
Day 8-Day 0	11,500	4,345	2,646	,008
Day 6-Day 4	2,500	4,345	,575	,565
Day 6-Day 2	6,000	4,345	1,381	,167
Day 6-Day 0	9,000	4,345	2,071	,038
Day 4-Day 2	3,500	4,345	,805	,421
Day 4-Day 0	6,500	4,345	1,496	,135
Day 2-Day 0	3,000	4,345	,690	,490

LAP color

Sample 1-Sample 2	Test Statistic	Std. Error	Std. Test Statistic	Sig.
Day 10-Day 8	3,667	4,341	,845	,398
Day 10-Day 6	5,333	4,341	1,229	,219
Day 10-Day 4	9,000	4,341	2,073	,038
Day 10-Day 2	12,000	4,341	2,764	,006
Day 10-Day 0	15,000	4,341	3,456	<,001
Day 8-Day 6	1,667	4,341	,384	,701
Day 8-Day 4	5,333	4,341	1,229	,219
Day 8-Day 2	8,333	4,341	1,920	,055
Day 8-Day 0	11,333	4,341	2,611	,009
Day 6-Day 4	3,667	4,341	,845	,398
Day 6-Day 2	6,667	4,341	1,536	,125
Day 6-Day 0	9,667	4,341	2,227	,026
Day 4-Day 2	3,000	4,341	,691	,489

Day 4-Day 0	6,000	4,341	1,382	,167
Day 2-Day 0	3,000	4,341	,691	,489

LDPE aroma

Sample 1-Sample 2	Test Statistic	Std. Error	Std. Test Statistic	Sig.
Day 10-Day 8	3,000	4,343	,691	,490
Day 10-Day 6	7,167	4,343	1,650	,099
Day 10-Day 4	7,833	4,343	1,804	,071
Day 10-Day 2	12,000	4,343	2,763	,006
Day 10-Day 0	15,000	4,343	3,454	<,001
Day 8-Day 6	4,167	4,343	,959	,337
Day 8-Day 4	4,833	4,343	1,113	,266
Day 8-Day 2	9,000	4,343	2,072	,038
Day 8-Day 0	12,000	4,343	2,763	,006
Day 6-Day 4	,667	4,343	,153	,878
Day 6-Day 2	4,833	4,343	1,113	,266
Day 6-Day 0	7,833	4,343	1,804	,071
Day 4-Day 2	4,167	4,343	,959	,337
Day 4-Day 0	7,167	4,343	1,650	,099
Day 2-Day 0	3,000	4,343	,691	,490

MT aroma results

Sample 1-Sample 2	Test Statistic	Std. Error	Std. Test Statistic	Sig.
Day 10-Day 8	4,833	4,334	1,115	,265

Day 10-Day 4	6,000	4,334	1,384	,166
Day 10-Day 6	7,167	4,334	1,654	,098
Day 10-Day 2	12,000	4,334	2,769	,006
Day 10-Day 0	15,000	4,334	3,461	<,001
Day 8-Day 4	1,167	4,334	,269	,788
Day 8-Day 6	2,333	4,334	,538	,590
Day 8-Day 2	7,167	4,334	1,654	,098
Day 8-Day 0	10,167	4,334	2,346	,019
Day 4-Day 6	-1,167	4,334	-,269	,788
Day 4-Day 2	6,000	4,334	1,384	,166
Day 4-Day 0	9,000	4,334	2,077	,038
Day 6-Day 2	4,833	4,334	1,115	,265
Day 6-Day 0	7,833	4,334	1,807	,071
Day 2-Day 0	3,000	4,334	,692	,489

LAP aroma results

Sample 1-Sample 2	Test Statistic	Std. Error	Std. Test Statistic	Sig.
Day 10-Day 8	4,167	4,345	,959	,338
Day 10-Day 6	4,833	4,345	1,112	,266
Day 10-Day 4	9,000	4,345	2,071	,038
Day 10-Day 2	12,000	4,345	2,762	,006
Day 10-Day 0	15,000	4,345	3,452	<,001
Day 8-Day 6	,667	4,345	,153	,878
Day 8-Day 4	4,833	4,345	1,112	,266
Day 8-Day 2	7,833	4,345	1,803	,071
Day 8-Day 0	10,833	4,345	2,493	,013

Day 6-Day 4	4,167	4,345	,959	,338
Day 6-Day 2	7,167	4,345	1,649	,099
Day 6-Day 0	10,167	4,345	2,340	,019
Day 4-Day 2	3,000	4,345	,690	,490
Day 4-Day 0	6,000	4,345	1,381	,167
Day 2-Day 0	3,000	4,345	,690	,490

LDPE texture results

Sample 1-Sample 2	Test Statistic	Std. Error	Std. Test Statistic	Sig.
Day 10-Day 4	5,333	4,320	1,234	,217
Day 10-Day 8	6,000	4,320	1,389	,165
Day 10-Day 6	6,667	4,320	1,543	,123
Day 10-Day 2	12,000	4,320	2,777	,005
Day 10-Day 0	15,000	4,320	3,472	<,001
Day 4-Day 8	-,667	4,320	-,154	,877
Day 4-Day 6	-1,333	4,320	-,309	,758
Day 4-Day 2	6,667	4,320	1,543	,123
Day 4-Day 0	9,667	4,320	2,237	,025
Day 8-Day 6	,667	4,320	,154	,877
Day 8-Day 2	6,000	4,320	1,389	,165
Day 8-Day 0	9,000	4,320	2,083	,037
Day 6-Day 2	5,333	4,320	1,234	,217
Day 6-Day 0	8,333	4,320	1,929	,054
Day 2-Day 0	3,000	4,320	,694	,487

MT texture results

Sample 1-Sample 2	Test Statistic	Std. Error	Std. Test Statistic	Sig.
Day 10-Day 8	4,667	4,336	1,076	,282
Day 10-Day 6	4,833	4,336	1,115	,265
Day 10-Day 4	8,500	4,336	1,960	,050
Day 10-Day 2	12,000	4,336	2,767	,006
Day 10-Day 0	15,000	4,336	3,459	<,001
Day 8-Day 6	,167	4,336	,038	,969
Day 8-Day 4	3,833	4,336	,884	,377
Day 8-Day 2	7,333	4,336	1,691	,091
Day 8-Day 0	10,333	4,336	2,383	,017
Day 6-Day 4	3,667	4,336	,846	,398
Day 6-Day 2	7,167	4,336	1,653	,098
Day 6-Day 0	10,167	4,336	2,345	,019
Day 4-Day 2	3,500	4,336	,807	,420
Day 4-Day 0	6,500	4,336	1,499	,134
Day 2-Day 0	3,000	4,336	,692	,489

LAP texture results

Sample 1-Sample 2	Test Statistic	Std. Error	Std. Test Statistic	Sig.
Day 10-Day 8	4,333	4,330	1,001	,317
Day 10-Day 6	6,000	4,330	1,386	,166
Day 10-Day 4	7,667	4,330	1,771	,077
Day 10-Day 2	12,000	4,330	2,772	,006
Day 10-Day 0	15,000	4,330	3,465	<,001

Day 8-Day 6	1,667	4,330	,385	,700
Day 8-Day 4	3,333	4,330	,770	,441
Day 8-Day 2	7,667	4,330	1,771	,077
Day 8-Day 0	10,667	4,330	2,464	,014
Day 6-Day 4	1,667	4,330	,385	,700
Day 6-Day 2	6,000	4,330	1,386	,166
Day 6-Day 0	9,000	4,330	2,079	,038
Day 4-Day 2	4,333	4,330	1,001	,317
Day 4-Day 0	7,333	4,330	1,694	,090
Day 2-Day 0	3,000	4,330	,693	,488

TVC

TVC across samples

Supplementary Table 7 TVC across samples

Hypothesis Test Summary

	Null Hypothesis	Test	Sig.[a,b]	Decision
1	The distribution of LDPE_TVC is the same across categories of TVC_Days.	Independent-Samples Kruskal-Wallis Test	,006	Reject the null hypothesis.
2	The distribution of LDPE_15EG@Lap_TVC is the same across categories of TVC_Days.	Independent-Samples Kruskal-Wallis Test	,007	Reject the null hypothesis.
3	The distribution of LDPE_15EG@Mt_TVC is the same across categories of TVC_Days.	Independent-Samples Kruskal-Wallis Test	,009	Reject the null hypothesis.

a. The significance level is ,050.

b. Asymptotic significance is displayed.

LDPE TVC across days

Sample 1-Sample 2	Test Statistic	Std. Error	Std. Test Statistic	Sig.
Day 0-Day 2	-3,000	4,359	-,688	,491
Day 0-Day 4	-6,000	4,359	-1,376	,169
Day 0-Day 6	-9,000	4,359	-2,065	,039
Day 0-Day 8	-13,000	4,359	-2,982	,003
Day 0-Day 10	-14,000	4,359	-3,212	,001
Day 2-Day 4	-3,000	4,359	-,688	,491
Day 2-Day 6	-6,000	4,359	-1,376	,169
Day 2-Day 8	-10,000	4,359	-2,294	,022
Day 2-Day 10	-11,000	4,359	-2,524	,012
Day 4-Day 6	-3,000	4,359	-,688	,491
Day 4-Day 8	-7,000	4,359	-1,606	,108
Day 4-Day 10	-8,000	4,359	-1,835	,066
Day 6-Day 8	-4,000	4,359	-,918	,359
Day 6-Day 10	-5,000	4,359	-1,147	,251
Day 8-Day 10	-1,000	4,359	-,229	,819

LDPE_15EG@Lap_TVC across Days

Sample 1-Sample 2	Test Statistic	Std. Error	Std. Test Statistic	Sig.
Day 0-Day 4	-3,000	4,359	-,688	,491
Day 0-Day 2	-6,667	4,359	-1,529	,126

Day 0-Day 6	-8,333	4,359	-1,912	,056
Day 0-Day 8	-12,333	4,359	-2,829	,005
Day 0-Day 10	-14,667	4,359	-3,365	<,001
Day 4-Day 2	3,667	4,359	,841	,400
Day 4-Day 6	-5,333	4,359	-1,224	,221
Day 4-Day 8	-9,333	4,359	-2,141	,032
Day 4-Day 10	-11,667	4,359	-2,677	,007
Day 2-Day 6	-1,667	4,359	-,382	,702
Day 2-Day 8	-5,667	4,359	-1,300	,194
Day 2-Day 10	-8,000	4,359	-1,835	,066
Day 6-Day 8	-4,000	4,359	-,918	,359
Day 6-Day 10	-6,333	4,359	-1,453	,146
Day 8-Day 10	-2,333	4,359	-,535	,592

LDPE_15EG@Mt_TVC across Days

Sample 1-Sample 2	Test Statistic	Std. Error	Std. Test Statistic	Sig.
Day 0-Day 2	-4,667	4,357	-1,071	,284
Day 0-Day 4	-5,333	4,357	-1,224	,221
Day 0-Day 6	-8,000	4,357	-1,836	,066
Day 0-Day 8	-12,000	4,357	-2,754	,006
Day 0-Day 10	-15,000	4,357	-3,443	<,001
Day 2-Day 4	-,667	4,357	-,153	,878
Day 2-Day 6	-3,333	4,357	-,765	,444
Day 2-Day 8	-7,333	4,357	-1,683	,092
Day 2-Day 10	-10,333	4,357	-2,372	,018

Day 4-Day 6	-2,667	4,357	-,612	,540
Day 4-Day 8	-6,667	4,357	-1,530	,126
Day 4-Day 10	-9,667	4,357	-2,219	,026
Day 6-Day 8	-4,000	4,357	-,918	,359
Day 6-Day 10	-7,000	4,357	-1,607	,108
Day 8-Day 10	-3,000	4,357	-,689	,491

TVC across days

Supplementary Table 8 TVC across days

Hypothesis Test Summary

	Null Hypothesis	Test	Sig.[a,b]	Decision
1	The distribution of Day_0_TVC is the same across categories of TVC_Samples.	Independent-Samples Kruskal-Wallis Test	1,000	Retain the null hypothesis.
2	The distribution of Day_2_TVC is the same across categories of TVC_Samples.	Independent-Samples Kruskal-Wallis Test	,115	Retain the null hypothesis.
3	The distribution of Day_4_TVC is the same across categories of TVC_Samples.	Independent-Samples Kruskal-Wallis Test	,061	Retain the null hypothesis.
4	The distribution of Day_6_TVC is the same across categories of TVC_Samples.	Independent-Samples Kruskal-Wallis Test	,044	Reject the null hypothesis.
5	The distribution of Day_8_TVC is the same across categories of TVC_Samples.	Independent-Samples Kruskal-Wallis Test	,046	Reject the null hypothesis.
6	The distribution of Day_10_TVC is the same across categories of TVC_Samples.	Independent-Samples Kruskal-Wallis Test	,051	Retain the null hypothesis.

a. The significance level is ,050.

b. Asymptotic significance is displayed.

Days across Samples

Day 0

Sample 1-Sample 2	Test Statistic	Std. Error	Std. Test Statistic	Sig.
LDPE-LDPE/15EG@Mt	,000	2,121	,000	1,000
LDPE-LDPE/15EG@Lap	,000	2,121	,000	1,000
LDPE/15EG@Mt-LDPE/15EG@Lap	,000	2,121	,000	1,000

Day 2

Sample 1-Sample 2	Test Statistic	Std. Error	Std. Test Statistic	Sig.
LDPE/15EG@Lap-LDPE/15EG@Mt	3,833	2,227	1,722	,085
LDPE/15EG@Lap-LDPE	4,167	2,227	1,871	,061
LDPE/15EG@Mt-LDPE	,333	2,227	,150	,881

Day 4

Sample 1-Sample 2	Test Statistic	Std. Error	Std. Test Statistic	Sig.
LDPE/15EG@Mt-LDPE/15EG@Lap	-1,000	2,236	-,447	,655
LDPE/15EG@Mt-LDPE	5,000	2,236	2,236	,025
LDPE/15EG@Lap-LDPE	4,000	2,236	1,789	,074

Day 6

Sample 1-Sample 2	Test Statistic	Std. Error	Std. Test Statistic	Sig.
LDPE/15EG@Lap-LDPE/15EG@Mt	2,000	2,227	,898	,369
LDPE/15EG@Lap-LDPE	5,500	2,227	2,470	,014
LDPE/15EG@Mt-LDPE	3,500	2,227	1,572	,116

Day 8

Sample 1-Sample 2	Test Statistic	Std. Error	Std. Test Statistic	Sig.
LDPE/15EG@Lap-LDPE/15EG@Mt	1,667	2,198	,758	,448
LDPE/15EG@Lap-LDPE	5,333	2,198	2,426	,015
LDPE/15EG@Mt-LDPE	3,667	2,198	1,668	,095

Day 10

Sample 1-Sample 2	Test Statistic	Std. Error	Std. Test Statistic	Sig.
LDPE/15EG@Lap-LDPE/15EG@Mt	3,667	2,236	1,640	,101
LDPE/15EG@Lap-LDPE	5,333	2,236	2,385	,017
LDPE/15EG@Mt-LDPE	1,667	2,236	,745	,456
